# Integrative taxonomy reveals hidden fungal and algal diversity in foliicolous *Porinaceae* (*Ascomycota*, *Ostropales*) from Madagascar and the Indian Ocean Islands

**DOI:** 10.3897/imafungus.17.207656

**Published:** 2026-11-05

**Authors:** Sandro Debaene, Luca Borgato, Damien Ertz

**Affiliations:** 1 Meise Botanic Garden, Research Department, Nieuwelaan 38, 1860 Meise, Belgium Meise Botanic Garden, Research Department Meise Belgium; 2 Fédération Wallonie–Bruxelles, Service général de l'Enseignement supérieur et de la Recherche scientifique, rue A. Lavallée 1, 1080 Bruxelles, Belgium Fédération Wallonie–Bruxelles, Service général de l'Enseignement supérieur et de la Recherche scientifique Bruxelles Belgium

**Keywords:** Biogeography, endemism, multilocus phylogeny, mycobiont, Paleotropics, photobiont, phylogeography, species delimitation

## Abstract

*Porinaceae* is one of the most common families of foliicolous lichens in tropical regions. Despite its abundance, major gaps remain in our understanding of its diversity and phylogeny. This study addresses these knowledge gaps and examines photobiont selectivity in Madagascar and the Indian Ocean Islands. In total, 114 lichen specimens from Madagascar and the neighbouring islands of Mauritius and Mayotte were sequenced. Mycobiont diversity was investigated using the mtSSU and ITS regions, while photobiont diversity and selectivity were assessed using the *rbc*L marker. Phenotypic traits, including ascospore and perithecial characteristics, were evaluated alongside molecular data to support species delimitation. Thirty-seven *Porinaceae* taxa were recognised using an integrative taxonomic approach, including six species newly described here: *Porina cincta* Debaene & Ertz, *P. complanata* Debaene & Ertz, *P. madagascariensis* Debaene & Ertz, *P. mayottensis* Debaene & Ertz, *P. parva* Debaene, Diederich & Ertz, and *P. trichocoronata* Debaene & Ertz. The *rbc*L data recovered 25 algal haplotypes distributed across six major clades, four of which were newly recovered. Despite limited morphological differentiation, the mycobiont exhibited unexpectedly high genetic diversity, and some traditionally diagnostic characters, such as ascospore septation, proved to be unreliable at times. These patterns were accompanied by strong phylogeographic structuring and high levels of island endemism. Bipartite network analyses revealed complex symbiotic interactions, with both highly specific and more flexible mycobiont-photobiont associations, alongside substantial photobiont diversity. Altogether, this study emphasises the need for extensive DNA multilocus sampling and type-linked material, combined with thorough phenotypic assessments, to refine species and genus concepts within *Porinaceae*.

## Introduction

Foliicolous lichens are defined by their ecological preference rather than by systematic coherence. They inhabit the surfaces of living leaves, especially in the understorey of tropical rainforests, where they form a characteristic component of the lichen vegetation. This ecological strategy evolved independently multiple times across lichen families, resulting in considerable anatomical diversity despite the shared epiphyllous lifestyle. Due to their ephemeral substrates, foliicolous lichens invest relatively little in biomass and instead emphasise rapid and abundant sexual reproduction, typically producing thin, crustose thalli paired with rapidly formed dispersal units (Lücking 2001, 2008; Baloch and Grube 2009). Almost 1000 foliicolous species are currently known worldwide, with the highest diversity in primary tropical lowland rainforests and a decline with altitude and seasonality (Lücking 2008; Ward et al. 2026). Despite their high diversity, research effort has been unevenly distributed geographically, with foliicolous lichens having been studied more extensively in the Neotropics than in the Paleotropics (Herrera-Campos et al. 2004; Lücking 2008). However, recent global phylogenetic work on dominant foliicolous lineages has begun to rebalance this picture by incorporating multiple tropical hotspots, including the Indian Ocean region (Lebreton et al. 2025).

*Porinaceae* Rchb. is a highly diverse lichen family comprising approximately 400 accepted species, around 35% of which are foliicolous, and is among the most abundant foliicolous lichen groups in tropical forests (Grube et al. 2004; Lücking 2004; Baloch and Grube 2006, 2009; McCarthy 2013; Lücking et al. 2017, 2020; Sobreira et al. 2018). The diagnostic features include a crustose thallus, frequently containing calcium oxalate crystals, and usually lacking secondary metabolites. The ascomata are perithecioid; paraphyses are unbranched, asci are unitunicate with 8 spores, commonly featuring a chitinoid ring at the tip. The ascospores are hyaline, mostly transversely septate, and rarely muriform (Baloch and Grube 2006; Lücking 2008; Ertz and Diederich 2022). The photobiont is a trentepohlioid alga. Since these algae take on a different form, with reduced or absent reproductive structures in symbiosis, identifying the exact species is often difficult without molecular methods (Hametner et al. 2014; Klimešová et al. 2019). The photobiont in foliicolous *Porinaceae* is usually categorised as either rectangular algal cells in radiating plates or angular-rounded cells arranged irregularly, leaving more or less interspace between them (Baloch and Grube 2006; Lücking 2008; Nelsen et al. 2011).

Due to the high number of *Porinaceae* species and the fact that most are still classified within the paraphyletic genus *Porina* Ach., many researchers have tried to subdivide the family into smaller genera. Despite numerous studies and an extensive number of species, no consensus has been reached (McCarthy 1995; McCarthy and Malcolm 1997; Sobreira et al. 2018; Lücking et al. 2020; Ertz and Diederich 2022; Lebreton et al. 2024). Most efforts to establish a generic framework and define species depend mainly on traditional features such as the presence of a crystal layer in the perithecia (crystallostratum), pigmentation of the perithecia and their inner layers, presence of setae, ascospore morphology, and thallus structure. A major issue is that many of these characters exhibit gradual transitions and/or lack monophyletic support, therefore complicating genus and species delimitation (McCarthy 1995; McCarthy and Malcolm 1997; Baloch and Grube 2006; Sobreira et al. 2018; Lebreton et al. 2024). For instance, species with black pigmented perithecia cluster into at least three unrelated groups: *Trichothelium* Müll.Arg., *Pseudosagedia* (Müll.Arg.) Choisy, and *Porina byssophila* (Körb. ex Hepp) Zahlbr. (Sobreira et al. 2018). Recently, a new clade including *Porina nuculoides* Ertz & Diederich and *Porina rupicola* Ertz & Diederich was identified. It features perithecia with a crystallostratum distinct from that of other clades sharing this trait, an unexpected finding, since species with a crystallostratum have traditionally been considered part of a single early-diverging lineage (Grube et al. 2004; Ertz and Diederich 2022).

Recent phylogenetic studies have substantially enhanced the understanding of relationships within *Porinaceae*. Evidence indicates that several traditional generic circumscriptions do not accurately reflect evolutionary history. As a result, the delimitation of genera and species increasingly relies on molecular phylogenetic evidence, combined with morphological characteristics. This integrative approach has produced the recognition of several new genera within the broadly paraphyletic genus *Porina*. Sobreira et al. (2018) described the genus *Flabelloporina* Sobreira, M. Cáceres & Lücking, characterised by flabelliform squamules. Subsequently, Lücking et al. (2020) added *Saxiloba* Lücking, Moncada & Viñas, distinguished by a placodioid thallus and fenestrate anatomy, and recently Lebreton et al. (2024) identified *Gallaicolichen* Sérus. & Lücking as part of *Porinaceae*. Despite these advances, most molecular phylogenies rely on a single marker (mtSSU) due to difficulties in sequencing nuclear rDNA regions and focus heavily on neotropical regions and taxa (Sobreira et al. 2018; Lücking et al. 2020; Sanders et al. 2023; Lebreton et al. 2024). Moreover, many species have yet to be sequenced, and many available sequences originate from non-type material or different locations than where the species was originally described, reducing confidence in their identification. There are also very few studies examining photobiont diversity and selectivity within the family, and almost none specifically focused on foliicolous species (Nelsen et al. 2011; Borgato et al. 2022; Sanders et al. 2023). This overall lack of reliable molecular data makes it difficult to evaluate species and genus concepts, limiting insights into biogeographic and symbiotic patterns within *Porinaceae*.

Like other parts of the Paleotropics, Madagascar and the Indian Ocean Islands (MIOI) remain poorly studied regarding the diversity and phylogeny of foliicolous *Porinaceae*. A checklist of the lichens from Madagascar has been published by Aptroot (2016), from the Seychelles by Seaward and Aptroot (2006, 2009), and Diederich and Ertz (2020) provided a checklist from Mauritius. Following this, Ertz and Diederich (2022) conducted a study of the diversity of *Porina* species in Mauritius. Recent research suggests a considerably higher level of endemism among lichens on oceanic islands than previously thought, emphasising the potential for undocumented diversity on island systems (Simon et al. 2018; Lücking et al. 2020; Masson et al. 2024; Lebreton et al. 2025). To address this knowledge gap, this paper focuses on the foliicolous *Porinaceae* of MIOI. Its goals are to (1) document the diversity in the region, (2) expand molecular sampling and improve phylogenetic understanding of the family, (3) use an integrative approach to species delimitation combining morphological and molecular evidence, and (4) examine photobiont diversity and their mycobiont selectivity.

## Methods

### Specimens

Collections originating from Madagascar (2014, 2019, and 2022), Mauritius (2019), and Mayotte (2017) were studied (see Suppl. materials 1, 2). Specimens are deposited in herbaria BR, TAN, MAU, and MAO. All specimens examined in this study were collected by D. Ertz, and collecting numbers are therefore cited without the collector’s name throughout the manuscript.

### Morphology

The external morphology of the specimens was described and measured using an Olympus SZX12 stereomicroscope. All macroscopic images of the lichens were captured with a Keyence VHX-5000 digital microscope and a VH-Z20R/W/T lens by S. Debaene. Hand-cut sections and squash preparations of perithecia and thalli were mounted in water and 5% aqueous potassium hydroxide (K) and examined under an Olympus BX51 compound microscope. K solutions were typically added to water mounts by placing a drop at the edge of the coverslip. The ascus apical ring was identified using a 1% ammoniacal Congo Red solution. Measurements from perithecial sections (such as asci and ascospores) are reported in water. For ascospores, photobiont cells, and asci (when n > 30), data are presented as (minimum–) x̄ - SD – x̄ + SD (–maximum), followed by the number of measurements (n). Measurements of perithecia, asci (if n < 30), periostiolar areas, etc., are given as minimum–maximum with the number of measurements (n), if relevant. Ascospore measurements exclude the perispore. Perithecia were measured at their widest point, including the base, for width, and from the apex to its basal insertion in the thallus for height. Only mature perithecia, asci, and ascospores were measured. Perithecia were measured to the nearest 50 µm in width and to 10 µm in height; perithecial inner layers to 5 µm; asci and photobiont cells to 1 µm; and ascospore length and width to 0.5 µm and 0.1 µm, respectively. Micrographs were taken with an Olympus BX51 microscope equipped with an Olympus SC50 digital camera. Lichen secondary metabolites were not tested, as *Porinaceae* and foliicolous lichens generally lack acetone-soluble compounds detectable by thin-layer chromatography (TLC) (Lücking 2008; Ertz and Diederich 2022).

### Molecular analyses

Well–preserved specimens, kept in the freezer (ca. -20 °C) after collection, were used for DNA sampling. Hand-cut sections of the perithecia and/or the thallus were used for PCRs. Before sampling, the sections were examined to exclude visible contamination by other fungi, free-living algae, or other extraneous material. Direct extractions, which represent crude DNA template preparations, were first made directly from the material. The hand-cut material was first put in microtubes with 40 µL of H_2_O, 5 µL of BSA, and 5 µL of DNA polymerase buffer. Extractions were then performed on a T100 thermocycler (Bio–Rad, Hercules, CA, USA) with the following cycling parameters: 95 °C (10 min), followed by 40 cycles of 95 °C (45 s) and 65 °C (30 s). The resulting extract was then used crude in all subsequent PCRs. For all species, we tried to amplify a targeted DNA fragment of ca. 0.8 kb coding for the small subunit of the mitochondrial ribosome (mtSSU) using primers mrSSU1 and mrSSU3R (Zoller et al. 1999), ca. 0.6–1 kb of the nuclear ribosomal internal transcribed spacer (ITS1 + 5.8S + ITS2) using primers ITS1F (Gardes and Bruns 1993) and ITSPoR (Orange et al. 2020), and ca. 0.8 kb of the large subunit of ribulose-1,5-biphosphate carboxylase (*rbc*L) locus in the alga’s chloroplast using primers *rbc*L-203F and *rbc*L-991R (Nelsen et al. 2011) to check the identity of the photobiont. For the mtSSU marker, a 20 µL PCR mix was used containing 7.5 µL water, 8 µL of DNA extract, 1.2 µL 10 × PCR buffer, 1.6 µL of dNTPs (2.5 mM of each), 0.8 µL of BSA (2.5 mg **·** mL^-1^), 0.4 µL of each primer (20 µM), and 0.5 U (0.1 µL) of DreamTaq DNA Polymerase (Thermo Fisher Scientific, Waltham, MA, USA; Cat. no. EP0701). The same mix was used for the *rbc*L marker, but 1.6 µL of MgCl_2_ (25 µM) was added to the mix and subtracted from the water volume. Because secondary structures within the ITS region were suspected of interfering with PCR amplification, an alternative approach was employed. A 20 µL PCR mix was used containing 6 µL of DNA extract, 5.7 µL of Trehalose (20%), 1 µL of DMSO (50%), 4 µL 5 × KAPA2G GC Buffer (Roche, Basel, Zwitzerland; Cat. no. 08200114001), 1.4 µL of dNTPs (2.5 mM of each), 1 µL of BSA (2.5 mg **·** mL^-1^), 0.4 µL of each primer (20 µM), and 0.5 U (0.1 µL) of KAPA Biosystems Taq DNA Polymerase (Roche, Basel, Switzerland; Cat. no. 07958463001). PCRs were run on a T100 thermocycler (Bio–Rad, Hercules, CA, USA). A touchdown PCR program was used to amplify all DNA fragments. For the mtSSU marker the following cycling parameters were used: 95 °C (3 min), six cycles of 95 °C (45 s), 55–52.5 °C (30 s), 72 °C (1 min 15 s), 36 cycles of 95 °C (45 s), 52 °C (1 min), 72 °C (1 min 15 s), and a final extension of 72 °C (7 min). For the *rbc*L marker the following cycling parameters were used: 95 °C (3 min), ten cycles of 95 °C (45 s), 52–47.5 °C (1 min), 72 °C (1 min), 36 cycles of 95 °C (45 s), 47 °C (1 min), 72 °C (1 min), and a final extension of 72 °C (7 min). For the ITS marker the following cycling parameters were used: 95 °C (3 min), ten cycles of 95 °C (45 s), 55–53.2 °C (1 min), 72 °C (1 min 30 s), 36 cycles of 95 °C (45 s), 47 °C (1 min), 72 °C (1 min 30 s), and a final extension of 72 °C (7 min). PCR yields were verified by running the products on a Fragment Analyser (Advanced Analytical, Parkersburg, WV, USA). Both strands were sequenced by Macrogen Europe B.V. (Amsterdam, The Netherlands) using the amplification primers. Sequence fragments were assembled in Geneious Prime® v.2024.0 (Biomatters Ltd., Auckland, New Zealand). The sequence identities were assessed using BLAST (Altschul et al. 1990) against the core_nt database on the NCBI website.

### Phylogenetic analyses

Datasets of mtSSU and ITS sequences for mycobionts and *rbc*L sequences for photobionts were compiled to study their phylogenetic relationships within the family *Porinaceae* and with their algal partners (see Suppl. materials 1, 2). Sequences from GenBank for *Porinaceae* (mtSSU and ITS) and *Trentepohliaceae* Hansgirg (*rbc*L) were included to enhance taxon sampling for both the fungi and algae. Phylogenetic analyses were conducted separately for each locus (mtSSU, ITS, and *rbc*L). Sequences were aligned using MAFFT v.7.402 (Katoh et al. 2002) on the CIPRES Web Portal (Miller et al. 2010) and manually inspected and adjusted using AliView v.1.31 (Larsson 2014). *Coenogonium leprieurii* (Mont.) Nyl. (AY584698; Lutzoni et al. 2004), *C. luteum* (Dicks.) Kalb & Lücking (AY584699; Lutzoni et al. 2004), and *C. pineti* (Ach.) Lücking & Lumbsch (AY300884; Lumbsch et al. 2003) were selected as outgroup taxa for the mtSSU dataset, following Sobreira et al. (2018). *Porina austroatlantica* P.M. McCarthy & Fryday (MN687933; Orange et al. 2020) and *Porina multipuncta* (Coppins & P. James) Ertz et al. (MN687934 and MN687935; Orange et al. 2020) were selected as outgroups for the ITS dataset, according to Orange et al. (2020). For the *rbc*L dataset, the species *Caulerpa prolifera* (Forssk.) J.V.Lamour. (AY942173; Lam and Zechman 2006), *Halimeda opuntia* (L.) J.V.Lamour. (AY942174; Lam and Zechman 2006), and *Ulva linza* L. (AB097620; Shimada et al. 2003) were selected as outgroups, following Borgato et al. (2022). Terminal ends of all marker matrices were trimmed, and for the mtSSU and ITS matrices, ambiguous regions were manually delimited and excluded from the alignment, whereas no regions were excluded from the *rbc*L matrix. Consequently, a total of 870 of the 1041 positions were conserved in the mtSSU matrix, 623 of the 677 positions in the ITS matrix, and all 785 positions in the *rbc*L matrix (see Suppl. materials 6–8). The best-scoring maximum-likelihood tree was inferred, and node support was estimated from 1,000 bootstrap replicates in the same run using RAxML-HPC2 v. 8.2.12 (Stamatakis 2014), with the GTR-GAMMA model and default settings as implemented on the CIPRES portal (Miller et al. 2010). Phylogenetic trees were visualised with FigTree v.1.4.2 (Rambaut 2012) and edited with Adobe Illustrator v. 30 (Adobe Inc. 2019). Bootstrap support values (BS) ≥ 70% were considered indicative of significant support (Mason-Gamer and Kellogg 1996). A *bipartite* network graph was constructed using the Bipartite Network Analysis program (Greenville 2018) to visualise the associations between mycobionts and photobionts and edited with Adobe Illustrator v. 30 (Adobe Inc. 2019).

### Taxon delimitation

Taxa were delimited using an integrative taxonomic approach (Lücking et al. 2021), combining molecular phylogenetic evidence and phenotypic observations. The phylogenetic placement of the obtained mtSSU and ITS sequences, along with clade support, was evaluated alongside external and anatomical morphological characters. Morphological assessments included thallus morphology (e.g., size, surface structure, crystal incrustation, prothallus development), perithecial morphology (e.g., size, shape, immersion in the thallus, and periostiolar pigmentation), photobiont morphology, ascus characteristics, and ascospore morphology. The obtained morphological data were compared with published species descriptions and identification keys (e.g., Lücking and Vězda 1998; Lücking 2008) for identification or to assess taxonomic affinities. Quantitative morphological data, including measurements of ascospores, perithecia and photobionts, were summarised using descriptive statistics. When two variables were measured on the same structure (e.g., ascospore length and width), both dimensions were considered, and ratios were calculated. The resulting summaries and ratios were interpreted alongside qualitative morphological characters and multilocus phylogenetic evidence to distinguish morphologically similar taxa and to support taxonomic delimitation. No automated single-marker species delimitation method or fixed-threshold molecular operational taxonomic unit (MOTU) clustering approach was used because genetic divergence varied substantially among and within clades and was not consistently associated with phenotypic differentiation. This decision was further supported by the findings of Lebreton et al. (2025), who documented substantial method-dependent variation in species delimitation within a comparable dataset of foliicolous lichens. In particular, they observed a tendency of the Bayesian Poisson Tree Process (bPTP) to oversplit, whereas the Generalised Mixed Yule Coalescent (GMYC) showed limited sensitivity to lineages associated with short branches. They also highlighted the sensitivity of these approaches to geographic sampling and the number of loci analysed. The designation “cf.” was used for specimens showing strong similarity to a described species but for which uncertainty remained, whereas “aff.” was used for specimens showing resemblance to a species while differing in important diagnostic characters. Only sequenced specimens were included in the taxonomic assessments. In cases where important diagnostic characters could not be evaluated, such as specimens containing only immature perithecia, lacking hymenial structures, or having damaged perithecia obscuring relevant characteristics (e.g., setae in *Trichothelium*), the material was treated as *Porina* sp. or *Trichothelium* sp. When unidentified specimens were grouped with identified material in the phylogeny, external morphological characters were evaluated to determine whether the specimens could be assigned to the same taxon. In cases where supported clades consisted exclusively of unidentified specimens, external morphology was used to assess whether the material represented a single taxon or multiple distinct taxa. One bambusicolous specimen was included because its phylogenetic placement was considered relevant to a major clade under study. Although bambusicolous and foliicolous habitats are considered ecologically similar, particularly because of the smooth surface of bamboo culms that reproduces the ecological conditions of a leaf (Santesson 1952), bambusicolous material was treated as a distinct substrate category in the present study. Taxa distribution across islands was visualised using Venny 2.1 (Oliveros 2015). To delimit different photobiont clades and haplotypes (noted as clade:haplotype, e.g., 10:2) for the Bipartite Network Analysis, the same approach used by Borgato et al. (2022) was adopted: the resulting phylogenetic tree was employed to define clades, and sequence variation was inspected manually to distinguish haplotypes within clades. Sequences differing by at least three nucleotides were treated as distinct haplotypes, with each nucleotide substitution and insertion/deletion event counted as one difference. A three-difference criterion, rather than the one-difference criterion used by Borgato et al. (2022), was adopted as a conservative operational threshold to avoid excessive subdivision into haplotypes based on very minor sequence variation and to reduce the potential influence of occasional sequencing errors. This criterion was used solely to define haplotypes for the network analysis and was not intended as a biological species-delimitation threshold.

**Figure 1.**
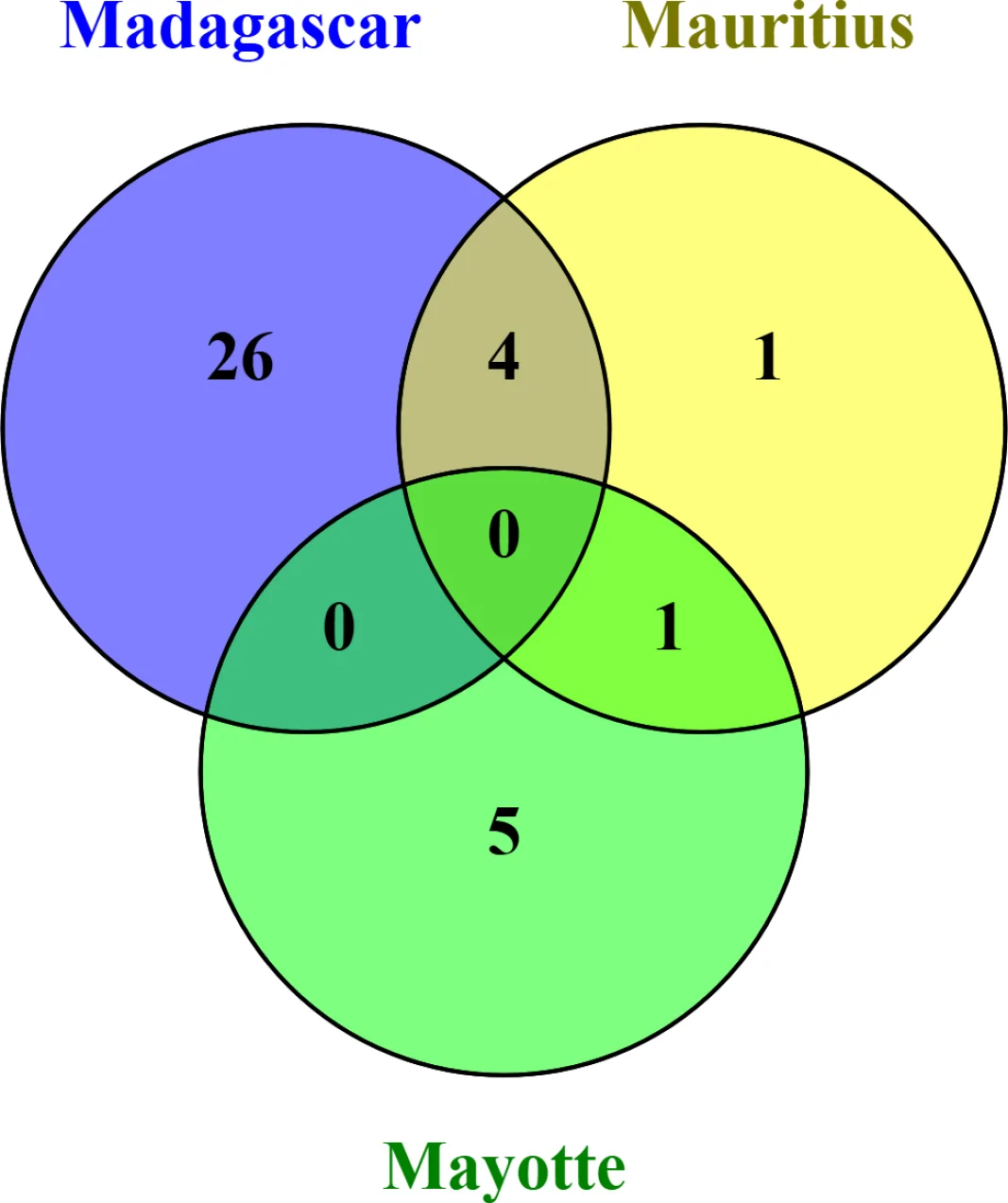
Venn diagram illustrating the number of foliicolous *Porinaceae* taxa across Madagascar, Mauritius, and Mayotte.

## Results

### Taxonomic diversity

A total of 114 *Porinaceae* specimens collected from MIOI were successfully sequenced, yielding both mtSSU and ITS sequences, or, in some cases, only one of them. Thirty-seven taxa were recognised, including six new to science. Overall, 30 taxa were recorded in Madagascar, six in Mauritius, and six in Mayotte (Fig. 1). Thirty-two taxa were restricted to a single island, and only five were shared among islands. None of the taxa were present on all three islands. Madagascar and Mauritius shared four taxa, whereas only a single taxon, *P. rufula* (Kremp.) Vain., was shared between Mauritius and Mayotte. No taxa were shared between Madagascar and Mayotte.

### Phylogenetic analyses

**Figure 2.**
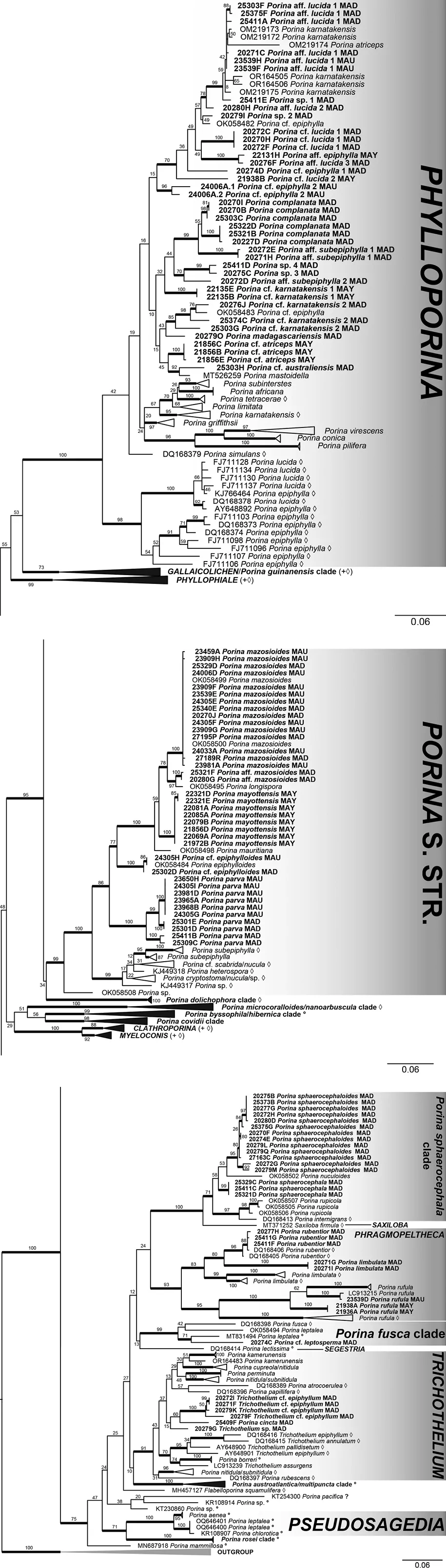
Maximum-Likelihood mtSSU phylogeny of *Porinaceae*. Bootstrap values are shown above or near branches. Branches with bootstrap support ≥ 70% are shown in bold. Newly sequenced specimens are in bold; their names are preceded by their collecting numbers and followed by an abbreviation of their island of origin (MAD = Madagascar, MAU = Mauritius, and MAY = Mayotte). Black triangles indicate collapsed major clades, whereas white triangles indicate collapsed subclades within major clades. Major clades are annotated on the right or collapsed and labelled in bold. The names of specimens originating from the Neotropics are followed by a lozenge (◊). Specimens originating from extratropical regions are followed by a degree sign (°), while those from the Paleotropics are left without a symbol. If the origin is unknown, the names are followed by a question mark (?). Collapsed clades containing specimens from both the Neotropics and the Paleotropics are marked with a (+◊) symbol. See Suppl. material 3 for the complete version of the phylogeny.

**Figure 3.**
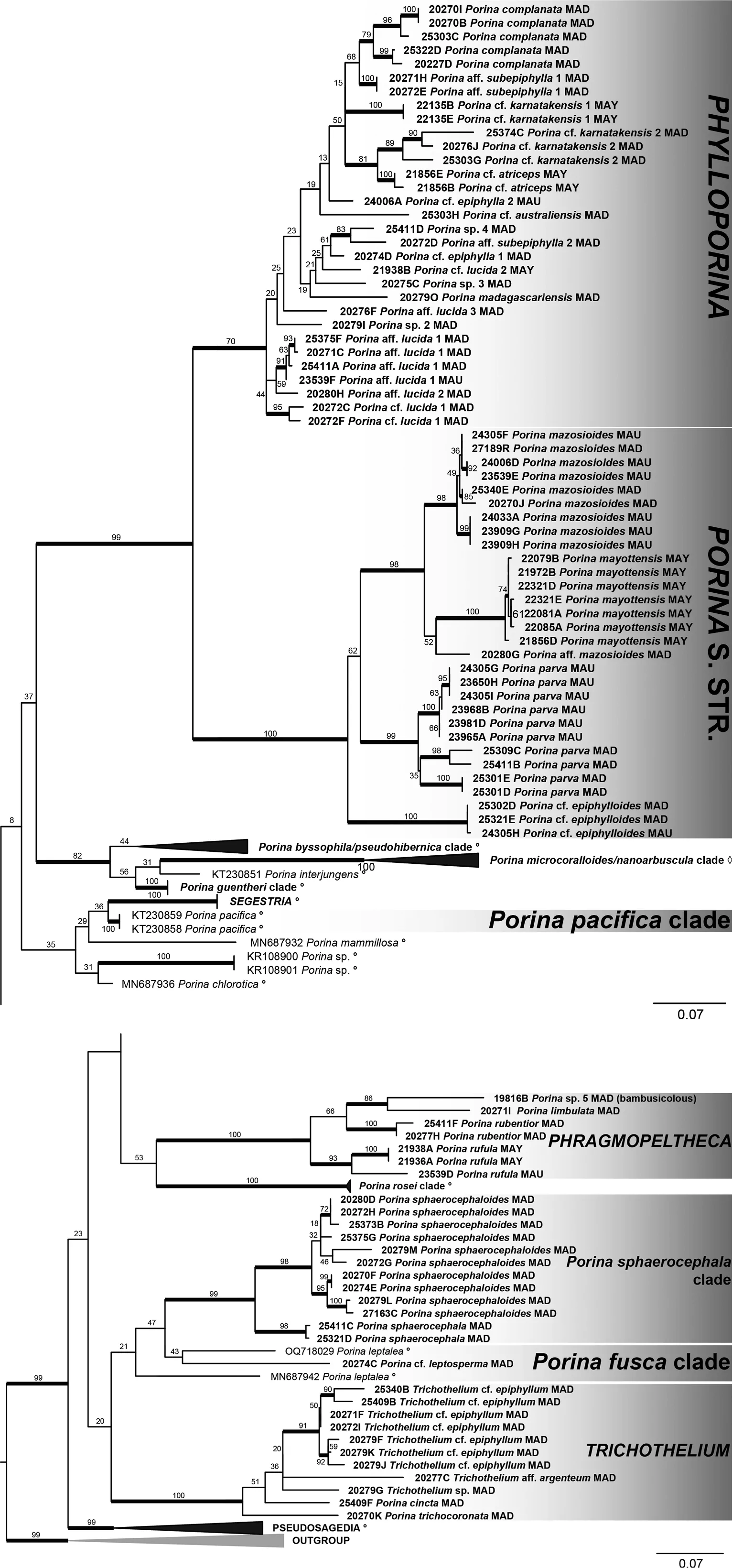
Maximum-Likelihood ITS phylogeny of *Porinaceae*. Bootstrap values are shown above or near branches. Branches with bootstrap support ≥ 70% are shown in bold. Newly sequenced specimens are in bold; their names are preceded by their collecting numbers and followed by an abbreviation of their island of origin (MAD = Madagascar, MAU = Mauritius, and MAY = Mayotte). Major clades are annotated on the right or collapsed and labelled in bold. The names of specimens originating from the Neotropics are followed by a lozenge (◊). Specimens originating from extratropical regions are followed by a degree sign (°), while those from the Paleotropics are left without a symbol. See Suppl. material 4 for the complete version of the phylogeny.

**Figure 4.**
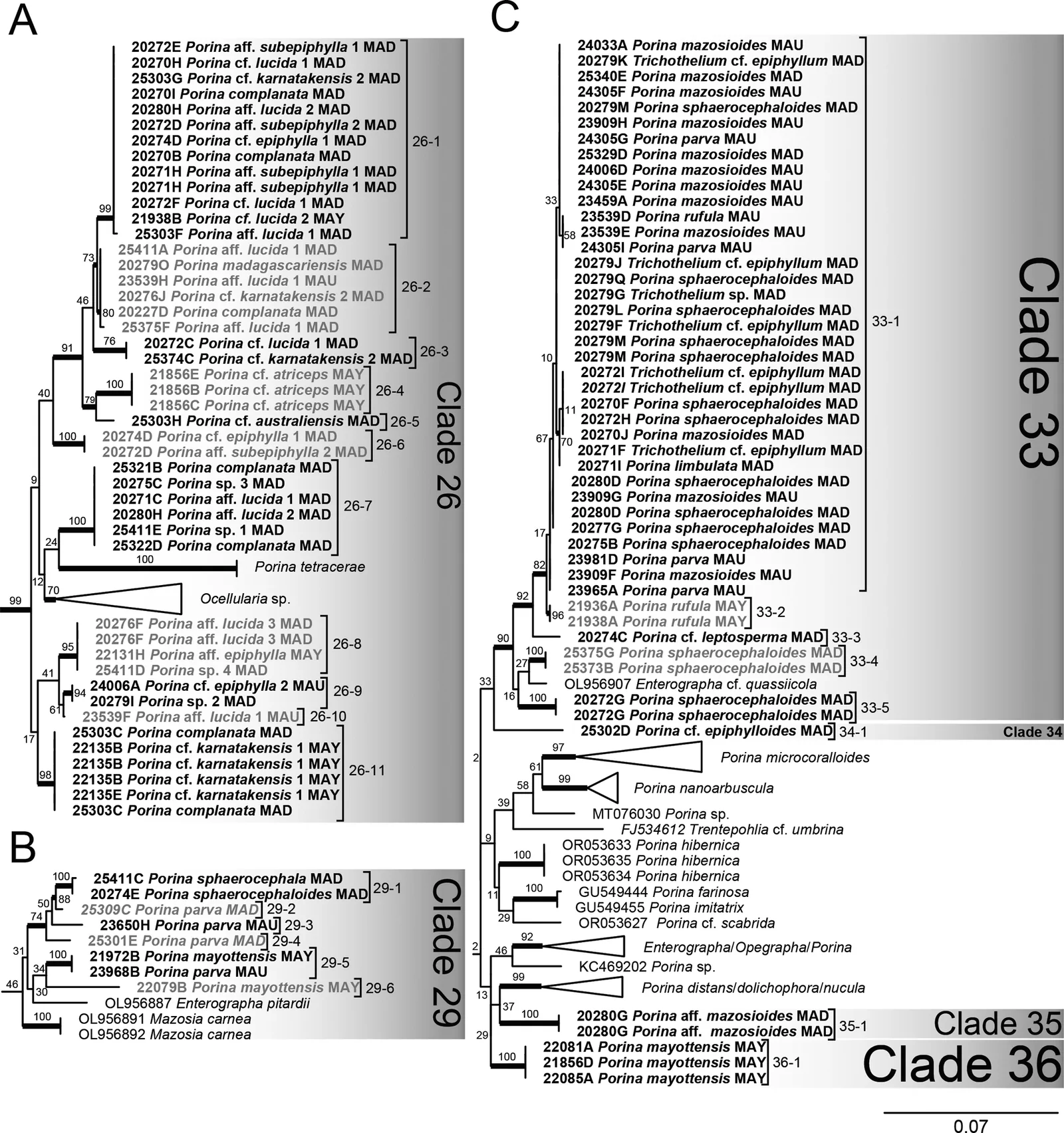
Maximum-Likelihood *rbc*L phylogeny of *Trentepohliaceae* labelled by host. Only the relevant clades are shown. Sequence labels correspond to the host taxon (and specimen), from which the photobiont was sequenced. Bootstrap values are shown above or near branches. Branches with bootstrap support ≥ 70% are shown in bold. Newly sequenced specimens are in bold; their names are preceded by their collecting numbers and followed by an abbreviation of their island of origin (MAD = Madagascar, MAU = Mauritius, and MAY = Mayotte). Algal clades and haplotypes are annotated on the right. Labels of sister haplotypes alternate between black and grey to distinguish adjacent haplotypes. **A** Algal clade 26 and its associated hosts **B** Algal clade 29 and its associated hosts **C** Algal clade 33–36 and their associated hosts. See Suppl. material 5 for the complete version of the phylogeny.

The following numbers of sequences were generated for each marker: 107 for mtSSU, 92 for ITS, and 103 for *rbc*L. The resulting phylogenies are shown in Figs 2, 3, 4. Overall, the topologies were congruent with previous studies.

**mtSSU phylogeny**. (Fig. 2) The same major clades were recovered as in previous studies, with the genus *Porina* paraphyletic and the backbone overall poorly supported (Sobreira et al. 2018; Lücking et al. 2020; Ertz and Diederich 2022; Lebreton et al. 2024). The newly generated sequences were distributed across six major *Porinaceae* clades. Several major clades exhibited phylogeographic structuring, with Paleotropical and Neotropical specimens often forming distinct, well-supported subclades. While some lineages remained pantropical, including specimens from both geographic regions, taxa represented in both the Paleotropics and Neotropics generally showed a phylogenetic trend towards regional segregation. Moreover, several morphologically defined taxa were recovered as non-monophyletic or consisted of multiple genetically distinct lineages. The newly sequenced species *P. sphaerocephala* Vain. and *P. sphaerocephaloides* Farkas were recovered in a major clade sister to the genus *Saxiloba*, representing the first known foliicolous representatives in this part of the phylogenetic tree of the *Porinaceae*.

**ITS phylogeny**. (Fig. 3) Previous ITS phylogenies included only a relatively small number of taxa, so the addition of the newly generated sequences substantially expanded taxonomic coverage (Sanders et al. 2023). Five major known *Porinaceae* clades were sampled for the first time using the ITS marker: the *Phylloporina* clade, the *Porina* s. str. clade, the *Porina sphaerocephala* clade, the *Phragmopeltheca* clade, and the *Trichothelium* clade. The resulting phylogeny was congruent with the mtSSU topology. No conflicts were observed between the two datasets. The ITS phylogeny sometimes provided greater support for relationships among and within taxa, whereas some deeper nodes were less well supported.

***rbc*L phylogeny**. (Fig. 4) The topology and major clades were congruent with those of Borgato et al. (2022), and the clade numbering proposed in that study was retained. Taking into account subsequently published studies, the current *rbc*L framework now comprises 40 recognised *Trentepohliaceae* clades. Clade 29 was the only exception, as it was no longer supported following the inclusion of the newly generated sequences. These recovered sequences were distributed across six major *Trentepohliaceae* clades, four of which are newly reported. The four novel lineages were numbered 33–36, continuing the existing clade numbering established by Borgato et al. (2022). Twenty-five unique haplotypes were identified, revealing substantial diversity in the study area.

### Bipartite network analysis

**Figure 5.**
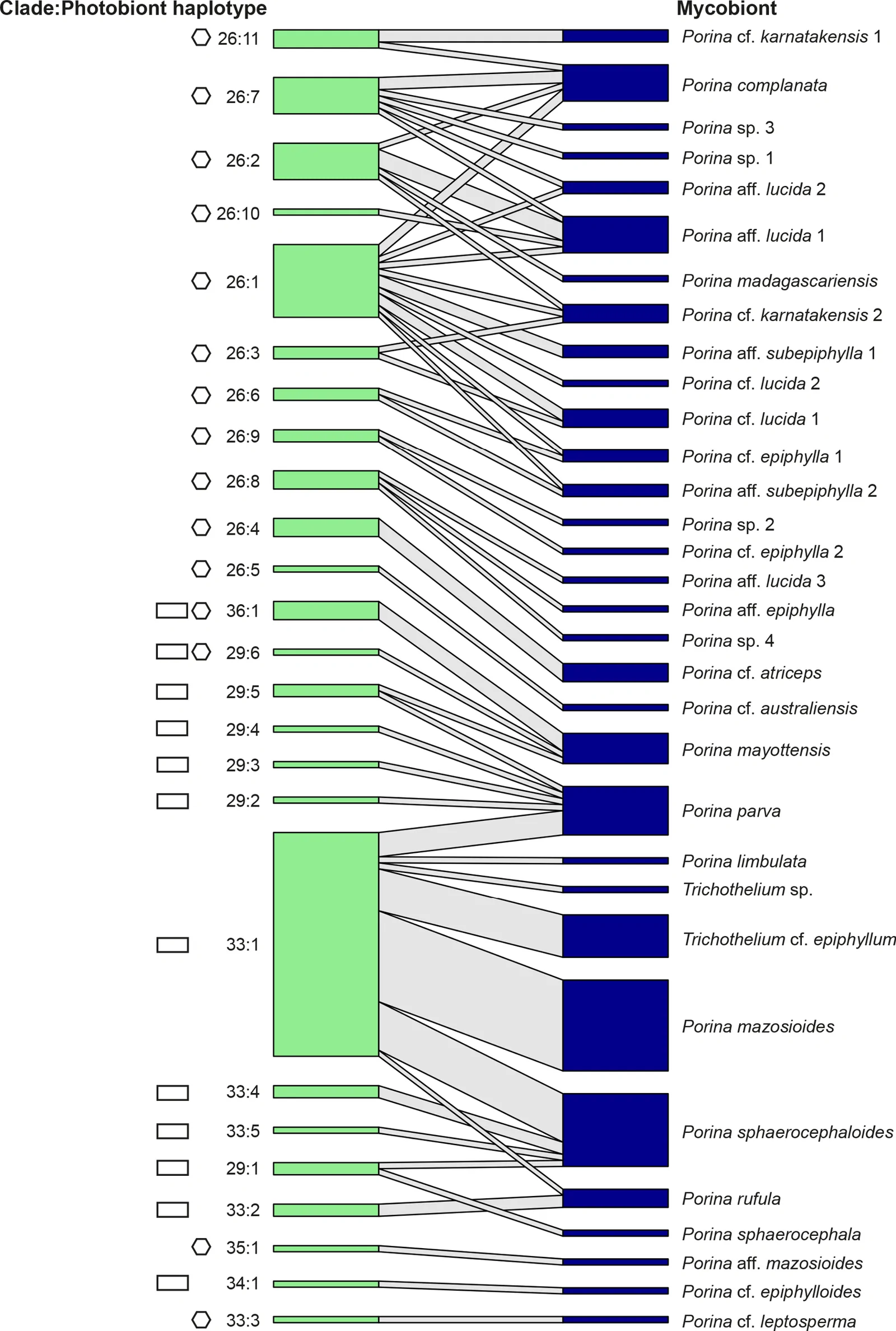
Bipartite interaction network showing associations between photobiont haplotypes and *Porinaceae* taxa. Photobiont haplotypes are shown on the left, while mycobiont taxa are shown on the right. The width of the links is proportional to the number of specimens in the association. The observed photobiont morphotypes are indicated by a symbol next to the algal haplotypes. Hexagons represent angular-rounded cells arranged irregularly, and rectangles represent rectangular cells arranged in radiate plates. When the morphotype associated with a sequenced haplotype is uncertain, both symbols are present.

**Figure 6.**
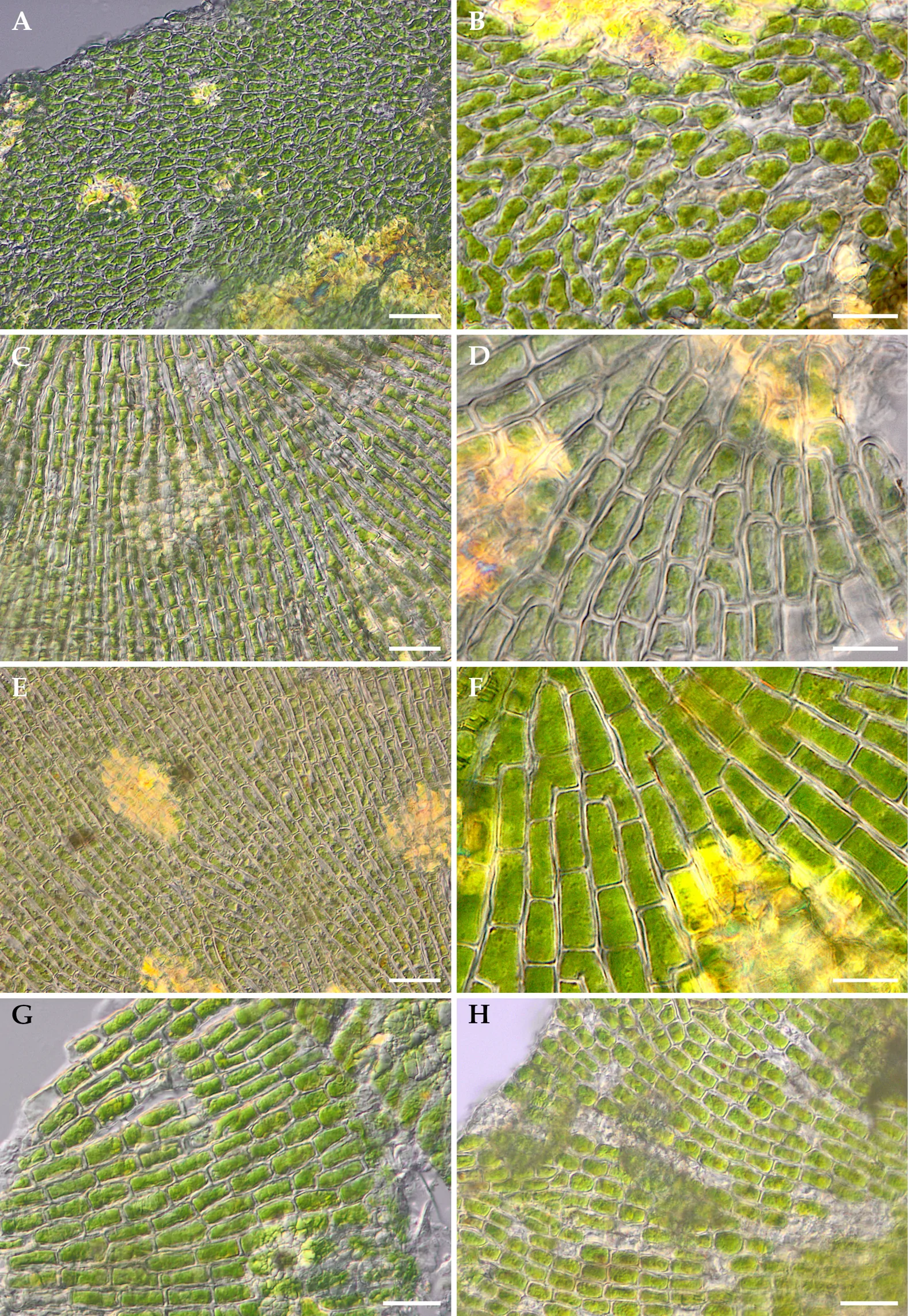
Selected photobiont specimens, in water, representing the morphotypes found across the recovered algal phylogeny. **A** Photobiont from clade 26 associated with *Phylloporina*, haplotype 26:3 (*P. cf. lucida* 1, 20272C), angular-rounded cells arranged irregularly **B** Photobiont from clade 26 associated with *Phylloporina*, haplotype 26:2 (*P. cf. karnatakensis* 2, 20276J), angular-rounded cells arranged irregularly **C** Photobiont from clade 29, haplotype 29:1 (*P. sphaerocephaloides*, 20274E), rectangular cells arranged in radiate plates **D** Photobiont from clade 29, haplotype 29:5 (*P. parva*, 23968B), rectangular cells arranged in radiate plates **E** Photobiont from clade 33, haplotype 33:1 (*P. mazosioides*, 20270J), rectangular cells arranged in radiate plates **F** Photobiont from clade 33, haplotype 33:1 (*P. parva*, 24305I), rectangular cells arranged in radiate plates **G** Photobiont from clade 33, haplotype 33:4 (*P. sphaerocephaloides*, 25375G), rectangular cells arranged in radiate plates **H** Photobiont from clade 33, haplotype 33:5 (*P. sphaerocephaloides*, 20272G), rectangular cells arranged in radiate plates. Scale bars: 20 µm (**A, C, E, G, H**); 10 µm (**B, D, F**).

**Figure 7.**
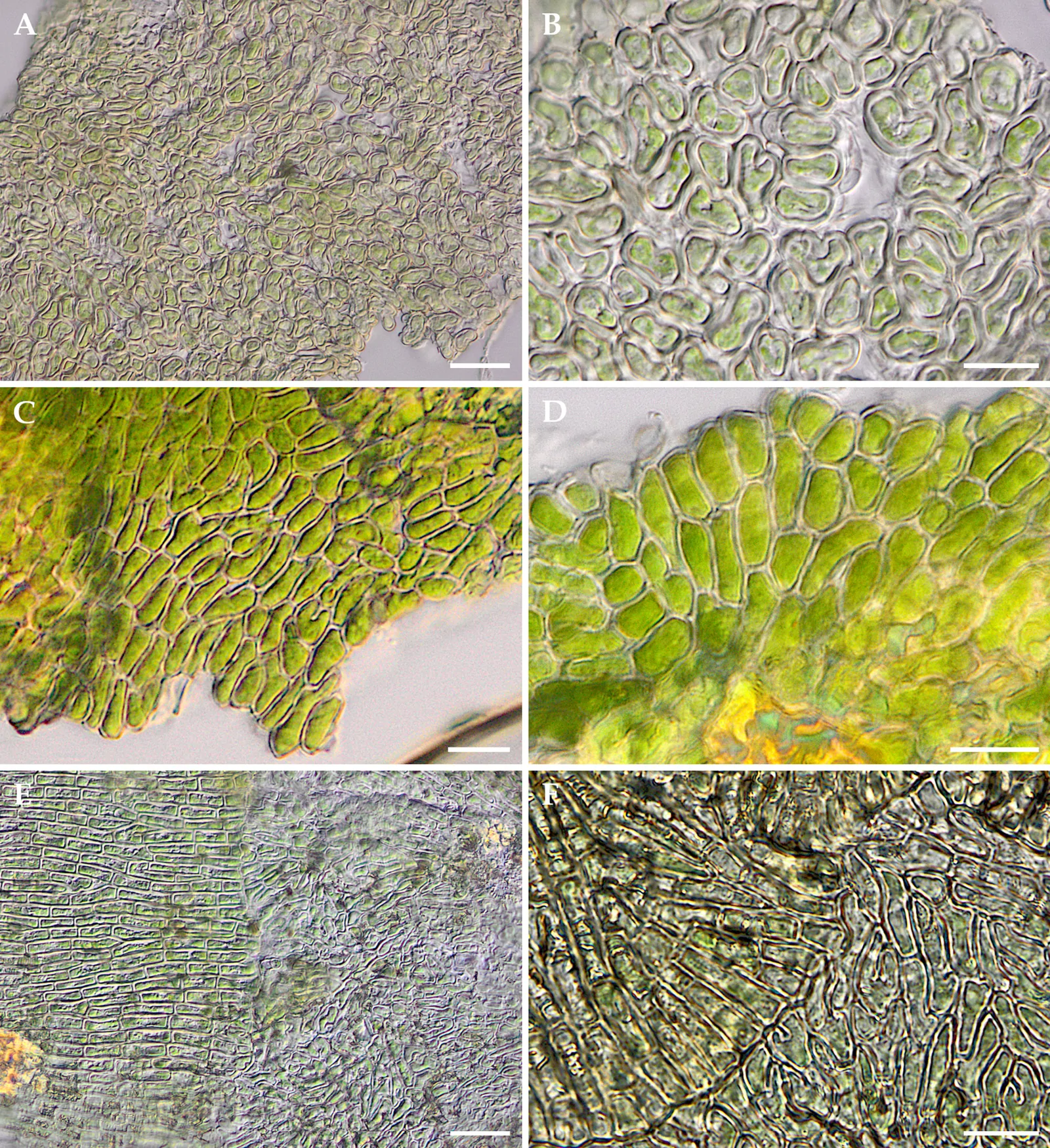
Selected photobiont specimens, in water, representing the morphotypes found across the recovered algal phylogeny. **A** Photobiont from clade 33, haplotype 33:3 (*P. cf. leptosperma*, 20274C), angular-rounded cells arranged irregularly **B** Photobiont from clade 33, haplotype 33:3 (*P. cf. leptosperma*, 20274C), angular-rounded cells arranged irregularly **C** Photobiont from clade 35, haplotype 35:1 (*P. aff. mazosioides*, 20280G), angular-rounded cells arranged irregularly **D** Photobiont from clade 35, haplotype 35:1 (*P. aff. mazosioides*, 20280G), angular-rounded cells arranged irregularly **E** Photobiont from clade 29, haplotype 29:6 (*P. mayottensis*, 22079B), both angular-rounded cells, arranged irregularly, and rectangular cells, arranged in radiate plates, on the same thallus **F** Photobiont from clade 36, haplotype 36:1 (*P. mayottensis*, 22085A), both angular-rounded cells, arranged irregularly, and rectangular cells, arranged in radiate plates, on the same thallus. Scale bars: 20 µm (**A, E**); 10 µm (**B, C, D, F**).

At least one *rbc*L sequence was recovered for the photobiont of 32 of the 37 identified *Porinaceae* taxa (Fig. 5). Multiple associations were observed in both symbiotic partners, with eleven fungal taxa associating with more than one algal haplotype and eleven algal haplotypes associating with more than one fungal taxon. Some associations suggested higher specificity, with five fungal taxa and algal haplotypes partnering only with one another. All taxa of the *Phylloporina* clade formed reciprocally exclusive symbiotic relationships with algal haplotypes from clade 26 (Fig. 6A, B), which exhibited only angular-rounded and irregularly arranged algal cells. Other *Porinaceae* clades formed varying partnerships with algal lineages 29 (Fig. 6C, D) and 33–36. Morphotypes recovered within these clades were mostly rectangular and arranged in radiate plates (Fig. 6C–H), with some exceptions (Fig. 7A–F). Furthermore, multiple algal haplotypes were occasionally recovered from a single thallus.

### Taxonomy

#### Porina cf. atriceps

Taxon classificationFungiPertusarialesPertusariaceae

(Vain.) Vain., Ann. Acad. Sci. Fenn., Ser. A 15(no. 6): 364. 1921.

B8B51FE8-A1F7-530C-A6D2-15CEC8956D10

Fig. 15A

##### Type.

DOMINICA • 1892, Elliot 517 (holotype: TUR).

##### Notes.

New record for Mayotte. *Porina atriceps* is a pantropical species known mainly from the Caribbean and Southeast Asian Islands (Lücking and Vězda 1998). The Mayotte material comprises three specimens: 21856B, 21856C, and 21856E. Because Vainio’s (1896) original mention of the species, as a variety of *P. epiphylla*, lacks a detailed description, a comparison was made using the material studied by Lücking and Vězda (1998). The ascospores in the Mayotte material are always 7-septate, measuring (23.5–)27–32.5(–36.5) × (2.5–)2.9–3.7(–4.1) µm (n = 97), which is consistent with the published measurements of 27–35 × 3–4.5 µm. A dark brownish spot, 0.1–0.3 mm wide, is visible on the perithecia. In some perithecia, it appears somewhat paler and broader than the previously reported 0.1–0.2 mm blackish–brown spot. The lens-shaped perithecia in the Mayotte specimens are also somewhat wider, measuring 0.3–0.55 mm (n = 38), compared with 0.2–0.4 mm reported in the literature. In the ITS and the mtSSU phylogenies, the specimens were recovered in the *Phylloporina* clade (Figs 2, 3). Specimens demonstrated high selectivity and were exclusively associated with algal haplotype 26:4 (Figs 4, 5). A single mtSSU sequence identified as *P. atriceps* (OM219174) from an unpublished Paleotropical study was included in the phylogeny but clustered with specimens of *P. karnatakensis* and *P. aff. lucida* 1 (Fig. 2). Given the marked inconsistency between its phylogenetic position and the published morphology of *P. atriceps*, this accession should be interpreted with caution. Overall, because *P. atriceps* was originally described from the Neotropics and no type-linked sequences are currently available, the Mayotte specimens cannot be confidently assigned to *P. atriceps* s. str. They are therefore retained as *P. cf. atriceps*.

##### Specimens examined.

MAYOTTE – **Choungui** • trail to Mount Choungui, 12°57'38.7"S, 45°08'08.4"E, alt. 365 m, dense humid forest, 29 Sep. 2017; Ertz 21856B (BR), 21856C (BR), 21856E (BR, MAO).

#### Porina cf. australiensis

Taxon classificationFungiPertusarialesPertusariaceae

(Lücking & Vězda) Lücking, Biblioth. Lichenol. 88: 413. 2004.

59D17AB4-38EF-574C-B57B-7B428BC542D9

Fig. 15B

##### Type.

AUSTRALIA • Queensland, Crediton State Forest, 840 m, rain forest, 6.1986, Streimann 37663a (holotype: UPS; isotype: CANB, Herb. R.Lücking).

##### Notes.

New record for Madagascar. *Porina australiensis*, originally found in lowland rainforests of northern Australia (Lücking and Vězda 1998), is represented here by a single specimen from southern Madagascar (25303H). The preserved morphological characters align with the original description by Lücking and Vězda (1998), although the specimen examined bears only a few perithecia and seems to be in a deteriorated state. The 7-septate ascospores measure (35–)36–38.5(–39) × (5.1–)5.2–6.2(–6.6) µm (n = 9), consistent with the previously reported dimensions of 30–38 × 5–6 µm. Furthermore, the ca. 0.6 mm (n = 2) lens-shaped perithecia with applanate tops align with the original description. In the ITS and the mtSSU phylogenies, the specimen was recovered within the *Phylloporina* clade and may represent the first sequenced material attributable to *P. australiensis* (Figs 2, 3). The examined specimen associates with algal haplotype 26:5 (Figs 4, 5). Its mtSSU sequence clusters with an accession identified as *P. mastoidella* (Nyl.) Müll.Arg. from an unpublished study. This is surprising since *P. mastoidella* is a corticolous species, whereas *P. australiensis* is foliicolous. However, the substrate of the specimen from the *P. mastoidella* GenBank accession is unknown. Although substrate shifts with limited genetic divergence have been demonstrated in *Porinaceae*, they appear to be relatively uncommon. The *P. mastoidella* accession should therefore be interpreted with considerable caution. In the absence of type-linked sequences, together with the degraded condition of the specimen, the available evidence is insufficient to confidently assign the material to *P. australiensis* s. str. It is therefore retained as *P. cf. australiensis*.

##### Specimens examined.

MADAGASCAR – **Toliara Prov**. • ca. 25 km Northwest of Fort-Dauphin (Tolagnaro), National Park of Andohahela, South part of parcel 1, ca. 5 km West of the village of Malio, 24°55'43.4"S, 46°44'37.2"E, alt. 360 m, dense humid forest at the bottom of a valley near a river bordered by large granite rocks, 27 Nov. 2019; Ertz 25303H (BR, TAN).

#### Porina cincta

Taxon classificationFungiPertusarialesPertusariaceae

Debaene & Ertz sp. nov.

F5D5D83D-CDEC-547F-BC6C-3C1BAC0E8EBB

865732

Fig. 8

##### Type.

MADAGASCAR • Toliara Province, ca. 26 km Northwest of Fort-Dauphin (Tolagnaro), National Park of Andohahela, South part of parcel 1, ca. 6.5 km West of the village of Malio, 24°56'24.1"S, 46°43'26.2"E, alt. 1000 m, dense humid forest, 01 Dec. 2019; Ertz 25409F (holotype: BR5030171982725; isotype: TAN).

##### Etymology.

The epithet refers to the perithecia, which are encircled by a ring of white hairs.

##### Diagnosis.

This species differs from *Porina dwesica* Brusse & C.H. Dickinson in its photobiont, which is irregularly arranged and angular-rounded rather than rectangular in radiate plates; in its involucrellum and excipulum, which show a K+ orange–red reaction rather than a K- reaction; and in its ascospores, which are slightly wider than those reported (5.2–6.5 µm in *P. cincta* vs. 4–5.2 µm in *P. dwesica*).

##### Description.

***Thallus*** epiphyllous; diffuse and dispersed, ca. 10–15 µm thick; smooth, matt; greyish green to pale brownish green, difficult to distinguish from the leaf surface. ***Photobiont*** angular-rounded, irregularly arranged; ca. (5–)6–9(–10) × 3–5(–6) µm (n = 9). ***Perithecia*** basally immersed in thallus, not well delimited from thallus; immature perithecia lens-shaped to conical, mature perithecia lens-shaped to hemispherical, rarely subglobose when less deeply immersed; smooth surface; ca. 0.3–0.5 mm diam. (n = 10), and ca. 140–200 µm high; pale to dark reddish brown; ring of white hairs surrounding the perithecium at its point of emergence from the thallus, hairs thick and soft, consisting of agglutinated hyphae, ca. 20–100 µm in length and ca. 10–20 µm in width; ostiole inconspicuous, apex slightly applanate. ***Crystallostratum*** not present. ***Excipulum*** ca. 15–35 µm thick; pale orange to red, K+ red. ***Involucrellum*** ca. 20–35 µm thick; pale orange to red, K+ red. ***Hamathecium*** hyaline; not inspersed; composed of thin, simple paraphyses ca. 1–2 μm diam., and ca. 75–100 µm long. ***Asci*** obclavate; 85–100 × 18–20 µm (n = 5); 8-spored; with apical ring structure. ***Ascospores*** 7-septate, broadly oblong to fusiform (30–)31.5–34.5(–36) × (5.2–)5.6–6.1(–6.5) µm, ratio L/B (5.2–)5.3–6(–6.4) (n = 52). ***Pycnidia*** not observed. ***Chemistry*** not tested for thallus; TLC not performed.

##### Distribution and ecology.

The species is known from only one locality in southern Madagascar. It inhabits the upper surface of leaves in rather dense, humid montane forests at moderate elevation (ca. 1000 m).

##### Notes.

**Figure 8.**
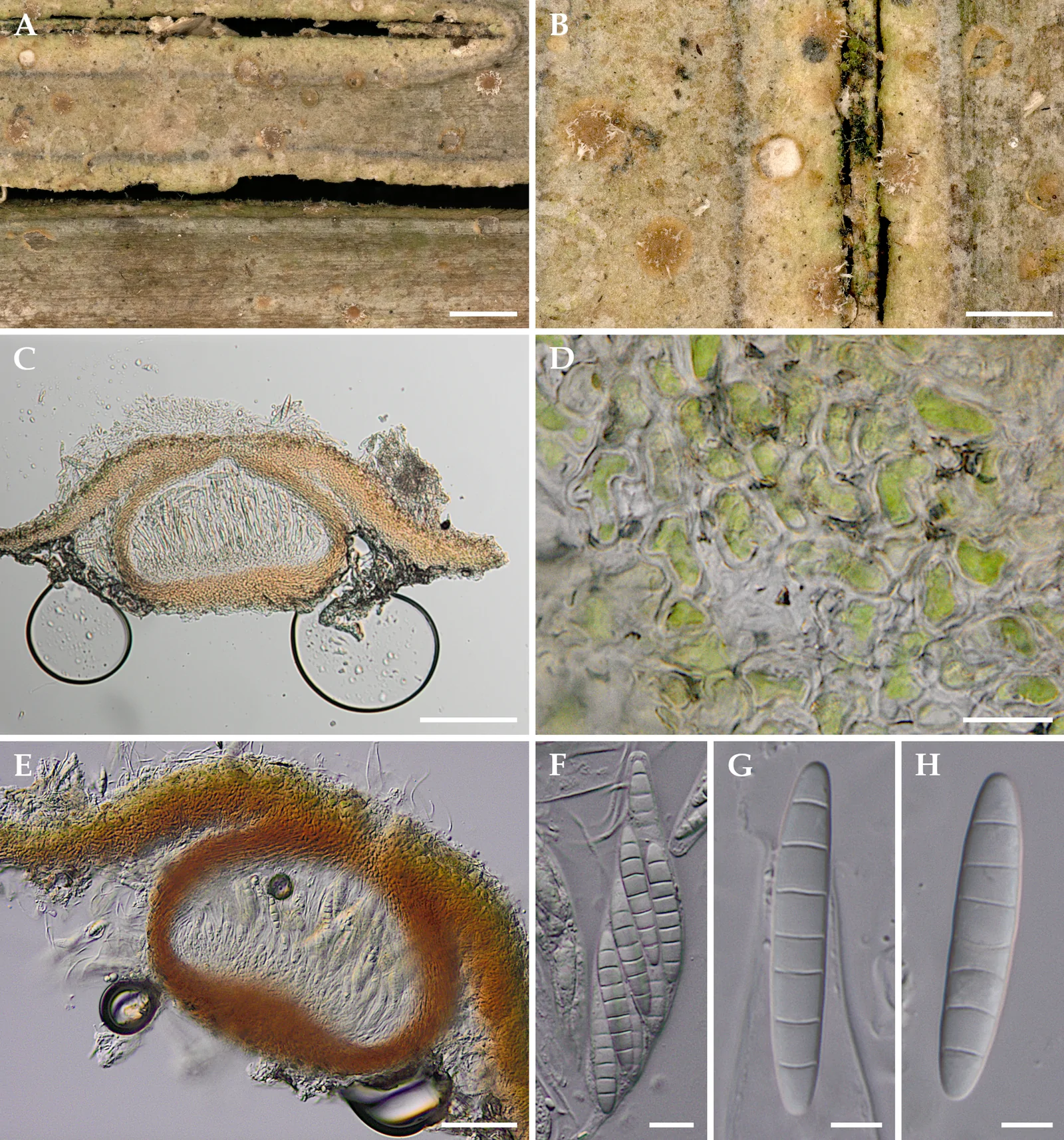
Diagnostic features of *Porina cincta* (25409F, Madagascar). **A** Diffuse and dispersed thallus, difficult to distinguish from the leaf surface, with perithecia **B** Close–up of perithecia basally immersed in thallus, lens-shaped to hemispherical, and ring of white hairs surrounding the perithecium at its point of emergence from the thallus **C** Perithecial cross-section in water, showing a pale orange to red involucrellum and excipulum **D** Photobiont in water, showing angular-rounded cells arranged irregularly **E** Perithecial cross-section in K, showing a K+ orange to red reaction of the excipulum and the involucrellum **F** Ascus in water, exhibiting eight ascospores and an apical ring **G**, **H** Ascospore in water. Scale bars: 1 mm (**A**); 0.5 mm (**B**); 100 µm (**C**); 10 µm (**D, F**); 50 µm (**E**); 5 µm (**G, H**).

Because the thallus of the single specimen was difficult to distinguish from the leaf surface (Fig. 8A), its width could not be estimated. Multiple solitary perithecia were found across the host leaf, suggesting diffuse colonisation without the formation of large confluent thalli. The host leaf was rather stiff and thick (possibly *Pandanus* sp.), unlike many of the other leaves bearing foliicolous lichens. *Porina cincta* was compared with the unsequenced *P. dwesica*, the morphologically most similar species, originally reported from South Africa (Transkei district, now Eastern Cape province) by Brusse and Dickinson (1991). Both species share similarly coloured perithecia and a ring of white hairs, although the fawn-coloured perithecia described for *P. dwesica* appear paler than those of *P. cincta*. Furthermore, photographs accompanying the original description show numerous soft white hairs extending from the base to the apex of the perithecia, whereas in *P. cincta*, the hairs are fewer and confined to the basal region (Fig. 8B). The most distinctive difference between the two species lies in the anatomy and chemistry of the perithecial inner walls. *Porina cincta* possesses a 20–35 µm thick, pale orange to red involucrellum (Fig. 8C) with a distinctly K+ orange to red reaction (Fig. 8E), whereas *P. dwesica* has a ca. 10 µm thick hyaline involucrellum that is K-. In addition, *P. dwesica* bears pycnidia and occasionally produces 9-septate ascospores, neither of which was observed in *P. cincta*. The two species also differ in photobiont morphology, with *P. dwesica* possessing rectangular cells arranged in radiate plates, whereas *P. cincta* has irregularly arranged angular-rounded photobiont cells (Fig. 8D). Although no *rbc*L sequence was recovered for this species, the photobiont morphology resembles that of *P. cf. leptosperma* (Fig. 7A, B), which is associated with the 33:3 algal haplotype, particularly in its relatively large intercellular spaces. In both the mtSSU and ITS phylogenies, *P. cincta* was recovered within the *Trichothelium* clade, despite possessing K+ orange to red perithecial walls that contrast with the predominantly black K- perithecia characterising most members of that lineage. The combination of a distinct phylogenetic position and diagnostic differences supports the recognition of *P. cincta* as a distinct species.

#### Porina complanata

Taxon classificationFungiPertusarialesPertusariaceae

Debaene & Ertz sp. nov.

C8F25E89-DFAD-5AD0-9CC8-5594594F8F7B

865733

Fig. 9

##### Type.

MADAGASCAR • Toliara Province, ca. 26 km Northwest of Fort-Dauphin (Tolagnaro), National Park of Andohahela, South part of parcel 1, ca. 6 km West of the village of Malio, 24°55'46.9"S, 46°43'55.9"E, alt. 515 m, dense humid forest, 28 Nov. 2019; Ertz 25321B (holotype: BR5030171983753; isotype: TAN).

##### Etymology.

The epithet refers to the flattened or immersed perithecia.

##### Diagnosis.

This species differs from *Porina australiensis* (Lücking & Vězda) Lücking in usually having completely applanate perithecia (vs. lens-shaped perithecia with applanate tops in *P. australiensis*); in having an unevenly rugose thallus (vs. smooth in *P. australiensis*); and in having narrower ascospores (3.4–4.7 µm in *P. complanata* vs. 5–6 µm in *P. australiensis*).

##### Description.

***Thallus*** epiphyllous; diffuse to continuous and marginally dispersed, ca. 3–15 mm wide, and ca. 30 µm thick; often a whitish translucent prothallus present between patches at the margins; unevenly rugose, incrusted with calcium oxalate crystals; matt to slightly glossy; pale greyish green to yellowish green. ***Photobiont*** angular-rounded, irregularly arranged; ca. (5–)6–13(–18) × (2–)3–5(–6) µm (n = 34). ***Perithecia*** immersed to superficial, not well delimited from thallus, usually completely applanate, occasionally becoming weakly lens-shaped; uneven surface, glabrous; ca. 0.5–0.9 mm diam. (n = 33), and ca. 50–100 µm high; covered by ca. 10 µm thick algiferous thallus layer; greenish-yellow to pale yellow, periostiolar area reddish to brown (ca. 0.05–0.2 mm), ostiolar depression sometimes present in mature perithecia with darker reddish-brown circle appearing around ostiole of ca. 0.1–0.2 mm diam. ***Crystallostratum*** ca. 15–30 µm thick. ***Excipulum*** ca. 15–20 µm thick; hyaline, K+ pale orange. ***Involucrellum*** ca. 30–50 µm thick; greyish brown, K+ orange to red. ***Hamathecium*** hyaline; not inspersed; composed of thin, simple paraphyses ca. 1–2 μm diam., and ca. 60–80 µm long. ***Asci*** narrowly fusiform; 90–120 × 9–13 µm (n = 9); 8-spored; with apical ring structure. ***Ascospores*** 7-septate, oblong to narrowly fusiform, (27.5–)30–35(–40.5) × (3.4–)3.7–4.3(–4.7) µm, ratio L/B (6.5–)7.3–8.9(–10.5) (n = 132), gelatinous sheath not or poorly visible. ***Pycnidia*** not observed. ***Chemistry*** not tested for thallus; TLC not performed.

##### Distribution and ecology.

The species is known from four different localities in Madagascar. It inhabits the surfaces of leaves in rather dense, humid forests and open mixed woodlands at low to moderate elevations (ca. 360–1177 m).

##### Notes.

**Figure 9.**
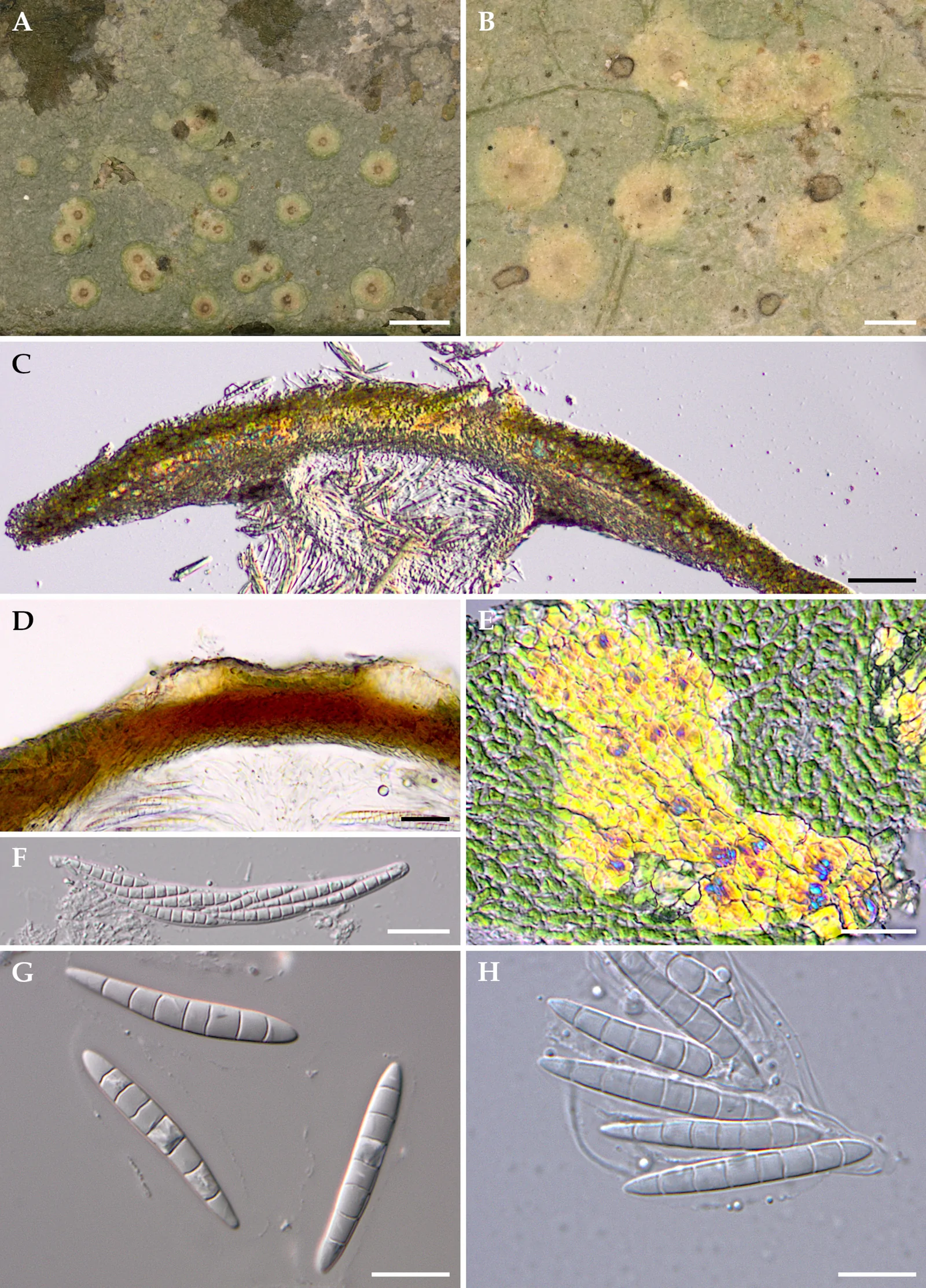
Diagnostic features of *Porina complanata* (Madagascar). **A** Continuous thallus with applanate perithecia and an ostiolar depression with darker red to brown circle around the ostiole (25321B) **B** Close–up of perithecia (25303C) **C** Perithecial cross-section in water, showing the algal layer on top and the crystallostratum (25322D) **D** Perithecial cross-section in K, showing a K+ orange to red reaction in the inner layers (25322D) **E** Photobiont in water, showing angular-rounded cells arranged irregularly, and calcium oxalate crystals incrusted in thallus (25321B) **F** Ascus in water, exhibiting eight ascospores and an apical ring (20270B) **G** Ascospores in water (25303C) **H** Ascospores in water (25321B). Scale bars: 1 mm (**A**); 0.5 mm (**B**); 50 µm (**C**); 25 µm (**D**); 20 µm (**E, F**); 10 µm (**G, H**).

*Porina complanata* most closely resembles *P. australiensis* in its similar photobiont morphotype and overlapping 7-septate ascospore length. It differs, however, in having completely applanate to immersed perithecia rather than lens-shaped perithecia with an applanate apex, an unevenly rugose thallus, narrower ascospores, and an apparent absence of pycnidia, which are frequently present in *P. australiensis* (Lücking and Vězda 1998). The species also resembles *P. subapplanata* Malcolm, Vězda, P.M. McCarthy & Kantvilas, as well as *P. pseudoapplanata* Lücking & M. Cáceres, in possessing applanate or immersed perithecia (Fig. 9A, B). Both species differ in having rectangular photobionts arranged in radiate plates, whereas *P. complanata* has angular-rounded photobionts arranged irregularly (Fig. 9E). *Porina subapplanata* is further distinguished by its occasionally white–pubescent ostiole, broader asci and ascospores, and the apparent absence of an ostiolar depression surrounded by a dark reddish-brown circle, according to the original description by Malcolm et al. (1999). *Porina pseudoapplanata* differs in having a smooth thallus, isidia, no crystals in the thallus or perithecia, a more prominent perithecial apex, shorter asci, slightly narrower ascospores, and perithecia that are never immersed, according to the original description by Lücking and Cáceres (1999). No confirmed sequences of *P. australiensis*, *P. subapplanata*, and *P. pseudoapplanata* are currently available for comparison. The single specimen identified as *P. australiensis* in this study does not cluster with the *P. complanata* sequences in either phylogeny (Figs 2, 3). *Porina complanata* lineage is represented by six sequenced specimens forming two subgroups within the *Phylloporina* clade. The relationship between these subgroups receives significant support values (BS = 79) in the ITS phylogeny but is unsupported in the mtSSU phylogeny (BS = 55). Both subgroups are nearly morphologically identical. Although the subgroup comprising specimens 20270I, 20270B, and 25303C tends to have more immersed perithecia, the other subgroup tends to have more flattened perithecia that protrude slightly from the thallus surface. Furthermore, this species was recovered in association with four different algal haplotypes (26:1, 26:2, 26:7, and 26:11), indicating substantial symbiotic flexibility. Additional data are needed to determine whether the observed genetic divergence supports recognition of more than one species.

##### Additional specimens examined.

MADAGASCAR – **Toliara Prov**. • ca. 26 km Northwest of Fort-Dauphin (Tolagnaro), National Park of Andohahela, South part of parcel 1, ca. 6 km West of the village of Malio, 24°55'46.9"S, 46°43'55.9"E, alt. 515 m, dense humid forest, 28 Nov. 2019; Ertz 25322D (BR, TAN). • ibid., ca. 5 km West of the village of Malio, 24°55'43.4"S, 46°44'37.2"E, alt. 360 m, dense and humid forest at the bottom of a valley near a river bordered by large granite boulders, 27 Nov. 2019; Ertz 25303C (BR, TAN). – **Diego Suarez Prov**. • Antsiranana, West of Sambava, Marojejy National Park, along trail from Camp Mantella to Camp Marojejia, rather dense humid evergreen lower montane forest, 14°26'07"S, 49°46'05"E, alt. ca. 650 m, 18 Oct. 2014; Ertz 20227D (BR, TAN). • ibid., Region: Diana, District: Diego II, Commune: Joffreville, Forontany: Morafeu, National Park of Montagne d’Ambre, Gîte des Roussettes, mixed open woodland, around house and picnic area, 12°31'37"S, 49°10'18"E, alt. 1068 m, 6 Oct. 2014, Ertz 20270I, 20270B (BR, TAN).

###### *Porina cf. epiphylla* 1 & 2 Fée, Essai Crypt. Exot. (Paris): 76. 1825 [1824].

Fig. 14A, B

**Type**. FRENCH GUIANA • Leprieur (lectotype: PC, designated by Santesson (1952: 234)).

**Notes**. *Porina epiphylla* is a common and widely distributed tropical species (Lücking 2008). It has been reported in Madagascar by Aptroot (2016) and in Mauritius by Ertz and Diederich (2022). *Porina cf. epiphylla* 1 (Fig. 14A) is represented by specimen 20274D from northern Madagascar, whereas *P. cf. epiphylla* 2 (Fig. 14B) is represented by specimen 24006A from Mauritius. Both specimens broadly match the published concept of *P. epiphylla* (Lücking and Vězda 1998; Lücking 2008), possessing a continuous, marginally dispersed, and minutely uneven thallus and lens-shaped to hemispherical perithecia with a slight reddish tinge. The reported 7-septate ascospores, measuring 26–33 × 3–4 µm, and perithecia, measuring 0.25–0.45 mm, are largely consistent with the examined specimens. In *P. cf. epiphylla* 1, ascospores measure (25.5–)27–32(–35.5) × (3.1–)3.4–4.4(–5.1) µm (n = 32) and perithecia 0.4–0.45 mm (n = 11), whereas in *P. cf. epiphylla* 2 the ascospores measure (26–)30–34(–36) × (4.2–)4.5–5.4(–6.5) µm (n = 45) and perithecia measure 0.4–0.45 mm (n = 10). Despite their overall similarity, each specimen departs slightly from the published concept of *P. epiphylla*. *Porina cf. epiphylla* 1 appears to possess a very thin, almost translucent thallus, a feature not observed in the examined specimens from the *Phylloporina* clade. Meanwhile, *P. cf. epiphylla* 2 has slightly longer and broader ascospores. In the ITS and mtSSU phylogenies, the sequences from the two specimens do not cluster together; instead, they form two distinct lineages within the *Phylloporina* clade (Figs 2, 3). *Porina cf. epiphylla* 1 is recovered in the Paleotropical *P. epiphylla-P. lucida* subclade, whereas *P. cf. epiphylla* 2 is recovered outside of this group. Neither mtSSU sequence clusters with the Neotropical accessions of *P. epiphylla*, the region where the species was described, suggesting that these specimens might represent taxa distinct from *P. epiphylla* s. str. Interestingly, two independently extracted neighbouring thallus fragments of *P. cf. epiphylla* 2 yielded different but closely related mtSSU sequences. Neither of the amplified products showed any clear sign of contamination. *Porina cf. epiphylla* 2 was associated exclusively with algal haplotype 26:9, whereas *P. cf. epiphylla* 1 was associated with two algal haplotypes (26:1 and 26:6) (Figs 4, 5). Overall, the available evidence indicates that the two specimens represent distinct Paleotropical lineages that broadly resemble the current morphological concept of *P. epiphylla* but cannot be confidently assigned to *P. epiphylla* s. str. in the absence of type-linked molecular data. They are therefore retained as *P. cf. epiphylla* 1 and *P. cf. epiphylla* 2.

**Specimens examined**. MADAGASCAR – **Diego Suarez Prov**. • Antsiranana, Region: Diana, District: Diego II, Commune: Joffreville, Forontany: Morafeu, National Park of Montagne d’Ambre, along trail from summit Olioly to small dam near camp Gîte des Roussettes, through moist montane forest, 12°32'34"S, 49°09'23"E, alt. 1242 m, 8 Oct. 2014, Ertz 20274D (BR, TAN). – MAURITIUS – **Rivière Noire District** • Black River Gorges National Park, along trail from Plaine Champagne towards Piton de la Petite Rivière Noire, 20°24'52"S (ca. 1000 m), 57°24'43"E (ca. 500 m), alt. 630–800 m, dense evergreen mountainous forest, 07 Sep. 2019; Ertz 24006A (BR).

#### Porina aff. epiphylla

Taxon classificationFungiPertusarialesPertusariaceae

Fée, Essai Crypt. Exot. (Paris): 76. 1825) [1824].

978CF117-BDF1-566E-B4E7-2D1A847FE53F

Fig. 14C

##### Type.

FRENCH GUIANA • Leprieur (lectotype: PC, designated by Santesson (1952: 234)).

##### Notes.

This taxon is represented by a single specimen (22131H). Morphologically, it most closely resembles *P. epiphylla* in possessing lens-shaped to hemispherical perithecia with a slight reddish tinge and similarly wide 7-septate ascospores. Its ascospores measure (29–)30–38(–42) × (2.4–)2.7–3.8(–4) µm (n = 10), and perithecia measure 0.3–0.45 mm (n = 5) in diameter. However, the specimen differs from the current concept of *P. epiphylla* in having longer ascospores that only partially overlap the reported range of 26–33 × 3–4 µm (Lücking and Vězda 1998; Lücking 2008). Furthermore, the examined specimen has a distinctly coarse, uneven thallus that is not an artefact of the leaf surface, which differs from the minutely uneven thallus described for *P. epiphylla*. In the mtSSU phylogeny, the specimen was recovered within the *Phylloporina* clade and more specifically within the Paleotropical *P. epiphylla*-*P. lucida* subclade (Fig. 3). Despite its morphological differences, it clusters with a *P. aff. lucida* 3 specimen (Fig. 14H) and differs from that taxon by very little mtSSU divergence. Nevertheless, the two taxa overlap only minimally morphologically. *Porina aff. lucida* 3 possesses considerably larger perithecia (0.5–0.7 mm; n = 19); a much darker reddish periostiolar region; a smooth and substantially larger thallus; and longer, narrower 7-septate ascospores, measuring (37–)39.5–43.5(–44) × 3–3.4(–3.5) µm (n = 10). Both taxa are associated with the algal haplotype 26:8 (Figs 4, 5). Overall, this specimen combines morphological affinity with *P. epiphylla* and phylogenetic affinity with the Paleotropical *P. epiphylla*-*P. lucida* subclade but differs sufficiently from the current concept of *P. epiphylla* to preclude a confident identification. Pending additional sampling and, ideally, type-linked sequences, it is therefore retained as *P. aff. epiphylla*.

##### Specimens examined.

MAYOTTE – **Sohoa** • Sohoa forest, 12°48'17.2"S, 45°06'47.6"E, alt. 150 m, mesophilic sloping forest, 05 Oct. 2017; Ertz 22131H (BR).

#### Porina cf. epiphylloides

Taxon classificationFungiPertusarialesPertusariaceae

Vězda, Folia geobot. phytotax. 10: 393. 1975.

EB4F0EAF-F1E5-58FF-9341-293F3BF5181A

Fig. 16A

##### Type.

TANZANIA • SW slope of Rwunge volcano; 1800–1900 m; Aug. 1972; Pócs 6762 (holotype: PRA).

##### Notes.

New record for Madagascar. *Porina epiphylloides* is mainly known from tropical East Africa and adjacent islands, where it occurs in submontane to montane rainforests (Lücking and Vězda 1998). This taxon is represented by three examined specimens: two from Madagascar (25302D and 25321E) and one from Mauritius (24305H). In the mtSSU phylogeny, the sequences cluster with *the P. epiphylloides* sequence reported by Ertz and Diederich (2022) within the Paleotropical subclade of the large *Porina* s. str. clade (Fig. 2). The sequences show little genetic divergence, suggesting that the specimens represent a single taxon. Despite this close phylogenetic affinity, the examined material does not fully match the measurements reported for the specimen by Ertz and Diederich (2022) or the description of *P. epiphylloides* by Lücking and Vězda (1998). This discrepancy mainly concerns ascospore length: the 7-septate ascospores measure (25–)26.5–31(–34) × (2.8–)3.1–3.5(–3.8) µm (n = 113), compared with 21–27 × 3 µm in the description. However, the perithecial morphology, thallus, and photobiont morphotype agree well with the description by Lücking and Vězda (1998). *Porina cf. epiphylloides* appears highly selective and is associated with a single algal clade and haplotype, 34:1 (Figs 4, 5). Overall, the relatively minor differences, together with the absence of type-linked sequences, make it difficult to confidently assign these specimens to *P. epiphylloides* s. str. The specimens are therefore retained as *P. cf. epiphylloides*.

##### Specimens examined.

MADAGASCAR – **Toliara Prov**. • ca. 25 km Northwest of Fort-Dauphin (Tolagnaro), National Park of Andohahela, South part of parcel 1, ca. 5 km West of the village of Malio, 24°55'43.4"S, 46°44'37.2"E, alt. 360 m, dense and humid forest at the bottom of a valley near a river bordered by large granite boulders, 27 Nov. 2019; Ertz 25302D (BR, TAN). • ibid., ca. 26 km Northwest of Fort-Dauphin (Tolagnaro), National Park of Andohahela, South part of parcel 1, ca. 6 km West of the village of Malio, 24°55'46.9"S, 46°43'55.9"E, alt. 515 m, dense humid forest, 28 Nov. 2019; Ertz 25321E (BR, TAN). – MAURITIUS – **Rivière Noire District** • Black River Gorges National Park, 5 km North-West of Pétrin, Brise Fer Forest, 20°22'40"S (ca. 200 m), 57°26'25"E (ca. 200 m), 585 m, 10 Sep. 2019; Ertz 24305H and Diederich (BR).

###### *Porina cf. karnatakensis* 1 & 2 Makhija, Adaw. & Patw., J. Econ. Taxon. Bot. 18(3): 538. 1995 [1994].

Fig. 15C, D

**Type**. INDIA • Karnataka: Londha to Yellapur road, Patwardhan and Prabhu 74.3642 (holotype: AMH).

**Notes**. New record for Madagascar and Mayotte. *Porina karnatakensis* is a widely distributed pantropical species found in the shaded rainforest understories to dry evergreen forests (Lücking 2008). *Porina cf. karnatakensis* 1 (Fig. 15C) is represented by two specimens from Mayotte (22135B and 22135E), whereas *P. cf. karnatakensis* 2 (Fig. 15D) comprises three specimens from northern and southern Madagascar (20276J, 25303G, and 25374C). The specimens closely match the current concept of *P. karnatakensis*, a species also described as *Porina atropunctata* Lücking & Vězda, which was subsequently synonymised with *P. karnatakensis* by Lücking and Kalb (2002). However, the original description of *P. karnatakensis* by Makhija et al. (1994) differs substantially from that of *P. atropunctata* by Lücking and Vězda (1998). In the original description, *P. karnatakensis* is described as having 7- to 9-septate ascospores, measuring 33–63 × 4 µm, whereas *P. atropunctata* has 7-septate ascospores, measuring (20–)25–32(–35) × 3–5 μm. Therefore, the description of *P. karnatakensis* in Lücking (2008), likely based on *P. atropunctata* material, is used here for comparison. Examined specimens possess 7-septate ascospores consistent with that description. In *P. cf. karnatakensis* 1, the ascospores measure (23–)26–34(–38) × (2.5–)2.8–3.7(–4.2) µm (n = 32), whereas those of *P. cf. karnatakensis* 2 measure (24.5–)26.5–30.5(–34) × (3–)3.3–3.9(–4.2) µm (n = 73), both agreeing well with the reported dimensions of (20–)25–35 × 3–5 µm. The wart-shaped perithecia, bearing a black-brown periostiolar dot that typically fades with age, likewise correspond to the published description and remain narrower than those of *P. atriceps*. Perithecia measure 0.4–0.55 mm (n = 28) in *P. cf. karnatakensis* 1 and 0.25–0.45 mm (n = 51) in *P. cf. karnatakensis* 2, consistent with the reported range of 0.3–0.5 mm. In the mtSSU phylogeny, sequences from both taxa do not cluster with the Neotropical *P. karnatakensis* subclade (Fig. 2). Nor do the two *P. cf. karnatakensis* taxa cluster together in either of the recovered ITS and mtSSU phylogenies (Figs 2, 3). Furthermore, in the absence of type-linked sequences, it remains uncertain if any of the recovered lineages might correspond to *P. karnatakensis* s. str. Several additional mtSSU sequences identified as *P. karnatakensis* from an unpublished Paleotropical study fall outside these lineages, clustering instead with *P. aff. lucida* 1 despite being inconsistent with the morphology currently attributed to *P. karnatakensis*. These identifications should therefore be interpreted cautiously. The mtSSU sequences of *P. cf. karnatakensis* 2 cluster with a specimen identified as *P. cf. epiphylla* by Ertz and Diederich (2022), which was likewise described as possessing a dark periostiolar region. The *P. cf. karnatakensis* 2 taxon additionally forms a supported group with *P. cf. atriceps* identified within this study, which is not surprising given their rough external similarities. Regarding their photobiont, *P. cf. karnatakensis* 1 is more selective and associates exclusively with algal haplotype 26:11, whereas *P. cf. karnatakensis* 2 is more flexible and associates with three algal haplotypes (26:1, 26:2, and 26:3) (Figs 4, 5). Overall, the uncertain application of the name *P. karnatakensis*, the absence of type-linked sequences, and the recovery of multiple distinct lineages prevent a confident assignment of the taxa to *P. karnatakensis* s. str. They are therefore retained as *P. cf. karnatakensis* 1 and *P. cf. karnatakensis* 2.

**Specimens examined**. MADAGASCAR – **Diego Suarez Prov**. • Antsiranana, West of Sambava, Marojejy National Park, Camp Mantella area, trail to Cascade de Humbert, through rather dense humid evergreen lower montane forest, stream bank at waterfall of large exposed bedrock area, 14°25'58"S, 49°46'22"E, alt. ca. 480 m, 18 Oct. 2014; Ertz 20276J (BR, TAN). – **Toliara Prov**. • ca. 25 km Northwest of Fort-Dauphin (Tolagnaro), National Park of Andohahela, South part of parcel 1, ca. 5 km West of the village of Malio, 24°56'07.0"S, 46°44'08.5"E, alt. 680 m, dense humid forest, 29 Nov. 2019, Ertz 25374C (BR, TAN). • ibid., 24°55'43.4"S, 46°44'37.2"E, alt. 360 m, dense and humid forest at the bottom of a valley near a river bordered by large granite boulders, 27 Nov. 2019, Ertz 25303G (BR, TAN). – MAYOTTE – **Sohoa** • Sohoa forest, 12°48'17.2"S, 45°06'47.6"E, alt. 150 m, mesophilic sloping forest, 05 Oct. 2017; Ertz 22135B (BR), 22135E (BR, MAO).

#### Porina cf. leptosperma

Taxon classificationFungiPertusarialesPertusariaceae

Müll. Arg., Flora, Regensburg 66(21): 333. 1883.

5E131464-7B12-53F9-964B-53C298227A9A

Fig. 18E, F

##### Type.

BRAZIL • Apiahy, J.I.Puiggari 1090 (holotype: G).

##### Notes.

New record for Madagascar. *Porina leptosperma* is a common but inconspicuous pantropical species of rainforest understories (Lücking 2008). The taxon is represented by a single specimen, 20274C, from northern Madagascar. The specimen is broadly consistent with the published descriptions of *P. leptosperma* (Lücking 2008; McCarthy 2013). The 3-septate ascospores, measuring (18.5–)21–26(–32) × (4.2–)4.9–5.9(–6.4) µm (n = 85), slightly exceed the previously published range of 14–24.5 × 3–4.5 µm. The thallus appears larger than previously described (Fig. 18E) and more closely resembles the continuous, irregular thallus described by Lücking (2008) than the smaller, irregular patches described by McCarthy (2013). The subglobose, orange perithecia measure 0.15–0.2 mm (n = 17) in diameter, matching the previously reported morphology and size of 0.13–0.26 mm. The taxon was recovered within the *Porina fusca* clade in both mtSSU and ITS phylogenies (Figs 2, 3) and may represent the first sequenced specimen attributable to *P. leptosperma*. The specimen was associated with photobiont haplotype 33:3 (Figs 4, 5), the only member of algal clade 33 characterised by angular-rounded, irregularly arranged cells. Overall, the specimen agrees closely with current morphological concepts of *P. leptosperma*; its larger thallus and broader ascospores, together with the absence of reliable type-linked sequences, preclude a confident assignment to *P. leptosperma* s. str. It is therefore retained as *P. cf. leptosperma*.

##### Specimens examined.

MADAGASCAR – **Diego Suarez Prov**. • Antsiranana, Region: Diana, District: Diego II, Commune: Joffreville, Forontany: Morafeu, National Park of Montagne d’Ambre, along trail from summit Olioly to small dam near camp Gîte des Roussettes, through moist montane forest, 12°32'34"S, 49°09'23"E, alt. 1242 m, 8 Oct. 2014; Ertz 20274C (BR, TAN).

#### Porina limbulata

Taxon classificationFungiPertusarialesPertusariaceae

(Kremp.) Vain., Ann. Acad. Sci. Fenn., Ser. A 15(no. 6): 363. 1921.

8A843917-1633-5A90-9782-A3BE110B6294

Fig. 18A

##### Type.

BORNEO • O.Beccari 225a (holotype: M).

##### Notes.

New record for Madagascar. *Porina limbulata* is a widely distributed tropical species common in rainforests and ecologically similar to *P. rufula* (Lücking 2008). This taxon is represented by two specimens from northern Madagascar (20271G and 20271I), both of which agree well with the published descriptions of *P. limbulata* by Lücking (1996, 2008) and McCarthy (2013). The 3-septate ascospores measure (18–)20–23(–25.5) × (3.7–)3.9–4.4(–4.6) µm (n = 57), corresponding most closely to the range of 18–28 × 2.5–4.5 µm reported by McCarthy (2013). The lens-shaped, dark red-brown perithecia measure 0.2–0.3 mm (n = 7) and match the reported morphology and size of 0.2–0.4 mm. Older perithecia additionally develop dark rings, as described by McCarthy (2013). In the mtSSU phylogeny, the specimens are recovered within a paraphyletic *P. limbulata* lineage within the *Phragmopeltheca* clade together with other Neotropical accessions, with *P. rubentior* nested within this group (Fig. 2). The taxon is associated with photobiont haplotype 33:1 (Figs 4, 5), which corresponds to the morphotype with rectangular cells arranged in radiate plates. Overall, the close agreement with the published morphology and phylogenetic placement supports assigning these specimens to *P. limbulata* s. str. under the current species concept, although the apparent paraphyly of the species suggests that its circumscription requires further investigation.

##### Specimens examined.

MADAGASCAR – **Diego Suarez Prov**. • Antsiranana, Region: Diana, District: Diego II, Commune: Joffreville, Forontany: Morafeu, National Park of Montagne d’Ambre, along trail from Gîte des Roussettes, across stream at small dam to dirt road, moist but not wet lower montane forest, with tall trees and tree ferns, 12°31'55"S, 49°10'33"E, alt. 1119 m, 7 Oct. 2014; Ertz 20271G, 20271I (BR, TAN).

###### *Porina cf. lucida* 1 & 2 R. Sant., Symb. bot. upsal. 12(no. 1): 240. 1952.

Fig. 14D, E

**Type**. INDONESIA • Java, Preanger, Tjibodas, Jul. 1898, Nyman 6B (holotype: UPS).

**Notes**. New record for Mayotte. *Porina lucida* is a common and widely distributed tropical species (Lücking 2008). It has previously been reported from Madagascar by Aptroot (2016). *P. cf. lucida* 1 (Fig. 14D) comprises specimens 20270H, 20272C, and 20272F from northern Madagascar, whereas *P. cf. lucida* 2 (Fig. 14E) is represented by specimen 21938B from Mayotte. Both taxa broadly conform to Santesson’s (1952) original description of *P. lucida*. Ascospores are 7-septate, measuring (31–)34–40(–44.5) × (4–)4.5–5.1(–5.3) µm (n = 24) in *P. cf. lucida* 1 and (36–)38–43(–45) × (4–)4.5–5.1(–5.3) µm (n = 57) in *P. cf. lucida* 2, broadly consistent with the originally reported dimensions of 35–45 × 4–5 µm (Santesson 1952). However, a minority (ca. 10%) of *P. cf. lucida* 2 ascospores are 8-septate. Both taxa possess lens-shaped to hemispherical perithecia with a spreading base, a pale yellowish tinge and an occasionally darker, applanate apex. Although the observed perithecia tend to be more hemispherical than lens-shaped, and those of *P. cf. lucida* 1 are slightly larger (0.5–0.7 mm; n = 10) than the originally reported 0.4–0.6 mm. In the ITS and the mtSSU phylogenies, the two taxa are recovered as distinct lineages within the Paleotropical *P. epiphylla-P. lucida* subclade of the *Phylloporina* clade (Figs 2, 3). The mtSSU phylogeny further shows that neither lineage clusters with the available Neotropical accessions identified as *P. lucida*, suggesting that these Neotropical accessions may represent distinct lineages. *Porina cf. lucida* 2 was associated exclusively with algal haplotype 26:1, *Porina cf. lucida* 1 was associated with two algal haplotypes (26:1 and 26:3) (Figs 4, 5). Overall, because the type of *P. lucida* originates from Java and no type-linked sequences are available, the identity of the Paleotropical lineage remains uncertain. The recovered taxa are therefore retained as *P. cf. lucida* 1 and *P. cf. lucida* 2.

**Specimens examined**. MADAGASCAR – **Diego Suarez Prov**. • Antsiranana, Region: Diana, District: Diego II, Commune: Joffreville, Forontany: Morafeu, National Park of Montagne d’Ambre, along trail from Gîte des Roussettes, across stream at small dam to dirt road and trail down to Lake Mahasarika, moist but not wet lower montane forest, with tall trees and tree ferns, 12°32'03"S, 49°10'28"E, alt. 1177 m, 7 Oct. 2014; Ertz 20272C, 20272F (BR, TAN). • ibid., Gîte des Roussettes, mixed open woodland, around house and picnic area, 12°31'37"S, 49°10'18"E, alt. 1068 m, 6 Oct. 2014; Ertz 20270H (BR, TAN). – MAYOTTE – **South of Tsararano** • trail to Mount Bénara, 12°52'45.2"S, 45°09'39.0"E, alt. 575 m, dense humid hillside forest, 01 Oct. 2017; Ertz 21938B (BR).

###### *Porina aff. lucida* 1, 2, & 3 R. Sant., Symb. bot. upsal. 12(no. 1): 240. 1952.

Fig. 14F–H

**Type**. INDONESIA • Java, Preanger, Tjibodas, Jul. 1898, Nyman 6B (holotype: UPS).

**Notes**. *Porina aff. lucida* 1 (Fig. 14F) is represented by specimens from Mauritius and northern and southern Madagascar (20271C, 23539F, 23539H, 25303F, 25375F, and 25411A), whereas *P. aff. lucida* 2 (Fig. 14G) and 3 (Fig. 14H) are both represented by a single specimen from northern Madagascar (20280H and 20276F, respectively). All three taxa have a morphology similar to that of *P. lucida*, as originally reported by Santesson (1952). Their 7-septate ascospore measure (31.5–)33–38(–41.5) × (3.3–)3.9–5.1(–5.3) µm (n = 63) in *P. aff. lucida* 1, (37–)40–48(–51) × (3.6–)3.9–4.7(–5.3) µm (n = 19) in *P. aff. lucida* 2, and (37–)39.5–43.5(–44) × 3–3.4(–3.5) µm (n = 10) in *P. aff. lucida* 3. Ascospore dimensions overlap only partially with the originally published range for *P. lucida* of 35–45 × 4–5 µm (Santesson 1952). The three taxa also differ in perithecial morphology. *Porina aff. lucida* 1 has hemispherical to conical perithecia (0.4–0.8 mm; n = 45) with a pale yellow to orange tinge, a variable spreading base, and a slightly convex to distinctly applanate, darker apex (ca. 0.1–0.35 mm wide). *Porina aff. lucida* 2 has conical perithecia (0.75–0.95 mm; n = 4) with a pale red to brown tinge, a strongly spreading base, and a distinctly applanate, darker apex (ca. 0.35–0.4 mm wide). *Porina aff. lucida* 3 has lens-shaped to hemispherical perithecia (0.5–0.7 mm; n = 19) with an orange to brown tinge, a slightly spreading base, and a convex, darker apex (ca. 0.15–0.25 mm wide). Altogether, the three taxa show only partly overlapping perithecial morphologies and generally larger perithecia than the reported lens-shaped perithecia (0.4–0.6 mm) with a pale yellowish tinge, a spreading base and a darker, sometimes applanate apex. In the ITS and mtSSU phylogenies, all three taxa are recovered within the Paleotropical *P. epiphylla*-*P. lucida* subclade of the *Phylloporina* clade but form three distinct lineages (Figs 2, 3). *Porina aff. lucida* 1 clusters with unpublished Paleotropical mtSSU accessions identified as *P. karnatakensis* and *P. atriceps*. However, those accessions are morphologically inconsistent with the published concepts of either species and are therefore interpreted with caution. A higher level of symbiont flexibility was observed in *P. aff. lucida* 1 and 2, with the first associating with four algal haplotypes (26:1, 26:2, 26:7, and 26:10) and the second with two haplotypes (26:1 and 26:7) from the same thallus (Figs 4, 5). By contrast, only one haplotype (26:8) was found on the single specimen representing *P. aff. lucida* 3. Overall, the combined morphological and molecular evidence supports the recognition of three distinct lineages showing affinity to *P. lucida*, but none can presently be confidently referred to *P. lucida* s. str. They are therefore retained as *P. aff. lucida* 1, *P. aff. lucida* 2, and *P. aff. lucida* 3.

**Specimens examined**. MADAGASCAR – **Diego Suarez Prov**. • Antsiranana, Region: Diana, District: Diego II, Commune: Joffreville, Forontany: Morafeu, National Park of Montagne d’Ambre, along trail from Gîte des Roussettes, across stream at small dam to dirt road, moist but not wet lower montane forest, with tall trees and tree ferns, 12°31'55"S, 49°10'33"E, alt. 1119 m, 7 Oct. 2014; Ertz 20271C (BR, TAN). • ibid., West of Sambava, Marojejy National Park, Camp Mantella area, trail to Cascade de Humbert, through rather dense humid evergreen lower montane forest, stream bank at waterfall of large exposed bedrock area, 14°25'58"S, 49°46'22"E, alt. ca. 480 m, 18 Oct. 2014; Ertz 20276F (BR, TAN). • ibid., along trail from Camp Marojejia down to Camp Mantella, rather dense humid evergreen lower montane forest, 14°26'07"S, 49°46'05"E, alt. ca. 650 m, 23 Oct. 2014; Ertz 20280H (BR, TAN). – **Toliara Prov**. • ca. 25 km Northwest of Fort-Dauphin (Tolagnaro), National Park of Andohahela, South part of parcel 1, ca. 5 km West of the village of Malio, 24°55'43.4"S, 46°44'37.2"E, alt. 360 m, dense humid forest at the bottom of a valley near a river bordered by large granite rocks, 27 Nov. 2019; Ertz 25303F (BR, TAN). • ibid., 24°56'07.0"S, 46°44'08.5"E, alt. 680 m, dense humid forest, 29 Nov. 2019; Ertz 25375F (BR, TAN). • ibid., ca. 26 km Northwest of Fort-Dauphin (Tolagnaro), National Park of Andohahela, South part of parcel 1, ca. 6.5 km West of the village of Malio, 24°56'24.1"S, 46°43'26.2"E, alt. 1000 m, dense humid forest, 01 Dec. 2019; Ertz 25411A (BR, TAN). – MAURITIUS – **Savanne District** • Black River Gorges National Park, along the trail to Mt Cocotte, 20°26'30"S (ca. 200 m), 57°28'16"E (ca. 500 m), alt. 720–750 m, 01 Sep. 2019; Ertz 23539F, 23539H and Diederich (BR).

#### Porina madagascariensis

Taxon classificationFungiPertusarialesPertusariaceae

Debaene & Ertz sp. nov.

BE12C183-9471-5C55-9159-7D8C7B2791CE

865735

Fig. 10

##### Type.

MADAGASCAR • Diego Suarez Province, Antsiranana, West of Sambava, Marojejy National Park, along trail from Camp Simpona down to Camp Marojejia, rather dense humid montane forest, 14°26'20"S, 49°45'08"E, alt. ca. 1200 m, 23 Oct. 2014; Ertz 20279O (holotype: BR5030171998917; isotype: TAN).

##### Etymology.

The epithet refers to the occurrence of the only known specimen in Madagascar.

##### Diagnosis.

This species differs from *Porina bellendenica* Müll.Arg. in its foliicolous habit (vs. corticolous and saxicolous in *P. bellendenica*); the absence of a dark-brown prothallus; broader perithecia (0.7–0.9 mm in diam. in *P. madagascariensis* vs. 0.35–0.6 mm in diam. in *P. bellendenica*); and a paler periostiolar area (red to brown in *P. madagascariensis* vs. dark-brown to black in *P. bellendenica*).

##### Description.

***Thallus*** epiphyllous; continuous and marginally dispersed, large, ca. 20–30 mm wide, and ca. 30 µm thick; surface smooth to slightly uneven, encrusted with calcium oxalate crystals; slightly glossy; pale yellowish green. ***Photobiont*** angular-rounded, irregularly arranged; ca. (4–)6–13(–15) × 2–4(–5) µm (n = 33). ***Perithecia*** superficial, not well delimited from thallus due to strongly spreading base, immature perithecia lens-shaped, mature perithecia lens-shaped to subhemispherical; smooth to slightly irregular surface, glabrous; ca. 0.7–0.9 mm diam. (n = 13), and ca. 200–250 µm high; covered by ca. 10–20 µm thick algiferous thallus layer; concolorous to slightly more yellowish than thallus; periostiolar area red to brown, getting darker and broader with age (ca. 0.2–0.3 mm), apex applanate with ostiole region sometimes slightly depressed. ***Crystallostratum*** ca. 10–20 µm thick. ***Excipulum*** ca. 15–20 µm thick; hyaline to yellow, K+ pale yellow to orange. ***Involucrellum*** ca. 30–45 µm thick; yellow, K+ orange to red. ***Hamathecium*** hyaline; not inspersed; composed of thin, simple paraphyses ca. 1–1.5 μm diam., and ca. 70–120 µm long. ***Asci*** fusiform; 110–135 × 13–15 µm (n = 6); 8-spored; with apical ring structure. ***Ascospores*** (9–)10–12(–14)-septate, bacillar to narrowly oblong (45.5–)48–55(–64) × (3.5–)3.9–4.4(–4.7) µm, ratio L/B (10.4–)11.2–13.8(–18.3) (n = 80); gelatinous sheath not or poorly visible. ***Pycnidia*** not observed. ***Chemistry*** not tested for thallus; TLC not performed.

##### Distribution and ecology.

The species is known from only one locality in northern Madagascar. It inhabits leaf surfaces in rather dense, humid montane forests at moderate elevation (ca. 1200 m).

##### Notes.

**Figure 10.**
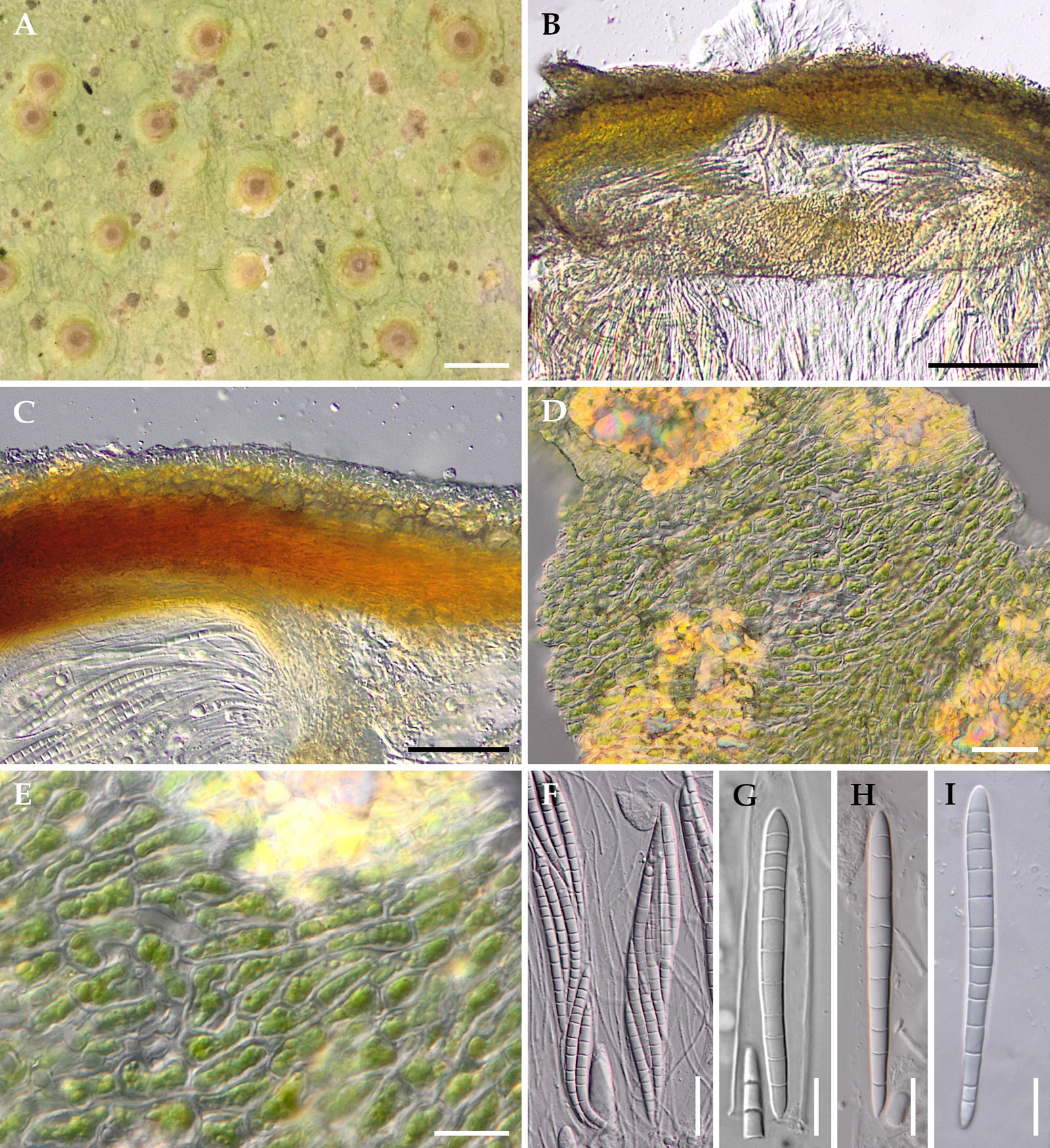
Diagnostic features of *Porina madagascariensis* (20279O, Madagascar). **A** Continuous thallus with lens-shaped perithecia with strongly spreading base **B** Perithecial cross-section in water, showing the algal layer on top, the thin crystallostratum, and the yellowish involucrellum and excipulum **C** Perithecial cross-section in K, showing a K+ orange to red reaction in the inner layers **D** Thallus section in water, showing angular-rounded algal cells arranged irregularly, and incrusted calcium oxalate crystals **E** Photobiont in water, showing angular-rounded cells arranged irregularly **F** Ascus in water, exhibiting eight ascospores and an apical ring **G** 11-septate ascospore in water **H** 10-septate ascospore in water **I** Bacillar to narrowly oblong 14-septate ascospore in water. Scale bars: 1 mm (**A**); 100 µm (**B**); 50 µm (**C**); 20 µm (**D, F**); 10 µm (**E, G–I**).

In the ITS and mtSSU phylogenies, *P. madagascariensis* forms an isolated lineage within the *Phylloporina* clade and is not closely related to any other sampled accession (Figs 2, 3). Distinct brown dots are visible on the specimen’s thallus (Fig. 10A), but they occur across the entire leaf surface and are therefore not associated with the species itself. *Porina madagascariensis* most closely resembles *P. bellendenica* in its ascospore morphology, with (9–)10–12(–14)-septate ascospores measuring (45.5–)48–55(–64) × (3.5–)3.9–4.4(–4.7) µm, closely matching the (9–)11–13(–15)-septate ascospores (32–56 × 3–5 µm) reported for *P. bellendenica* by McCarthy (2013). It differs, however, in being foliicolous rather than corticolous or saxicolous; in having broader, lens-shaped to subhemispherical perithecia with a red to brown periostiolar area instead of hemispherical perithecia with a dark-brown to black periostiolar area; in the absence of a dark-brown prothallus; and in the apparent absence of pycnidia. Moreover, the currently available mtSSU accessions for *P. bellendenica* are recovered in the *Phyllophiale* clade (See Suppl. material 3), whereas *P. madagascariensis* belongs to the *Phylloporina* clade. *Porina madagascariensis* is additionally one of the few foliicolous members of the *Phylloporina* clade that deviate from the predominantly 7-septate ascospore type. Similar increases in ascospore septation have previously been reported in several *Porinaceae* species (Lücking 2008: 337), e.g. *Porina virescens* (Kremp.) Müll.Arg., which includes a multi-septate form with 7–11(–15)-septate ascospores (Lücking and Vězda 1998; Lücking 2004). The species was recovered in association with algal haplotype 26:2 (Fig. 10D, E), which is shared with several other *Phylloporina*-clade taxa, such as *P. complanata* (Figs 4, 5). The combination of a distinct phylogenetic position and diagnostic differences supports the recognition of *P. madagascariensis* as a distinct species.

#### Porina mayottensis

Taxon classificationFungiPertusarialesPertusariaceae

Debaene & Ertz sp. nov.

526198E6-A462-52FD-A2BB-BDE8B9153B0F

865737

Fig. 11

##### Type.

MAYOTTE • South of Tsararano, Summit of Mount Bépilipili, 12°52'52.2"S, 45°10'09.2"E, alt. 659 m, humid mountainous forest, 03 Oct. 2017; Ertz 22081A (holotype: MAO; isotype: BR5030171995824).

##### Etymology.

The epithet refers to the apparent restriction of this species to Mayotte.

##### Diagnosis.

This species differs from *Porina mazosioides* Lücking & Vězda in having less protruding perithecia (lens-shaped to hemispherical in *P. mayottensis* vs. hemispherical to wart-shaped in *P. mazosioides*); the absence of distinct verrucae on the thallus; slightly broader ascospores (5–8 µm in *P. mayottensis* vs. 5–6 µm in *P. mazosioides*); and the presence of both angular-rounded and rectangular photobiont cells, often occurring on the same thallus.

##### Description.

***Thallus*** epiphyllous; small and dispersed or sometimes confluent, ca. 0.5–10 mm wide, and ca. 20 µm thick; sometimes with a whitish translucent prothallus (ca. 0.05 mm wide); finely rugose, encrusted with calcium oxalate crystals; matt to slightly glossy; greyish green to yellowish green. ***Photobiont*** rectangular cells, forming radiate plates and/or angular-rounded cells, irregularly arranged; ca. 10–16(–21) × (2–)3–4 µm (n = 22) when rectangular and (5–)8–15(–19) × 3–5(–8) µm (n = 66) when angular-rounded. ***Perithecia*** superficial, moderately delimited from thallus; immature perithecia lens-shaped and mature perithecia lens-shaped to hemispherical; surface smooth to rough, glabrous; ca. 0.3–0.6 mm diam. (n = 30), and ca. 120–140 µm high; covered by ca. 10–20 µm thick algiferous thallus layer; pale yellow to orange, periostiolar area slightly darker; apex convex to slightly applanate, ostiole inconspicuous. ***Crystallostratum*** ca. 10–40 µm thick. ***Excipulum*** ca. 10–15 µm thick; hyaline to pale yellow, K+ yellow to pale orange. ***Involucrellum*** ca. 10–15 µm thick; hyaline to pale yellow, K+ pale to bright orange. ***Hamathecium*** hyaline; not inspersed; composed of thin, simple paraphyses ca. 1–2 μm diam., and ca. 70–100 µm long. ***Asci*** fusiform to obclavate; 95–125 × 19–35 µm (n = 14); 8-spored; with apical ring structure. ***Ascospores*** 7-septate, oblong to narrowly fusiform (36–)42.5–48.5(–53) × (5–)5.8–7.1(–8) µm, ratio L/B (6.4–)7.2–8.6(–9.8) (n = 143), gelatinous sheath not or poorly visible and thin (ca. 1 µm). ***Pycnidia*** not observed. ***Chemistry*** not tested for thallus; TLC not performed.

##### Distribution and ecology.

The species is known from six different localities in Mayotte. It inhabits the surfaces of leaves in rather dense, humid forests at low to moderate elevations (ca. 370–660 m).

##### Notes.

**Figure 11.**
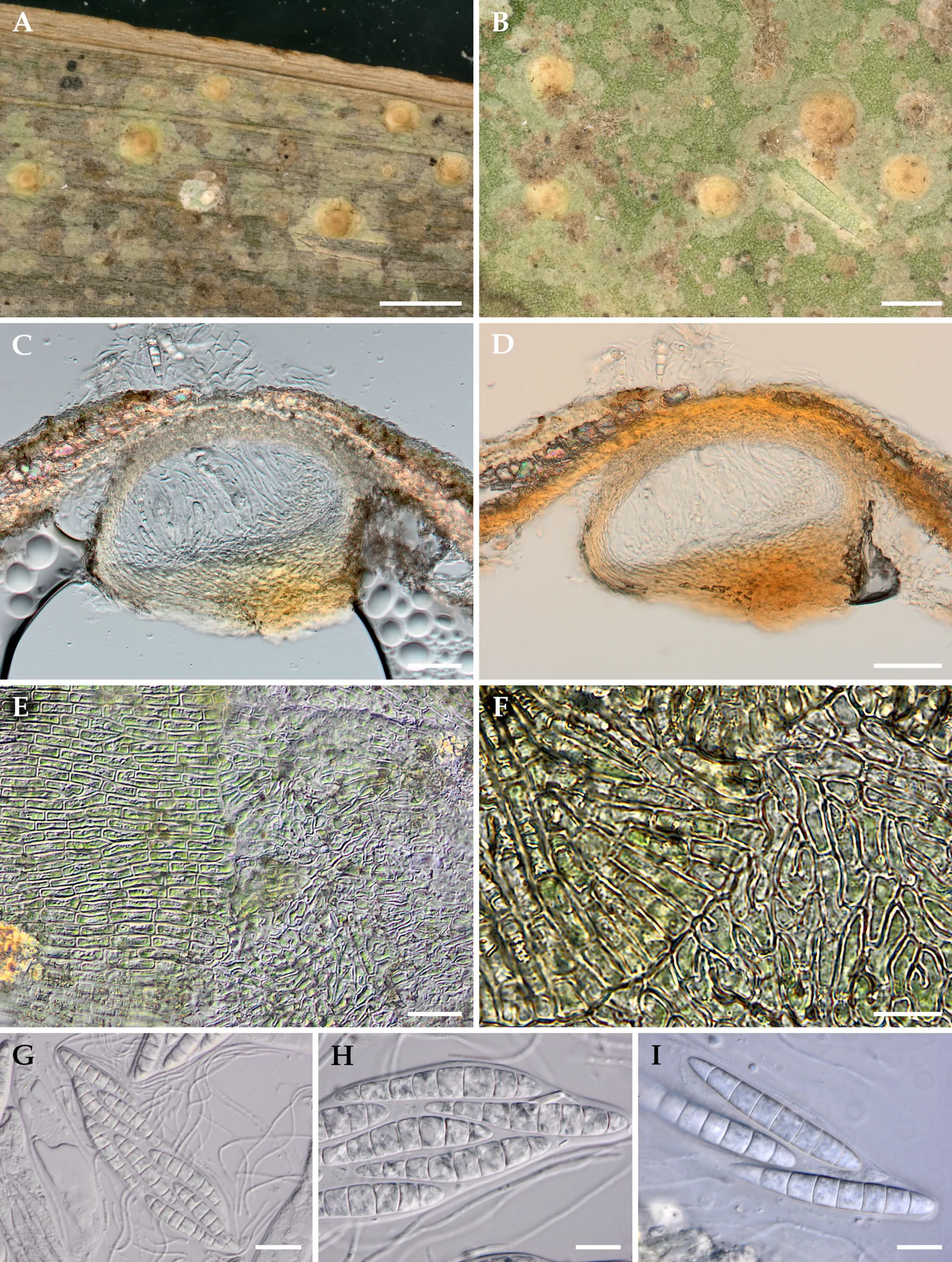
Diagnostic features of *Porina mayottensis* (Mayotte). **A** Dispersed thallus with lens-shaped to hemispherical, orange perithecia (22069A) **B** Dispersed thallus with lens-shaped to hemispherical, pale yellow perithecia (22081A) **C** Perithecial cross-section in water, showing the algal layer on top, the crystallostratum, and the pale yellow involucrellum and excipulum (22069A) **D** Perithecial cross-section in K, showing a K+ pale to sharp orange reaction of the excipulum and involucrellum (22069A) **E** Photobiont in water, showing both angular-rounded cells, arranged irregularly, and rectangular cells, arranged in radiate plates, on the same thallus; calcium oxalate crystals incrusted in thallus (22079B) **F** Close-up of photobiont in water, showing both angular-rounded cells, arranged irregularly, and rectangular cells, arranged in radiate plates, on the same thallus (22085A) **G** Ascus in water, exhibiting eight ascospores and an apical ring (21972B) **H** Ascospores in water (21972B) **I** Ascospores in water (22321D). Scale bars: 1 mm (**A**); 0.5 mm (**B**); 20 µm (**C–E, G**); 10 µm (**F, H, I**).

The examined *P. mayottensis* specimens were recovered in the *Porina* s. str. clade, showing little intraspecific genetic divergence in both the ITS and mtSSU phylogenies (Figs 2, 3). In the ITS phylogeny, they form a subclade with *P. mazosioides* and *P. aff. mazosioides*. *Porina mazosioides* was the most similar species because it shows both morphological similarity and a close phylogenetic relationship as the sister taxon of *P. mayottensis*. However, the two species differ mainly in their perithecial and thallus morphology, photobiont morphology, and slight differences in ascospore sizes. Both angular-rounded photobiont cells arranged irregularly and rectangular cells arranged in radiate plates were observed on five of the eight sequenced specimens. This resembles *P. rudiuscula* (Nyl.) Müll.Arg., in which foliicolous specimens described by Lücking and Vězda (1998) also exhibit different photobiont morphotypes on the same thallus. However, it remains unclear whether these contrasting morphotypes represent two or more *Trentepohlia* species occurring within the same thallus or morphological variation within a single algal species. Although two different *rbc*L sequences were not recovered from individual specimens, several samples consistently produced two PCR fragments of similar length that could not be separated using standard amplification protocols. This anomaly could potentially indicate the presence of two different *Trentepohlia* taxa in the thallus piece used for extraction. Overall, the recovered algal haplotypes were distributed among clades 29 (29:5 and 29:6; Fig. 11E) and 36 (Fig. 11F), the latter containing only sequences from *P. mayottensis* specimens (Figs 4, 5). The sequence recovered within algal haplotype 29:5 clusters with a *P. parva* specimen, which is plausible because the *P. mayottensis* specimen appeared to harbour only a photobiont with rectangular cells arranged in radiate plates. Most specimens had relatively small thalli, although one specimen had a larger, confluent thallus. The combination of a distinct phylogenetic position, consistent morphology across specimens, and diagnostic differences supports the recognition of *P. mayottensis* as a distinct species.

##### Additional specimens examined.

MAYOTTE – **South of Tsararano** • ridge between Mount Bénara and Mount Bépilipili, 12°52'49"S, 45°09'59"E, alt. 580 m, humid ridge forest (mountainous) with rocky outcrops, 03 Oct. 2017; Ertz 22069A (BR). • ibid., summit of Mount Bénara, 12°52'47"S, 45°09'46"E, alt. 660 m, humid ridge forest (mountainous), 01 Oct. 2017; Ertz 21972B (BR). • ibid., summit of Mount Bépilipili, 12°52'52.2"S, 45°10'09.2"E, alt. 659 m, humid montane forest, 03 Oct. 2017; Ertz 22079B (BR). – **Choungui** • Path of Mount Choungui, 12°57'38.7"S, 45°08'08.4"E, alt. 365 m, dense humid forest, 29 Sep. 2017; Ertz 21856D (BR). – **Mamoudzou** • forest reserve of Majimbini, near the governor’s house, 12°45'59.8"S, 45°11'23.7"E, alt. 470 m, dense primary forest, 10 Oct. 2017; Ertz 22321D, 22321E (BR). – **North West of Bandrele** • near summit of Mount Bépilipili, 12°52'54.8"S, 45°10'09.7"E, alt. 611 m, humid montane forest, 01 Oct. 2017; Ertz 22085A (BR).

#### Porina mazosioides

Taxon classificationFungiPertusarialesPertusariaceae

Lücking & Vězda, Willdenowia 28(1/2): 210. 1998.

25E7F55F-E1AB-5B4A-8D98-3A684C5B8E7B

Fig. 16B, C

##### Type.

TANZANIA • N-Uluguru Mountains; 1700–1850 m; mossy forest; Dec. 1972; Pócs et al. 6851 (holotype: PRA).

##### Notes.

*Porina mazosioides* is known from tropical East Africa and the South-Western Indian Ocean region, primarily in lower montane rainforests (Lücking and Vězda 1998). It has been reported from Madagascar (Aptroot 2016) and Mauritius (Ertz and Diederich 2022). This taxon is represented by five specimens from northern, southern, and central Madagascar (20270J, 25340E, 25329D, 27195P, and 27189R) and ten from Mauritius (23459A, 23539E, 23909F, 23909G, 23909H, 23981A, 24006D, 24033A, 24305E, and 24305F), all of which are consistent with the original description by Lücking and Vězda (1998). The ascospores are 7-septate, measuring (38–)43–51(–57) × (3.7–)5–6.2(–7.2) µm (n = 110), broadly matching the reported size of 40–50 × 5–6 µm. Furthermore, the thallus and perithecia morphology matched the reported description of a finely verrucose thallus and hemispherical to wart-shaped perithecia measuring 0.4–0.6 mm. In the mtSSU phylogeny, specimens from Mauritius and Madagascar cluster with the *P. mazosioides* sequences reported by Ertz and Diederich (2022) (Fig. 2). *Porina mazosioides* was the most frequently sequenced species in this study and appeared to be the most abundant *Porinaceae* species in Mauritius. This species exhibited high photobiont selectivity and was exclusively associated with algal haplotype 33:1 (Figs 4, 5). Overall, because the specimens match the original description of *P. mazosioides* and cluster with published accessions, they can be confidently assigned to *P. mazosioides* s. str.

##### Specimens examined.

MADAGASCAR – **Toliara Prov**. • ca. 25 km Northwest of Fort-Dauphin (Tolagnaro), National Park of Andohahela, South part of parcel 1, ca. 5 km West of the village of Malio, 24°55'58"S, 46°44'07"E (ca. 200 m), 600–730 m, humid forest on the slopes of an inselberg, 29 Nov. 2019; Ertz 25340E (BR, TAN). • ibid., ca. 26 km Northwest of Fort-Dauphin (Tolagnaro), National Park of Andohahela, South part of parcel 1, ca. 6 km West of the village of Malio, 24°55'46.9"S, 46°43'55.9"E, alt. 515 m, dense humid forest, 28 Nov. 2019; Ertz 25329D (BR, TAN). – **Diego Suarez Prov**. • Antsiranana, Region: Diana, District: Diego II, Commune: Joffreville, Forontany: Morafeu, National Park of Montagne d’Ambre, Gîte des Roussettes, mixed open woodland, around house and picnic area, 12°31'37"S, 49°10'18"E, alt. 1068 m, 6 Oct. 2014; Ertz 20270J (BR, TAN). – **National Park of Ranomafana** • trail from the Vatoharanana camp towards the park entrance located along the RN25, 21°15'47.3"S, 47°25'20.4"E, alt. 990 m, dense humid mid-altitude and ridge forest, 31 Oct. 2022; Ertz 27195P (BR, TAN). • ibid., 21°16'24.9"S, 47°25'35.8"E, alt. 1115 m, dense humid mid-altitude and ridge forest, 31 Oct. 2022; Ertz 27189R (BR, TAN). – MAURITIUS – **Rivière Noire District** • Black River Gorges National Park, along trail from Plaine Champagne towards Piton de la Petite Rivière Noire, 20°24'52"S (ca. 1000 m), 57°24'43"E (ca. 500 m), alt. 630–800 m, dense evergreen mountainous forest, on the bark of tree, 07 Sep. 2019; Ertz 23909F, 23909G, 23909H, 23981A, 24006D, 24033A (BR). • ibid., 5 km NW of Pétrin, Brise Fer Forest, 20°22'40"S (ca. 200 m), 57°26'25"E (ca. 200 m), 585 m, 10 Sep. 2019; Ertz 24305E, 24305F and Diederich (BR). – **Savanne District** • Black River Gorges National Park, along the trail to Mt Cocotte, 20°26'30"S (ca. 200 m), 57°28'16"E (ca. 500 m), alt. 720–750 m, 01 Sep. 2019; Ertz 23539E and Diederich (BR). – **Plaines Wilhems District** • Black River Gorges National Park, Le Pétrin, along the trail west of Pétrin Information Centre, up to 600 m W of first viewpoint, 20°24'01"S (ca. 200 m), 57°27'30"E (ca. 400 m), 620–660 m, 31 Aug. 2019; Ertz 23459A and Diederich (BR).

#### Porina aff. mazosioides

Taxon classificationFungiPertusarialesPertusariaceae

Lücking & Vězda, Willdenowia 28(1/2): 210. 1998.

832ABA54-0BB3-50E2-B937-C9A138D66DDB

Fig. 16D

##### Type.

TANZANIA • N-Uluguru Mountains; 1700–1850 m; mossy forest; Dec. 1972; Pócs et al. 6851 (holotype: PRA).

##### Notes.

This taxon is represented by two specimens, one from southern Madagascar (25321F) and one from northern Madagascar (20280G). Both specimens are recovered in the *Porina* s. str. clade, and their mtSSU sequences cluster closely with a sequence from *P. longispora* Vězda, reported by Ertz and Diederich (2022) (Fig. 2). However, they differ markedly from *P. longispora* in possessing 7-septate ascospores rather than the characteristic 15-septate ascospores described by Lücking and Vězda (1998). In addition, the specimens feature irregularly arranged, angular-rounded photobionts, whereas Lücking and Vězda (1998) and Ertz and Diederich (2022) describe rectangular photobionts in radiate plates for *P. longispora*. Morphologically, the specimens most closely resemble *P. mazosioides*, but also differ in photobiont morphotype. The ascospores are 7-septate, measuring (42.5–)47.5–53.5(–55.5) × (5.3–)5.6–6.3(–6.7) µm (n = 36), slightly longer than the 40–50 × 5–6 µm reported for *P. mazosioides* by Lücking and Vězda (1998). Furthermore, the thallus appears less rough, whereas the perithecial surface is more rugose than in *P. mazosioides*. *Porina aff. mazosioides* is exclusively associated with algal clade 35 and is the only sequenced taxon assigned to this clade within the *rbc*L phylogeny (Figs 4, 5). Overall, this taxon shows morphological similarity to *P. mazosioides* and phylogenetic affinity to *P. longispora*, while differing from both taxa in phenotypic characters. Pending additional sampling and type-linked sequences, they are therefore retained as *P. aff. mazosioides*.

##### Specimens examined.

MADAGASCAR – **Diego Suarez Prov**. • Antsiranana, West of Sambava, Marojejy National Park, along trail from Camp Marojejia down to Camp Mantella, rather dense humid evergreen lower montane forest, 14°26'07"S, 49°46'05"E, alt. ca. 650 m, 23 Oct. 2014; Ertz 20280G (BR, TAN). – **Toliara Prov**. • ca. 26 km Northwest of Fort-Dauphin (Tolagnaro), National Park of Andohahela, South part of parcel 1, ca. 6 km West of the village of Malio, 24°55'46.9"S, 46°43'55.9"E, alt. 515 m, dense humid forest, 28 Nov. 2019; Ertz 25321F (BR, TAN).

#### Porina parva

Taxon classificationFungiPertusarialesPertusariaceae

Debaene, Diederich & Ertz sp. nov.

2E6F1894-3D28-5866-93AF-1AB58E41EB80

865738

Fig. 12

##### Type.

MADAGASCAR • Toliara Province, ca. 25 km Northwest of Fort-Dauphin (Tolagnaro), National Park of Andohahela, South part of parcel 1, ca. 5 km West of the village of Malio, 24°55'43.4"S, 46°44'37.2"E, alt. 360 m, dense and humid forest at the bottom of a valley near a river bordered by large granite boulders, 27 Nov. 2019; Ertz 25301E (holotype: BR5030171996852; isotype: TAN).

##### Etymology.

The epithet refers to the small thallus and perithecia of the species.

##### Diagnosis.

This species differs from *Porina epiphylloides* Vězda in its small thallus, often scattered in patches; in usually larger perithecia (0.2–0.4 mm in diam. in *P. parva* vs. 0.24–0.25 mm in *P. epiphylloides*); the presence of pycnidia; in larger asci (65–100 × 17–31 µm vs. 65–70 × 8–10 µm); and in ascospores, which are slightly longer and wider (26.5–42 × 3–6.2 µm in *P. parva* vs. 26–34 × 3–3.5 µm in *P. epiphylloides*).

##### Description.

***Thallus*** epiphyllous; mostly in small, irregular patches and sometimes confluent, ca. 0.5–5 mm wide, and ca. 15–25 µm thick; rarely with a whitish translucent prothallus; rugose to distinctly verrucose, verrucae ca. 0.05 mm diam., encrusted with calcium oxalate crystals; matt to slightly glossy; greyish green to yellowish green or green with orange tinge. ***Photobiont*** rectangular, forming radiate plates; ca. (8–)10–14(–18) × (3–)4–6(–7) µm (n = 168). ***Perithecia*** superficial, not well delimited from the thallus; immature perithecia lens-shaped, mature perithecia lens-shaped to hemispherical (rarely almost wart-shaped); surface smooth to slightly rough, glabrous; ca. 0.2–0.4 mm diam. (n = 48), and ca. 140–160 µm high; covered by a ca. 5–10 µm algiferous thallus layer; concolorous to slightly more yellowish than the thallus or pale orange to brown; apex slightly applanate to convex; ostiole inconspicuous. ***Crystallostratum*** ca. 25–50 µm thick. ***Excipulum*** ca. 15–20 µm thick; hyaline, K+ pale yellow. ***Involucrellum*** ca. 20–35 µm thick; hyaline to greyish brown, K+ yellow to orange. ***Hamathecium*** hyaline; not inspersed; composed of thin, simple paraphyses ca. 1–2.5 μm diam., and ca. 70–100 µm long. ***Asci*** fusiform to obclavate; (65–)76–90(–100) × (17–)20–26(–31) µm (n = 31); 8-spored; with apical ring structure. ***Ascospores*** 7-septate, oblong to narrowly fusiform (26.5–)32–38(–42) × (3–)4.1–5.3(–6.2) µm, ratio L/B (5.2–)6.3–8.8(–12.7) (n = 257), gelatinous sheath not or poorly visible. ***Pycnidia*** ca. 0.1 mm diam.; lens-shaped; smooth, glabrous; concolorous to yellowish. ***Conidiophores*** reduced to the conidiogenous cell; conidiogenesis enteroblastic, phialidic; conidiogenous cells elongate ampulliform, 5–6 × 3–3.5 µm. ***Microconidia*** ellipsoid to fusiform; (2.5–)2.6–3.3(–3.6) × (0.8–)0.9–1.2(–1.3) µm (n = 21). ***Chemistry*** not tested for thallus; TLC not performed.

##### Distribution and ecology.

The species is known from three localities in Mauritius and three in southern Madagascar. It inhabits leaf surfaces in dense, humid, evergreen forests, often near rivers, at low to moderate elevations (ca. 345–1000 m).

##### Notes.

**Figure 12.**
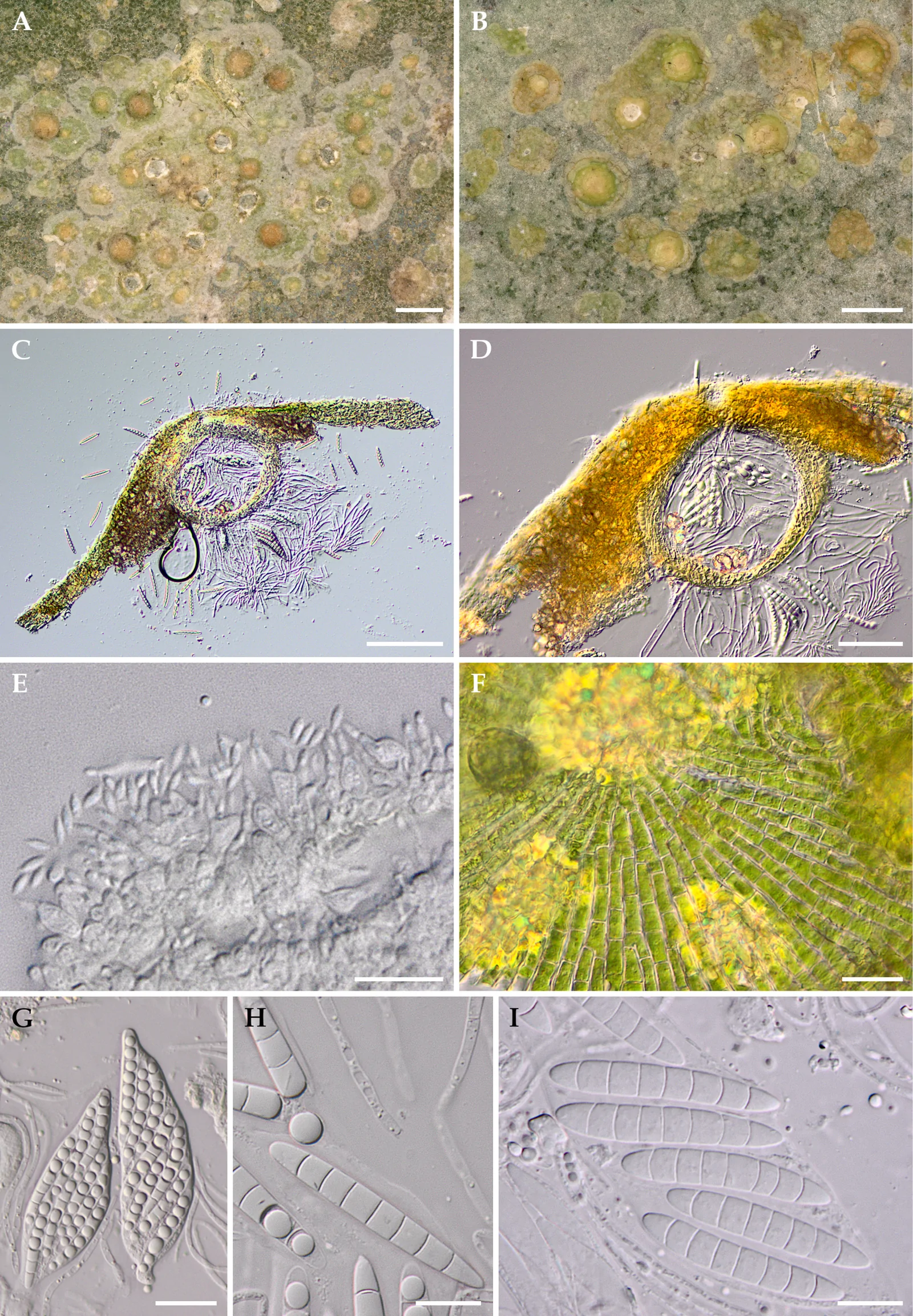
Diagnostic features of *Porina parva*. **A** Confluent thallus with lens-shaped to hemispherical perithecia and pycnidia (25301E, Madagascar) **B** Dispersed thallus with lens-shaped to hemispherical perithecia (23968B, Mauritius) **C** Perithecial cross-section in water, showing the algal layer on top, the crystallostratum, and the greyish brown involucrellum and hyaline excipulum (23968B, Mauritius) **D** Perithecial cross-section in K, showing a K+ yellow to orange reaction in the inner layers (23968B, Mauritius) **E** Microconidia with conidiophores (25301E, Madagascar) **F** Photobiont in water, showing rectangular cells arranged in radiate plates, and calcium oxalate crystals incrusted in thallus (24305I, Mauritius) **G** Asci (fresh material) in water, exhibiting eight ascospores and an apical ring (24305I, Mauritius) **H** Ascospore in water (23968B, Mauritius) **I** Ascospores in water (25301E, Madagascar). Scale bars: 0.5 mm (**A, B**); 100 µm (**C**); 50 µm (**D**); 10 µm (**E, H, I**); 20 µm (**F, G**).

*Porina parva* was recovered as a distinct lineage within the *Porina* s. str. clade in both the mtSSU and ITS phylogenies, although it exhibited a relatively high intraspecific genetic divergence compared with other members of the clade (Figs 2, 3). The species is represented by six specimens from Mauritius and four from southern Madagascar, all of which are morphologically similar. *Porina epiphylloides* is considered the most similar species based on both morphology and phylogenetic affinity. However, *P. parva* differs in possessing generally larger perithecia, considerably larger asci, slightly longer and broader ascospores, and pycnidia, which have not been reported for *P. epiphylloides*. Most specimens formed small, discrete thalli bearing only a few perithecia, although two confluent specimens produced pycnidia, 25301E (Fig. 12A) and 25309C. Better-preserved material often exhibited an orange tinge on the thallus, caused by photobiont pigmentation (Fig. 12B). *Porina parva* showed high photobiont flexibility and was associated with five algal haplotypes distributed across two major *rbc*L clades (29:2, 29:3, 29:4, 29:5, and 33:1) (Figs 4, 5). The combination of a distinct phylogenetic position, consistent morphology across specimens, and diagnostic differences supports the recognition of *P. parva* as a distinct species.

##### Additional specimens examined.

MADAGASCAR – **Toliara Prov**. • ca. 25 km Northwest of Fort-Dauphin (Tolagnaro), National Park of Andohahela, South part of parcel 1, ca. 5 km West of the village of Malio, 24°55'43.4"S, 46°44'37.2"E, alt. 360 m, dense and humid forest at the bottom of a valley near a river bordered by large granite boulders, 27 Nov. 2019; Ertz 25301D (BR, TAN) • ibid., ca. 26 km Northwest of Fort-Dauphin (Tolagnaro), National Park of Andohahela, South part of parcel 1, ca. 6 km West of the village of Malio, 24°55'46.9"S, 46°43'55.9"E, alt. 515 m, dense and humid forest, 28 Nov. 2019; Ertz 25309C (BR, TAN). • ibid., ca. 6.5 km West of the village of Malio, 24°56'24.1"S, 46°43'26.2"E, alt. 1000 m, dense humid forest, 01 Dec. 2019; Ertz 25411B (BR, TAN). – MAURITIUS – **Rivière Noire District** • Black River Gorges National Park, 5 km North-West of Pétrin, Brise Fer Forest, 20°22'40"S (ca. 200 m), 57°26'25"E (ca. 200 m), 585 m, 10 Sep. 2019; Ertz 24305I (BR, MAU), 24305G (BR) and Diederich. • ibid., along trail from Plaine Champagne towards Piton de la Petite Rivière Noire, 20°24'52"S (ca. 1000 m), 57°24'43"E (ca. 500 m), alt. 630–800 m, dense evergreen mountainous forest, 07 Sep. 2019; Ertz 23965A (BR), 23968B (BR, MAU), 23981D (BR). • ibid., Chamarel, Ebony Forest, South-West part of forest, 20°26'13"S (ca. 50 m), 57°22'06"E (ca. 50 m), alt. 345 m, 02 September 2019; Ertz 23650H and Diederich (BR).

#### Porina rubentior

Taxon classificationFungiPertusarialesPertusariaceae

(Stirt.) Müll. Arg., Flora, Regensburg 66(2): 26. 1883.

F16C4E5C-9A0A-5F1F-9BEA-156BE823DE6B

Fig. 18B

##### Type.

BRAZIL • Trail (lectotype: BM, *fide* R.Lücking, Bot. Acta 109: 259 (1996)).

##### Notes.

*Porina rubentior* is a pantropical species commonly found in shady rainforest understories or drier evergreen forests (Lücking 2008). It has previously been reported in Madagascar by Aptroot (2016). This taxon is represented by three examined specimens from northern and southern Madagascar (20277H, 25411F, and 25411G), all of which agree well with the descriptions provided by Lücking (1996, 2008). The 3-septate ascospores measure (15–)16–18.5(–19.5) × (2.2–)2.4–2.9(–3) µm (n = 16), consistent with the reported range of 14–21 × 2.5–3.5 µm. The lens-shaped, dark red to blackish red perithecia measure 0.15–0.2 mm (n = 8), matching both the reported morphology and dimensions of 0.15–0.2(–0.3) mm. In the mtSSU phylogeny, the specimens were recovered within the *Phragmopeltheca* clade and show very little genetic divergence from Neotropical *P. rubentior* accessions (Fig. 2). No *rbc*L sequence was recovered for this taxon, although the photobiont exhibits the morphotype with rectangular cells arranged in radiate plates. Overall, the close agreement between morphology and phylogenetic placement supports the confident assignment of these specimens to *P. rubentior* s. str. under the current species concept.

##### Specimens examined.

MADAGASCAR – **Diego Suarez Prov**. • Antsiranana, West of Sambava, Marojejy National Park, along trail from Camp Mantella to Camp Marojejia, rather dense humid evergreen lower montane forest, 14°26'07"S, 49°46'05"E, alt. ca. 650 m, 18 Oct. 2014; Ertz 20277H (BR, TAN). – **Toliara Prov**. • ca. 26 km Northwest of Fort-Dauphin (Tolagnaro), National Park of Andohahela, South part of parcel 1, ca. 6.5 km West of the village of Malio, 24°56'24.1"S, 46°43'26.2"E, alt. 1000 m, dense humid forest, 01 Dec. 2019; Ertz 25411F, 25411G (BR, TAN).

#### Porina rufula

Taxon classificationFungiPertusarialesPertusariaceae

(Kremp.) Vain., Acta Soc. Fauna Flora fenn. 7(no. 2): 227. 1890.

2BD30270-1834-5FBB-A0DF-77C1DEFB08C1

Fig. 18C, D

##### Type.

MALAYSIA • Sarawak [Borneo]; 1866; Beccari (holotype: M[M0023867]; isotype: G[G00293679]).

##### Notes.

New record for Mayotte. *Porina rufula* is a pantropical species and among the most common members of the genus in rainforest understories (Lücking 2008). It has previously been reported in Mauritius by Ertz and Diederich (2022). This taxon is represented by three examined specimens, two from Mayotte (21936A and 21938A) and one from Mauritius (23539D), all of which agree well with the descriptions provided by Lücking (1996, 2008). The 3-septate ascospores measure (21–)22.5–26.5(–30) × (3.3–)3.6–4.4(–4.8) µm (n = 49), matching the reported range of 18–27(–31) × 3–5(–6) µm. The lens-shaped, cherry-red to orange perithecia measure 0.2–0.3 mm (n = 10), matching the reported morphology and dimensions of 0.15–0.3 (–0.4) mm. In the mtSSU phylogeny, the examined specimens were recovered within the *Phragmopeltheca* clade and cluster with both Neotropical and Paleotropical *P. rufula* accessions, although they show closer affinity to the Paleotropical lineage (Fig. 2). Within this lineage, the two Mayotte specimens (Fig. 18C) form a distinct subgroup, whereas the Mauritian specimen (Fig. 18D) clusters with a Japanese accession. *Porina rufula* exhibits one of the highest levels of intraspecific mtSSU divergences recovered in this study, exceeding that observed for the ITS marker. Both Mayotte specimens showed high specificity and were exclusively associated with algal haplotype 33:2, whereas the Mauritian specimen was associated with one of the most shared algal haplotypes, 33:1 (Figs 4, 5). Overall, the close agreement with the published morphology and the phylogenetic placement of these specimens support their confident assignment to *P. rufula* s. str. under the current species concept, although the marked phylogeographic structure indicates that species boundaries within this complex warrant further investigation.

##### Specimens examined.

MAYOTTE – **South of Tsararano** • trail of Mount Bénara, 12°52'44.0"S, 45°09'37.5"E, alt. 560 m, dense humid hillside forest, 01 Oct. 2017; Ertz 21936A (BR). • ibid., 12°52'45.2"S, 45°09'39.0"E, alt. 575 m, dense humid hillside forest, 01 Oct. 2017; Ertz 21938A (BR, MAO). – MAURITIUS – **Savanne District** • Black River Gorges National Park, along the trail to Mt Cocotte, 20°26'30"S (ca.200 m), 57°28'16"E (ca.500 m), 720–750 m, 01 Sep. 2019; Ertz 23539D and Diederich (BR).

#### Porina sphaerocephala

Taxon classificationFungiPertusarialesPertusariaceae

Vain., Ann. Acad. Sci. Fenn., Ser. A 15(no. 6): 368. 1921.

75255063-592A-5E4F-9FF6-7FBF7ACE18A9

Fig. 17A, G

##### Type.

PHILIPPINES • Robinson (holotype: TUR–VAINIO).

##### Notes.

*Porina sphaerocephala* is a widely distributed species in Paleotropical rainforests (McCarthy 2013). It has also been reported from Madagascar by Aptroot (2016). This taxon is represented by three examined specimens from southern Madagascar (25321D, 25329C, and 25411C), recovered in the *P. sphaerocephala* clade. These specimens closely match the description provided by McCarthy (2013). The ascospores are 7-septate, measuring (28–)34–38.5(–41) × (3.1–)3.4–4.1(–4.7) µm (n = 46), consistent with the reported size range of 27–40 × 3–4.5 µm. Furthermore, the perithecia, 0.1–0.2 mm (n = 6), matched the reported subglobose, pale yellowish to pale orange-brown perithecia measuring 0.15–0.28 mm. However, distinct thalline ridges, sometimes associated with this species, were not observed in the specimens. In the ITS and mtSSU phylogenies, the examined specimens formed a well-supported clade sister to *P. sphaerocephaloides*, with little intraspecific genetic divergence (Figs 2, 3). A single *rbc*L sequence was recovered for this species, indicating an association with algal haplotype 29:1 (Figs 4, 5). Overall, the specimens closely match the distinctive morphology described for *P. sphaerocephala*. Although no comparative accessions of the species are currently available, no other described species exhibits this combination of characters, making an alternative identification unlikely. The specimens can therefore be confidently assigned to *P. sphaerocephala* s. str. and represent the first sequenced specimens.

##### Specimens examined.

MADAGASCAR – **Toliara Prov**. • ca. 26 km northwest of Fort-Dauphin (Tolagnaro), National Park of Andohahela, South part of parcel 1, ca. 6 km West of the village of Malio, 24°55'46.9"S, 46°43'55.9"E, alt. 515 m, dense humid forest, 28 Nov. 2019; Ertz 25321D, 25329C (BR, TAN). • ibid., South part of parcel 1, ca. 6.5 km West of the village of Malio, 24°56'24.1"S, 46°43'26.2"E, alt. 1000 m, dense humid forest, 01 Dec. 2019; Ertz 25411C (BR, TAN).

#### Porina sphaerocephaloides

Taxon classificationFungiPertusarialesPertusariaceae

Farkas, Lichenologist 19(1): 54. 1987.

4E6566C9-81D3-5AD5-B4EA-E16F4B540446

Fig. 17B–F

##### Type.

TANZANIA • West Usambara Mts, Mazumbai University Forest reserve, in rocky Sagara Ridge in fragments of mossy forest on *Zenkerella grotei* leaves at 1820 m alt., 23 Jan. 1985, T.Pócs 8518/AD (holotype: VBI).

##### Notes.

*Porina sphaerocephaloides* is a common species in tropical Africa (Farkas 1987). It has also been reported from Madagascar by Aptroot (2016). This taxon is represented by 13 examined specimens from northern, southern, and central Madagascar (20270F, 20272G, 20272H, 20274E, 20275B, 20277G, 20279L, 20279M, 20279Q, 20280D, 25373B, 25375G, and 27163C), which were recovered in the *P. sphaerocephala* clade. Most specimens match the original description by Farkas (1987), although they exhibit substantially greater variation in ascospore morphology than previously reported. The perithecia, 0.25–0.35 mm (n = 39), matched the reported globose, constricted-at-the-base, rough and unequally thickened, yellow to pale ochraceous yellow perithecia measuring 0.25–0.4 mm (Fig. 17B). The distinct thalline verrucae, regarded as characteristic of this species, were reduced or absent in some specimens. In contrast to the relatively conserved external morphology, ascospore morphology proved to be highly variable. Although some specimens appeared to have exclusively 7-septate ascospores, others harboured a minority (ca. 10–20%) of multi-septate ascospores, ranging from 8- to 12-septate, alongside 7-septate ascospores. Ascospore length, width, and degree of constriction likewise varied considerably among specimens (Fig. 17E, F). An exceptionally divergent specimen (20279M) harboured exclusively 13–15-septate ascospores (Fig. 17D) and perithecia that appeared slightly more uneven and verrucose (Fig. 17C) relative to others (Fig. 17B). Its ascospore measurements were analysed separately from the remaining specimens because they fell well outside the observed range, measuring (63–)68–78(–90) × (5.3–)5.7–6.7(–7.6) µm (n = 58). The ascospores of the other examined specimens measured (35–)44.5–61.5(–75) × (3.7–)5–7.9(–11) µm (n = 253), broadly matching the reported 7(–9)-septate ascospores of 42–60 × 6–9 µm, despite several extreme measurements. Despite this pronounced variation in ascospores morphology, neither phylogeny supported a corresponding species-level subdivision. Both mtSSU and ITS markers recovered *P. sphaerocephaloides* as a distinct clade with several moderately supported subgroups (Figs 2, 3), but these did not correspond to ascospore morphology or any other consistent phenotypic character. For example, specimen 20272G clustered with the highly divergent specimen (20279M) in the mtSSU phylogeny despite producing exclusively 7-septate ascospores that match the typical size ranges. This taxon showed high photobiont flexibility, associating with four algal haplotypes distributed across two *rbc*L clades (29:1, 33:1, 33:4, and 33:5) (Figs 4, 5). Overall, the relatively conserved external morphology, the absence of phylogenetically structured phenotypic variation, and the distinctive morphology originally described for *P. sphaerocephaloides* support interpreting the examined material as a single morphologically variable lineage. Although no published accessions are currently available for comparison, the distinctive morphology makes confusion with other described species unlikely. These specimens are therefore assigned to *P. sphaerocephaloides* s. str., pending additional sampling, particularly from type-linked material.

##### Specimens examined.

MADAGASCAR – **Diego Suarez Prov**. • Antsiranana, Region: Diana, District: Diego II, Commune: Joffreville, Forontany: Morafeu, National Park of Montagne d’Ambre, Gîte des Roussettes, mixed open woodland, around house and picnic area, 12°31'37"S, 49°10'18"E, alt. 1068 m, 6 Oct. 2014; Ertz 20270F (BR, TAN). • ibid., across stream at small dam to dirt road and trail down to Lake Mahasarika, moist but not wet lower montane forest, with tall trees and tree ferns, 12°32'03"S, 49°10'28"E, alt. 1177 m, 7 Oct. 2014; Ertz 20272G, 20272H (BR, TAN). • ibid., along trail from summit Olioly to small dam near camp Gîte des Roussettes, through moist montane forest, 12°32'34"S, 49°09'23"E, alt. 1242 m, 8 Oct. 2014; Ertz 20274E (BR, TAN). • ibid., West of Sambava, Marojejy National Park, Trail from Park entrance to Camp Mantella, rather dense humid evergreen lower montane forest, 14°26'48"S, 49°47'06"E, alt. 360 m, 17 Oct. 2014; Ertz 20275B (BR, TAN). • ibid., along trail from Camp Mantella to Camp Marojejia, rather dense humid evergreen lower montane forest, 14°26'07"S, 49°46'05"E, alt. ca. 650 m, 18 Oct. 2014; Ertz 20277G (BR, TAN). • ibid., along trail from Camp Simpona down to Camp Marojejia, rather dense humid montane forest, 14°26'20"S, 49°45'08"E, alt. ca. 1200 m, 23 Oct. 2014; Ertz 20279L, 20279M, 20279Q (BR, TAN). • along trail from Camp Marojejia down to Camp Mantella, rather dense humid evergreen lower montane forest, 14°26'07"S, 49°46'05"E ca. 650 m, 23 Oct. 2014; Ertz 20280D (BR, TAN). – **Toliara Prov**. • ca. 25 km Northwest of Fort-Dauphin (Tolagnaro), National Park of Andohahela, South part of parcel 1, ca. 5 km West of the village of Malio, 24°56'07.0"S, 46°44'08.5"E, alt. 680 m elev., dense humid forest, 29 Nov. 2019; Ertz 25373B, 25375G (BR, TAN). – **Ranomafana National Park** • trail along the Vatoharanana river from the Vatoharanana camp, 21°17'08.0"S, 47°26'00.7"E, alt. 1015 m, dense humid forest at medium altitude, 30 Oct. 2022; Ertz 27163C (BR, TAN).

###### *Porina aff. subepiphylla* 1 & 2 Lücking & Vězda, Willdenowia 28(1/2): 217. 1998.

Fig. 15E, F

**Type**. COSTA RICA • Heredia, La Selva Protection Zone, 10°26'N, 84°03'W, 50 m, lowland rain forest, 9.1991, Lücking 91-5751 (holotype: CR).

**Notes**. *Porina aff. subepiphylla* 1 (Fig. 15E) is represented by specimens 20272E and 20271H, whereas *P. aff. subepiphylla* 2 (Fig. 15F) is represented by a single specimen (20272D), all collected from northern Madagascar. Both taxa most closely resemble *P. subepiphylla* as originally described by Lücking and Vězda (1998). Their 7-septate ascospores measure (36.5–)37–41.5(–43) × (3.9–)4.3–4.9(–5.2) µm (n = 40) in *P. aff. subepiphylla* 1 and (28.5–)29.5–35(–36.5) × (4.1–)4.2–4.8(–5.4) µm (n = 58) in *P. aff. subepiphylla* 2, broadly matching the reported range for *P. subepiphylla* (30–43 × 4.5–6 µm). Both taxa also resemble *P. subepiphylla* in possessing wart-shaped perithecia with a slight reddish tinge, but differ in having larger perithecia than the reported range of 0.3–0.4 mm. Perithecia measure 0.45–0.7 mm (n = 36) in *P. aff. subepiphylla* 1 and 0.35–0.55 mm (n = 15) in *P. aff. subepiphylla* 2. Altogether, the two taxa differ from one another, showing only partial overlap in ascospore dimensions and perithecial size. In the ITS and mtSSU phylogenies, the taxa are recovered within the *Phylloporina* clade but form two distinct lineages (Figs 2, 3). *Porina aff. subepiphylla* 1 clusters with *P. complanata* specimens, despite their markedly different morphology. By contrast, all other available mtSSU accessions identified as *P. subepiphylla*, including Neotropical material from Costa Rica, the type country, are recovered within the *Porina* s. str. clade rather than the *Phylloporina* clade. Although these sequences do not originate from the type specimen or type locality, they provide stronger support for the current concept of *P. subepiphylla* than the specimens from Madagascar examined here (Baloch and Grube 2006, 2009). A higher level of symbiont flexibility was observed in *P. aff. subepiphylla* 2, associating with two haplotypes (26:1 and 26:6) from the same thallus (Figs 4, 5). By contrast, only one haplotype (26:1) was found to be associated with both *P. aff. subepiphylla* 1 specimens. Overall, the morphological and molecular evidence supports recognition of two distinct lineages showing affinity to *P. subepiphylla*. However, available evidence does not justify assigning either lineage to *P. subepiphylla* s. str., and they are therefore retained as *P. aff. subepiphylla* 1 and *P. aff. subepiphylla* 2.

**Specimens examined**. MADAGASCAR – **Diego Suarez Prov**. • Antsiranana, Region: Diana, District: Diego II, Commune: Joffreville, Forontany: Morafeu, National Park of Montagne d’Ambre, along trail from Gîte des Roussettes, across stream at small dam to dirt road, moist but not wet lower montane forest, with tall trees and tree ferns, 12°31'55"S, 49°10'33"E, alt. 1119 m, 7 Oct. 2014; Ertz 20271H (BR, TAN). • ibid., across stream at small dam to dirt road and trail down to Lake Mahasarika, moist but not wet lower montane forest, with tall trees and tree ferns, 12°32'03"S, 49°10'28"E, alt. 1177 m, 7 Oct. 2014; Ertz 20272D, 20272E (BR, TAN).

#### Porina trichocoronata

Taxon classificationFungiPertusarialesPertusariaceae

Debaene & Ertz sp. nov.

84ED2B7F-7A47-5124-ABED-85CF027756BC

865742

Fig. 13

##### Type.

MADAGASCAR • Diego Suarez Province. Antsiranana, Region: Diana, District: Diego II, Commune: Joffreville, Forontany: Morafeu, National Park of Montagne d’Ambre, Gîte des Roussettes, mixed open woodland, around house and picnic area, 12°31'37"S, 49°10'18"E, alt. 1068 m, 6 Oct. 2014, Ertz 20270K (holotype: BR5030171997880; isotype: TAN).

##### Etymology.

The epithet refers to the characteristic crown of white hairs near the perithecial apex.

##### Diagnosis.

This species differs from *Porina trichothelioides* R.Sant. in the septation of its ascospores (3–5-septate in *P. trichocoronata* vs. 7-septates in *P. trichothelioides*); in having slightly shorter and narrower ascospores (19–31 × 3.2–5 µm in *P. trichocoronata* vs. 28–32 × 4–5 µm in *P. trichothelioides*); in the overall much thicker perithecial walls which tend to be thicker in the lower part rather than the upper part; in its photobiont morphotype (rectangular and forming distinct radiate plates in *P. trichocoronata* vs. more or less angular in almost continuous plates in *P. trichothelioides*).

##### Description.

***Thallus*** epiphyllous; dispersed to continuous with uneven margins, ca. 7 mm wide, and ca. 10–15 µm thick; slightly rugose, matt; green to yellowish green. ***Photobiont*** rectangular, arranged in radiate plates; ca. (9–)10–13(–14) × (2–)3–4 µm (n = 20). ***Perithecia*** superficial, well delimited from thallus, globose, constricted at base; smooth, matt; ca. 0.1–0.2 mm diam. (n = 13), and ca. 180 µm high; covered up to base with ca. 5–10 µm algiferous thallus layer; greyish black to black; crown of ca. 30 white hairs surrounding apex of perithecium, hairs thick and soft, consisting of agglutinated hyphae, sometimes fused into seta-like structures, ca. 30–100 µm in length and ca. 10–30 µm in width; ostiole inconspicuous, apex rounded, glabrous around ostiole. ***Crystallostratum*** not present. ***Excipulum*** and ***Involucrellum*** indistinguishable from one another, ca. 20–35 µm thick around the apex and ca. 40–50 µm thick at the remaining lower part; greyish blue to black, K-. ***Hamathecium*** hyaline; not inspersed; composed of thin, simple paraphyses ca. 1–2 μm diam., and ca. 80–100 µm long. ***Asci*** obclavate; 52–80 × 10–16 µm (n = 10); 8-spored; with apical ring structure. ***Ascospores*** 3–5-septate, broadly oblong to fusiform, often tapered on one or both sides, (19–)23–27.5(–31) × (3.2–)3.6–4.3(–5) µm, ratio L/B (4.2–)5.7–7.2(–8.3) (n = 101). ***Pycnidia*** not observed. ***Chemistry*** not tested for thallus; TLC not performed.

##### Distribution and ecology.

The species is known from only one locality in northern Madagascar. It inhabits the upper surface of leaves in mixed open woodlands at moderate elevation (ca. 1100 m).

##### Notes.

**Figure 13.**
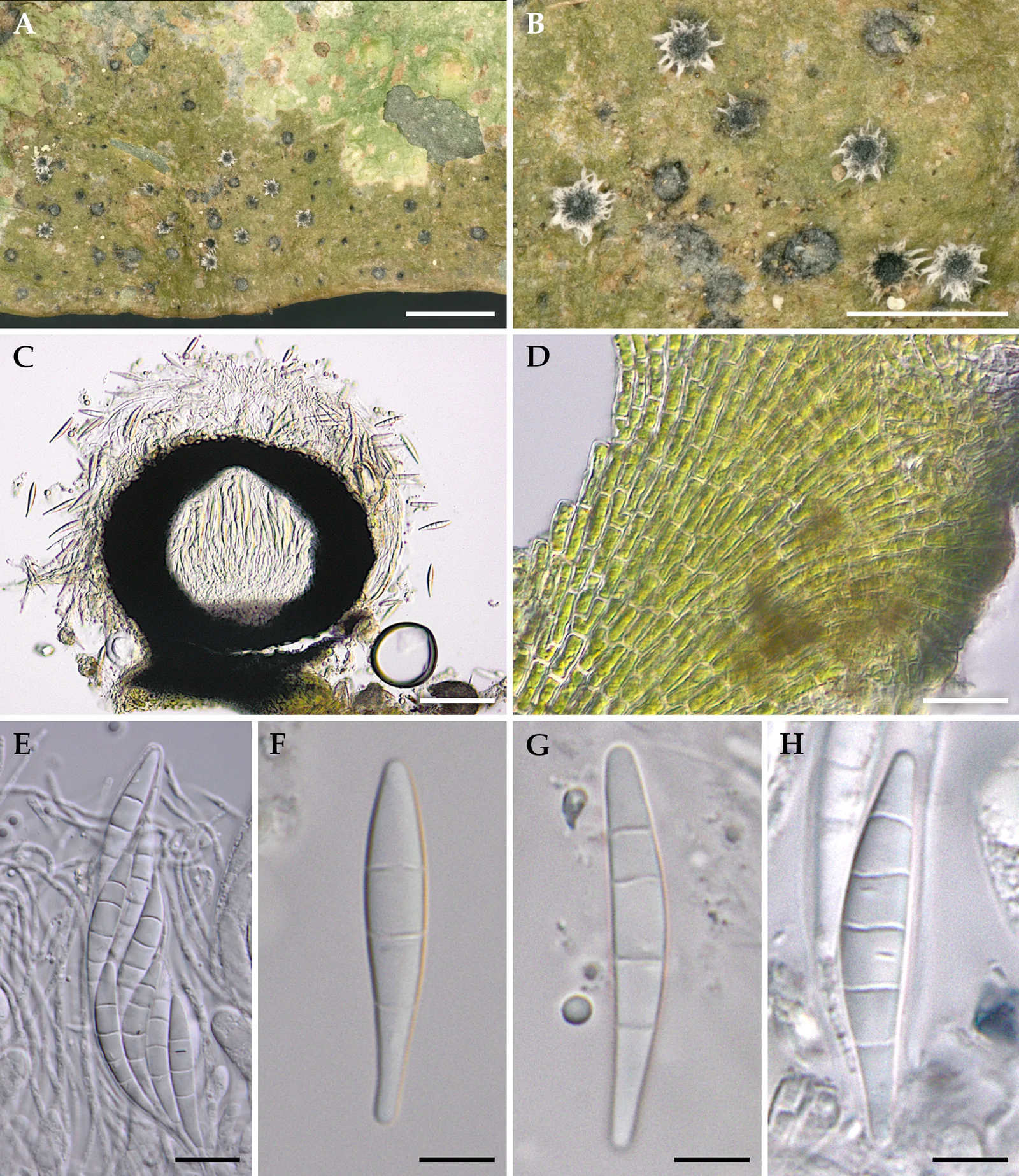
Diagnostic features of *Porina trichocoronata* (20270K, Madagascar). **A** Continuous thallus with perithecia **B** Close–up of globose perithecia **C** Perithecial cross-section in water, showing black, indistinguishable inner layers **D** Photobiont in water, showing rectangular cells arranged in radiate plates **E** Ascus in water, exhibiting eight ascospores and an apical ring **F** 3-septate ascospore in water **G** 4-septate ascospore in water **H** 5-septate ascospore in water. Scale bars: 1 mm (**A**); 0.5 mm (**B**); 50 µm (**C**); 20 µm (**D**); 10 µm (**E**); 5 µm (**F, G, H**).

*Porina trichocoronata* most closely resembles the unsequenced *P. trichothelioides*, originally described by Santesson (1952). Both species share similarly coloured and shaped perithecia bearing a crown of white hairs, although the hairs appear somewhat longer in *P. trichocoronata*. Hair width was not reported for *P. trichothelioides*. The main differences concern the anatomy of the perithecial walls and the ascospores. In both species, the excipulum and involucrellum are not differentiated, but *P. trichothelioides* possesses a thicker black upper half (14–24 µm) and a thinner hyaline to light brown lower half (8–10 µm), whereas *P. trichocoronata* has uniformly greyish-blue to black perithecial walls that are thinner around the apex (20–35 µm) and thicker walls in the remaining lower part (40–50 µm) (Fig. 13C). Furthermore, *P. trichocoronata* has 3–5-septate ascospores that are slightly narrower and shorter than the 7-septate ascospores reported for *P. trichothelioides*. The photobiont morphotype of *P. trichothelioides* cannot be determined with certainty. However, Santesson (1952) described the photobiont as “more or less angular” and arranged in “almost continuous plates”, which most likely corresponds to the angular-rounded, irregularly arranged morphotype recognised in the present study. This differs from the rectangular cells arranged in distinct radiate plates observed in *P. trichocoronata* (Fig. 13D). The new species also resembles *P. perminuta* Vain. in having globose black perithecia bearing hairs (Santesson 1952). However, *P. perminuta* differs in having narrower perithecia with shorter greyish hairs, thinner perithecial walls throughout, and shorter 3-septate ascospores. Although no *rbc*L sequence was recovered for *P. trichocoronata*, the photobiont morphology resembles that of algal clades 33 or 29 (Fig. 6C–F). In the ITS phylogeny, *P. trichocoronata* was recovered within the *Trichothelium* clade (Fig. 3), whereas no mtSSU sequence was obtained. Some perithecia bear partially fused hyphal hairs forming seta-like structures. However, these structures remain soft and only sporadically developed, and are therefore interpreted as agglutinated hyphal hairs rather than true setae as in *Trichothelium alboatrum* Vain. (Santesson 1952). Pending a broader revision of the *Trichothelium* clade, the species is consequently assigned to the genus *Porina*. The combination of a distinct phylogenetic position and diagnostic differences supports the recognition of *P. trichocoronata* as a distinct species.

###### *Porina* sp. 1, 2, 3, 4, & 5

**Notes**. The material assigned to *Porina* sp. 1–5 lacked hymenial structures and ascospores in the examined perithecia, preventing assessment of important diagnostic characters. Moreover, the available morphological characters did not allow confident assignment to the closest named taxa. The five specimens are therefore retained as unnamed, distinct taxa. *Porina* sp. 1–4 were placed within the *Phylloporina* clade, whereas the bambusicolous *Porina* sp. 5 was placed within the *Phragmopeltheca* clade. *Porina* sp. 1 differs from the closely related *P. aff. lucida* 1 in perithecial morphology. Its perithecia are lens-shaped to hemispherical (0.3–0.45 mm; n = 11), with a convex apex, whereas those of *P. aff. lucida* 1 are hemispherical to conical (0.4–0.8 mm; n = 45), with an apex ranging from slightly convex to distinctly applanate and darker in colour. *Porina* sp. 2 is characterised by a smooth, continuous thallus and pale green, lens-shaped to hemispherical perithecia (0.4–0.5 mm; n = 7), with an orange to reddish tinge around the ostiole. Although *Porina* sp. 3 and sp. 4 showed little genetic divergence in the mtSSU phylogeny, they were provisionally treated as separate taxa from one another and from *P. aff. subepiphylla* 2 based on differences in external morphology. *Porina* sp. 3 has a slightly rough, dispersed thallus and orange-green, lens-shaped to hemispherical perithecia (0.25–0.4 mm; n = 14) that are distinctly spaced apart. In contrast, *Porina* sp. 4 has a smooth, continuous thallus and yellowish-green, lens-shaped perithecia (0.3–0.5 mm; n = 21) that are irregularly distributed, closely spaced, and sometimes form nearly contiguous clusters. The bambusicolous specimen designated *Porina* sp. 5 was included because of its placement within the *Phragmopeltheca* clade in the ITS phylogeny. Although bamboo culms can provide ecological conditions comparable to leaf surfaces (Santesson 1952), bambusicolous material was treated as a distinct substrate in this study. The specimen has a yellowish-green, smooth to slightly rough thallus and dark red to blackish-red, lens-shaped perithecia (0.15–0.25 mm; n = 9).

**Specimens examined**. MADAGASCAR – **Diego Suarez Prov**. • Antsiranana, West of Sambava, Marojejy National Park, Camp Mantella area, trail to Cascade de Humbert, through rather dense humid evergreen lower montane forest, stream bank at waterfall of large exposed bedrock area, 14°25'58"S, 49°46'22"E, alt. ca. 480 m, on bamboo culm, 18 Oct. 2014; Ertz 19816B as “*Porina* sp. 5 ” (BR, TAN). • ibid., trail from Park entrance to Camp Mantella, rather dense humid evergreen lower montane forest, 14°26'48"S, 49°47'06"E, alt. 360 m, 17 Oct. 2014; Ertz 20275C as “*Porina* sp. 3 ” (BR, TAN). • ibid., along trail from Camp Simpona down to Camp Marojejia, rather dense humid montane forest, 14°26'20"S, 49°45'08"E, alt. ca. 1200 m, 23 Oct. 2014; Ertz 20279I as “*Porina* sp. 2 ” (BR, TAN). – **Toliara Prov**. • ca. 26 km Northwest of Fort-Dauphin (Tolagnaro), National Park of Andohahela, South part of parcel 1, ca. 6.5 km West of the village of Malio, 24°56'24.1"S, 46°43'26.2"E, alt. 1000 m, dense humid forest, 01 Dec. 2019; Ertz 25411D as “*Porina* sp. 4 ”, 25411E as “*Porina* sp. 1 ” (BR, TAN).

#### Trichothelium aff. argenteum

Taxon classificationFungiOstropalesPorinaceae

Lücking & L.I. Ferraro, Lichenologist 29(3): 217. 1997.

6C206DF1-543B-5C71-BB1D-1607F73B3EFE

Fig. 19D

##### Type.

COSTA RICA • Limón: Hitoy Cerere Biological Reserve, 100–200 m, mar. 1991, Lücking 91–1374 (holotype: ULM).

##### Notes.

This taxon is represented by a single specimen (20277C). Morphologically, it most closely resembles *Trichothelium argenteum* based on the morphology of its setae and ascospores, according to the original description by Lücking and Ferraro (1997). The 7-septate ascospores measure (35–)38–43.5(–46) × (4.9–)5.1–5.9(–6.3) µm (n = 38) and are slightly longer and broader than those reported for *T. argenteum* (30–40 × 4–5.5 µm), although they considerably overlap with the reported range. The black perithecia measure 0.15–0.25 mm (n = 4) in diameter and bear acute and solitary setae, measuring 0.3–0.65 mm (n = 8) in length with black bases and silvery white tips. These characters broadly resemble those of *T. argenteum*, but differ from the original description, in which the perithecia are broader (0.3–0.35 mm) and the setae are basally confluent and form an almost continuous plate in young perithecia, whereas those of the present specimen remain discrete. Furthermore, *T. argenteum* possesses angular-rounded photobiont cells arranged irregularly, whereas the examined specimen has rectangular cells arranged in radiate plates. In the ITS phylogeny, the specimen is recovered within the *Trichothelium* clade (Fig. 3), although no mtSSU sequence was obtained. No sequenced material confidently identified as *T. argenteum* is currently available for phylogenetic comparison. Overall, the specimen shows clear morphological affinity with *T. argenteum* but differs in several consistent characters from the current species concept. Pending additional sampling and, ideally, type-linked sequences, it is therefore retained as *T. aff. argenteum*.

##### Specimens examined.

MADAGASCAR – **Diego Suarez Prov**. • Antsiranana, West of Sambava, Marojejy National Park, along trail from Camp Mantella to Camp Marojejia, rather dense humid evergreen lower montane forest, 14°26'07"S, 49°46'05"E, alt. ca. 650 m, 18 Oct. 2014; Ertz 20277C (BR, TAN).

#### Trichothelium cf. epiphyllum

Taxon classificationFungiOstropalesPorinaceae

Müll. Arg., Bot. Jahrb. Syst. 6: 418. 1885.

B35CC4C6-E64F-5623-971F-DF9E4D83AA8A

Fig. 19A–C

##### Type.

CUBA • Unknown locality, Wright (holotype: G).

##### Notes.

*Trichothelium epiphyllum* has traditionally been regarded as a pantropical species, and specimens attributable to the current concept have previously been reported from Madagascar (Lücking 2008; Aptroot 2016). The present taxon is represented by seven specimens collected from northern and southern Madagascar (20271F, 20272I, 20279F, 20279J, 20279K, 25340B, and 25409B). These specimens broadly agree with the description of *T. epiphyllum* reported by Lücking (2008), although they exhibit some morphological variation. Ascospores are 7-septate and measure (28–)32.5–42(–50) × (3.7–)4.7–6.3(–7.3) µm (n = 125), broadly corresponding to the reported range of 35–45 × 4–5 µm. Both ascospore length and, particularly, width vary among specimens, with some producing distinctly broader spores. Perithecial morphology is likewise variable, especially in the form of the setae, which range from brush-shaped to lanceolate or acute, accurately matching the reported description. Perithecia are subglobose, black, and ca. 0.15–0.25 mm (n = 19) in diameter, while the setae measure ca. 0.2–0.4 mm (n = 32) in length. Altogether, these characters correspond closely to the published morphology of *T. epiphyllum* with perithecial ranges of 0.15–0.3 mm and 0.3–0.5 mm for setae. Phylogenetically, however, none of the mtSSU sequences cluster with the Neotropical *T. epiphyllum* lineage (Fig. 2). Instead, they are recovered in two distinct, well-supported subclades within the *Trichothelium* clade. This demonstrates that morphologically similar material occurs in multiple phylogenetic lineages and highlights the poor correspondence between phenotype and phylogeny within the clade. In the absence of sequences from type material, it remains impossible to determine whether the examined lineage represents *T. epiphyllum* s. str. or an undescribed species. Moderate genetic divergence is present within the Paleotropical lineage in both the mtSSU and ITS datasets, but no corresponding phenotypic differences could be identified. The taxon exhibited high photobiont selectivity and was exclusively associated with algal haplotype 33:1 (Figs 4, 5). Overall, pending type-linked sequences, these specimens are therefore retained as *T. cf. epiphyllum*.

##### Specimens examined.

MADAGASCAR – **Diego Suarez Prov**. • Antsiranana, Region: Diana, District: Diego II, Commune: Joffreville, Forontany: Morafeu, National Park of Montagne d’Ambre, along trail from Gîte des Roussettes, across stream at small dam to dirt road, moist but not wet lower montane forest, with tall trees and tree ferns, 12°31'55"S, 49°10'33"E, alt. 1119 m, 7 Oct. 2014; Ertz 20271F (BR, TAN). • ibid., across stream at small dam to dirt road and trail down to Lake Mahasarika, moist but not wet lower montane forest, with tall trees and tree ferns, 12°32'03"S, 49°10'28"E, alt. 1177 m, 7 Oct. 2014; Ertz 20272I (BR, TAN). • ibid., West of Sambava, Marojejy National Park, along trail from Camp Simpona down to Camp Marojejia, rather dense humid montane forest, 14°26'20"S, 49°45'08"E, alt. ca. 1200 m, 23 Oct. 2014; Ertz 20279F, 20279J, 20279K (BR, TAN). – **Toliara Prov**. • ca. 25 km Northwest of Fort-Dauphin (Tolagnaro), National Park of Andohahela, South part of parcel 1, ca. 5 km West of the village of Malio, 24°55'58"S, 46°44'07"E (ca. 200 m), 600–730 m, humid forest on the slopes of an inselberg, 29 Nov. 2019; Ertz 25340B (BR, TAN). • ibid., South part of parcel 1, ca. 6.5 km West of the village of Malio, 24°56'24.1"S, 46°43'26.2"E, alt. 1000 m, dense humid forest, 01 Dec. 2019; Ertz 25409B (BR, TAN).

#### Trichothelium

Taxon classificationFungiOstropalesPorinaceae

sp.

93261834-6359-5E97-9DA9-83CE461899C4

##### Notes.

Specimen 20279G had few perithecia, most of which had damaged or missing setae. Although the hymenia and ascospores were in good condition, the absence of sufficiently intact setae, an important diagnostic character in *Trichothelium*, prevented confident identification. The available morphological characters broadly resembled those of the specimens provisionally identified as *T. cf. epiphyllum*. However, the sequences of specimen 20279G did not group with those of the specimens in either the mtSSU or ITS phylogeny. The specimen was therefore retained as a separate, unidentified taxon rather than assigned to *T. cf. epiphyllum*. Its 7-septate ascospores measured (32–)32.5–37.2(–38) × (4.7–)5.3–6.3(–6.5) µm (n = 13).

##### Specimens examined.

MADAGASCAR – **Diego Suarez Prov**. • Antsiranana, West of Sambava, Marojejy National Park, along trail from Camp Simpona down to Camp Marojejia, rather dense humid montane forest, 14°26'20"S, 49°45'08"E, alt. ca. 1200 m, 23 Oct. 2014; Ertz 20279G (BR, TAN).

## Discussion

### Mycobiont diversity and phylogeny

This study revealed an unexpectedly high diversity of foliicolous *Porinaceae* in the southwestern Indian Ocean islands, documenting 37 taxa, including six new species, through an integrative taxonomic approach (Lücking et al. 2021). Molecular sampling of foliicolous *Porinaceae* was substantially expanded by generating mtSSU and ITS sequences for 114 specimens. Many of the recovered taxa had never been sequenced previously, whereas others were represented only by Neotropical material in existing molecular datasets, highlighting the limited taxonomic and geographic coverage of current phylogenetic frameworks. The new sequences were recovered across six major, well-supported clades, suggesting that the present-day diversity of *Porinaceae* in the study region originated through multiple independent colonisation events.

The distribution of taxa across the three islands showed pronounced geographic structuring: 32 of the 37 taxa were restricted to a single island, and none occurred on all three. This pattern is consistent with the high levels of island endemism reported for foliicolous lichens (Lücking et al. 2020; Lebreton et al. 2025). Interestingly, the 32 (86.5%) taxa restricted to a single island in the present study are comparable to the 84% of *Gomphillaceae* species restricted to a single territory reported by Lebreton et al. (2025). Despite this similarity, Lebreton et al. (2025) reported a Sørensen similarity of 0.18 between Madagascar and Mayotte, whereas the present study found no *Porinaceae* taxa shared between these islands. This discrepancy may reflect differences between the two families, as well as differences in sampling depth and taxonomic coverage. The greater diversity recorded in Madagascar is likely attributable to its larger land area, greater habitat heterogeneity, and more intensive sampling. Likewise, the absence of taxa shared among all three islands may reflect the limited long-distance dispersal capacity of foliicolous lichens, although incomplete sampling across the study region probably also contributes to this pattern (Lücking 2008; Baloch and Grube 2009).

As expected for foliicolous lichens, the limited number of informative morphological characters proved insufficient to accurately reflect phylogenetic relationships (Lücking 2008). Several morphologically similar taxa that were difficult to distinguish using phenotype alone were found to be non-monophyletic or to represent deeply divergent evolutionary lineages. These results emphasise the value of integrative taxonomy, combining morphological and molecular data for species delimitation in *Porinaceae*. More broadly, the phylogenetic analyses demonstrate that the family comprises several well-supported lineages that are only partly congruent with current generic circumscriptions. Although recent molecular studies have substantially improved our understanding of relationships within the family, many lineages remain poorly sampled, and their taxonomy requires further refinement (Baloch and Grube 2006; Sobreira et al. 2018; Ertz and Diederich 2022; Lebreton et al. 2024). Consequently, the following discussion is organised by major phylogenetic clades, providing a framework for interpreting the taxonomic and biogeographic patterns identified in this study.

**Figure 14.**
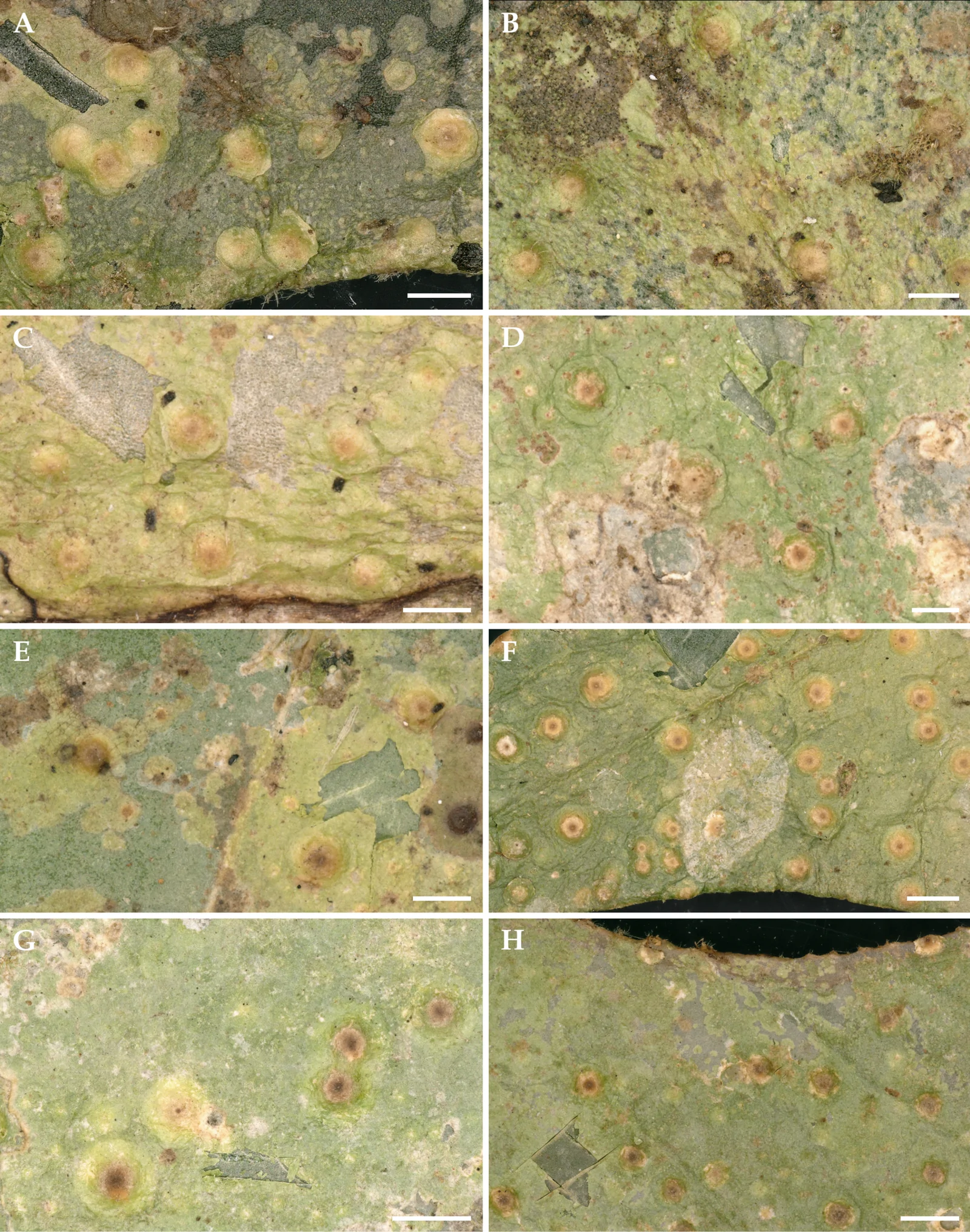
Specimens of the *Phylloporina* clade identified as or allied to *Porina epiphylla* and *P. lucida*. **A***P. cf. epiphylla* 1 (20274D, Madagascar) **B***P. cf. epiphylla* 2 (24006A, Mauritius) **C***P. aff. epiphylla* (22131H, Mayotte) **D***P. cf. lucida* 1 (20270H, Madagascar) **E***P. cf. lucida* 2 (21938B, Mayotte) **F***P. aff. lucida* 1 (20271C, Madagascar) **G***P. aff. lucida* 2 (20280H, Madagascar) **H***P. aff. lucida* 3 (20276F, Madagascar). Scale bars: 0.5 mm (**A**–**E**); 1 mm (**F**–**H**).

**Figure 15.**
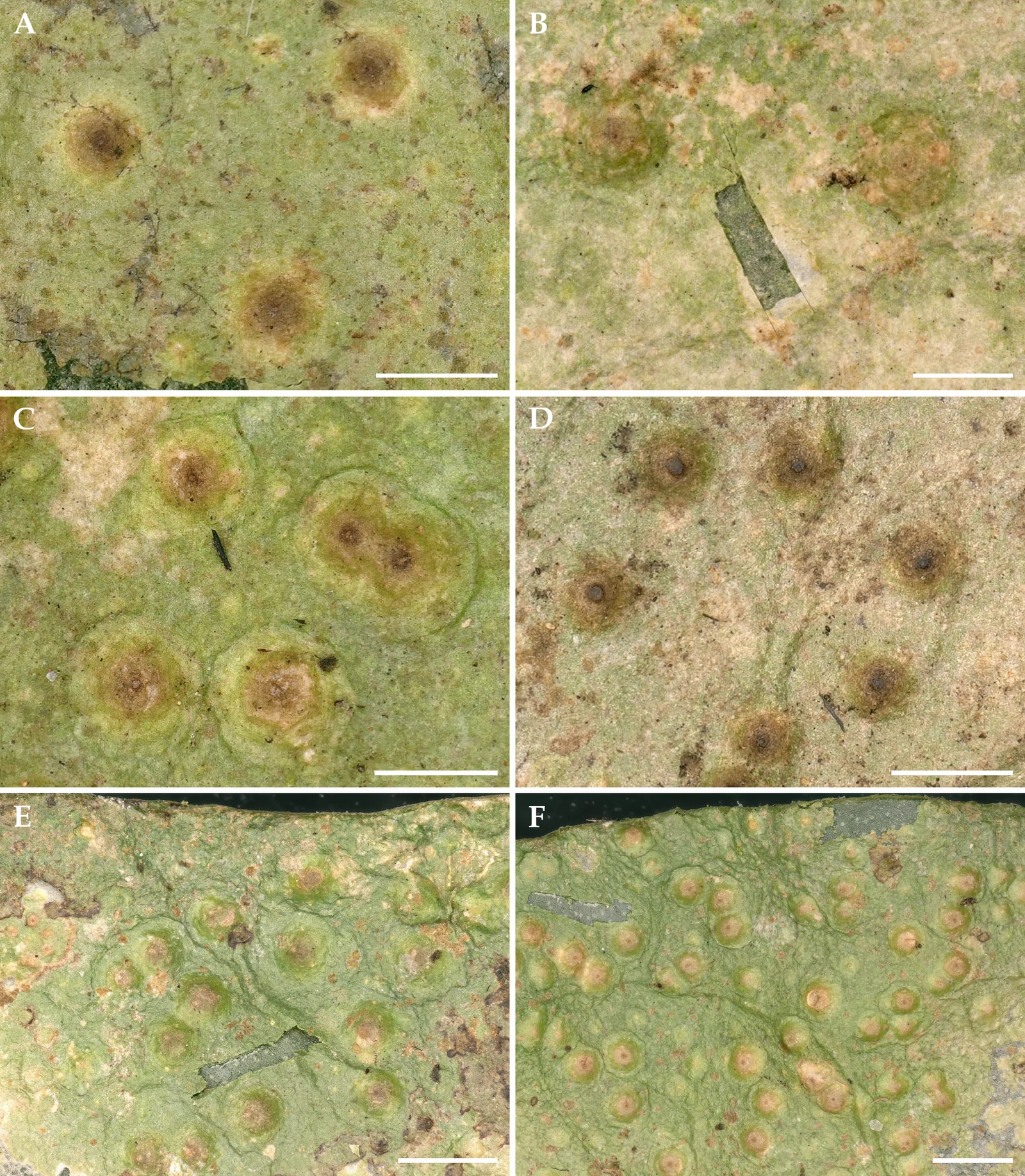
Selected specimens representing the *Phylloporina* clade, excluding *P. epiphylla*, *P. lucida*, and newly described species. **A***P. cf. atriceps* (21856B, Mayotte) **B***P. cf. australiensis* (25303H, Madagascar) **C***P. cf. karnatakensis* 1 (22135B, Mayotte) **D***P. cf. karnatakensis* 2 (25303G, Madagascar) **E***P. aff. subepiphylla* 1 (20272E, Madagascar) **F***P. aff. subepiphylla* 2 (20272D, Madagascar). Scale bars: 0.5 mm (**A**–**D**); 1 mm (**E, F**).

***Phylloporina* clade**. (Figs 9, 10, 14, 15) This clade represents the most species-rich and genetically diverse lineage recovered in the present study, including two newly described species, *Porina complanata* (Fig. 9) and *P. madagascariensis* (Fig. 10). Although the backbone of the clade is well supported, relationships among terminal lineages remain poorly resolved in both the mtSSU and ITS phylogenies, highlighting the difficulty of delimiting taxa within this group, particularly among the currently sequenced material. Moreover, several accessions identified as *P. karnatakensis*, *P. atriceps*, and *P. mastoidella* fall outside their expected phylogenetic positions, suggesting that their identifications should be treated with caution.

The mtSSU phylogeny recovers distinct Paleotropical and Neotropical subclades associated with *P. epiphylla* and *P. lucida*, each comprising specimens identified as or morphologically allied to these taxa (Fig. 14). These subclades reveal a marked incongruence between phenotype and phylogeny, as neither taxon forms a well-supported monophyletic lineage. Notably, *P. aff. lucida* 3 (Fig. 14H) and *P. aff. epiphylla* (Fig. 14C) are morphologically well differentiated despite exhibiting only little mtSSU sequence divergence. This pattern suggests that the current circumscriptions of *P. epiphylla* and *P. lucida* likely encompass geographically structured species complexes rather than single pantropical species. In the absence of sequenced data from type material, however, it remains impossible to determine whether the Neotropical and Paleotropical subclades represent geographically differentiated populations of broadly circumscribed species or multiple independently evolving taxa. Until additional molecular data, particularly from type specimens or type localities, become available, the most conservative interpretation is that these subclades represent geographically structured entities with overlapping phenotypes.

This pattern is consistent with the findings of Baloch and Grube (2009), who reported extensive cryptic diversity and substantial mtSSU divergence within the *Phylloporina* clade. In the present study, this is reflected by relatively high levels of genetic divergence associated with limited morphological differentiation, with many taxa sharing predominantly 7-septate ascospores that differ only subtly in size. Similarly, some taxa exhibit pronounced phenotypic differences despite showing little genetic divergence. Phenotypic convergence further complicates species delimitation and may lead to erroneous identifications; for example, the warted perithecial morphology characteristic of *P. subepiphylla*-like specimens (Fig. 15E, F) appears to have evolved independently in more than one major lineage. Species delimitation is particularly challenging in *Porinaceae* because the relatively small number of informative morphological characters makes it difficult to identify clear discontinuities that support robust species concepts. This emphasises the importance of integrating molecular evidence, especially sequence data from type material, into taxonomy studies (Lücking 2008; Lücking et al. 2021). Overall, our results reveal substantial hidden diversity within the *Phylloporina* group and indicate that current species concepts considerably underestimate its evolutionary diversity.

**Figure 16.**
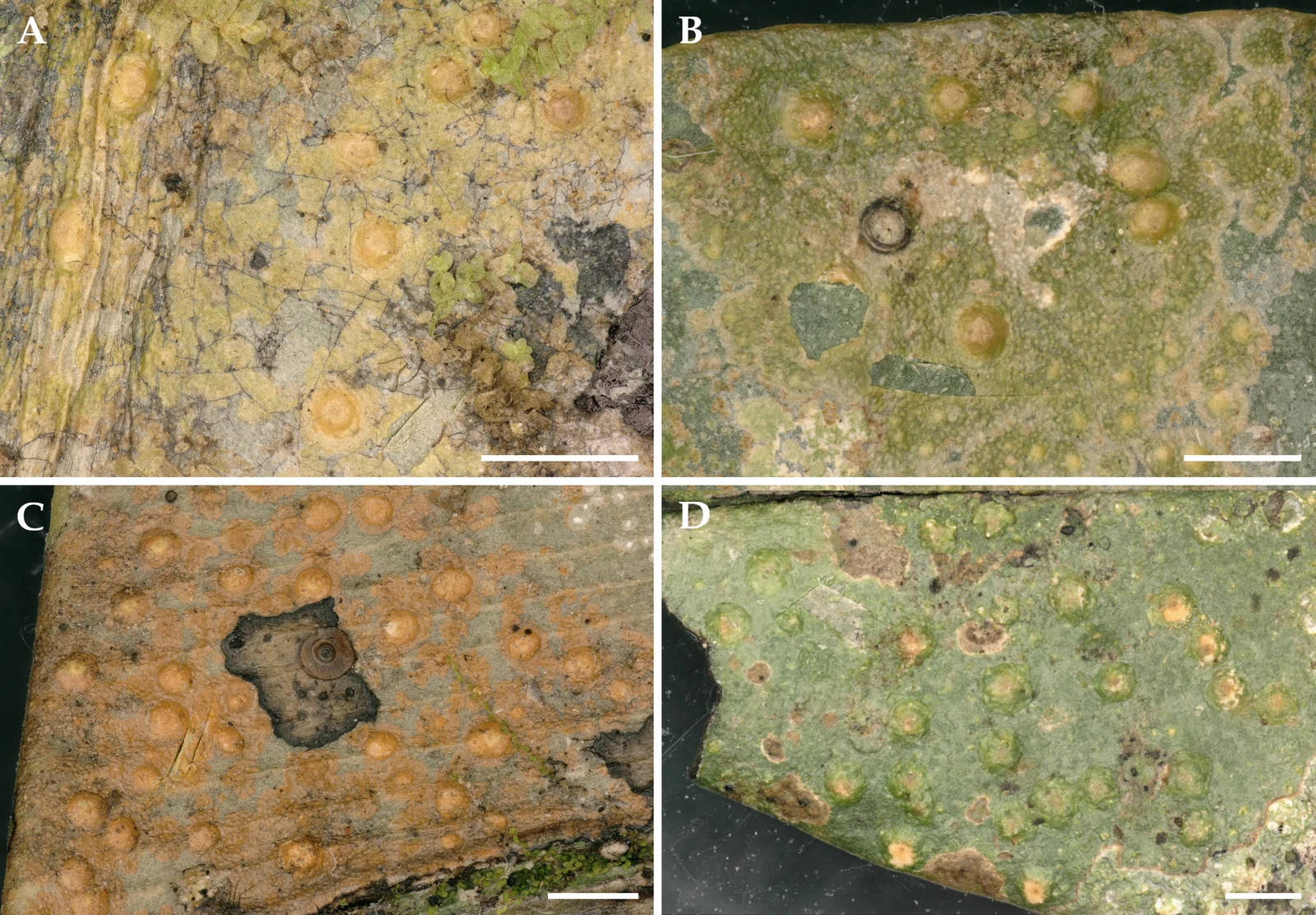
Selected specimens representing the *Porina* s. str. clade, excluding newly described species. **A***P. cf. epiphylloides* (24305H, Mauritius) **B***P. mazosioides* (20270J, Madagascar) **C***P. mazosioides* (23981A, Mauritius), fresh specimen **D***P. aff. mazosioides* (20280G, Madagascar). Scale bars: 1 mm (**A**–**D**).

***Porina* s. str. clade**. (Figs 11, 12, 16) This clade, sister to *Phylloporina*, shows a well-resolved structure in both the ITS and mtSSU phylogenies, and includes two newly described species. The backbone of the clade is strongly supported, and most terminal relationships are well resolved, with the exception of the predominantly Neotropical subclade, where several internal relationships remain poorly supported. The inclusion of both foliicolous and corticolous taxa, which do not form separate monophyletic groups, indicates that substrate preference is not phylogenetically conserved and suggests that shifts between substrates have likely occurred repeatedly during the evolutionary history of the clade. The Paleotropical subclade also exhibits pronounced phylogeographic structuring. Several taxa are shared between Madagascar and Mauritius, whereas Mayotte is represented by a single, unshared taxon, *Porina mayottensis* (Fig. 11). Although this pattern is consistent with island endemism, additional sampling will be required to determine whether its apparent restriction to Mayotte reflects a genuinely narrow distribution or incomplete sampling.

In contrast to the *Phylloporina* clade, species of *Porina* s. str. are generally well delimited, with phylogenetic relationships largely congruent with phenotypic species boundaries. Nevertheless, a few taxa reveal discordance between phenotype and phylogeny. For example, *P. longispora* and *P. aff. mazosioides* (Fig. 16D) differ markedly in ascospore septation despite exhibiting only limited mtSSU sequence divergence, suggesting that ascospore septation may be evolutionarily more labile than the molecular data imply. Additionally, both taxa differ in photobiont morphotype, (see below). Similarly, *P. epiphylloides* and *P. cf. epiphylloides* (Fig. 16A) differ in ascospore length from the original species description, despite showing low mtSSU divergence. Altogether, these cases suggest that some morphological characters traditionally regarded as taxonomically informative may exhibit greater intraspecific variability than previously recognised or may be influenced by atypical specimens. These findings further emphasise the importance of comprehensive phenotypic and molecular sampling, particularly of type material, for establishing robust species boundaries.

**Figure 17.**
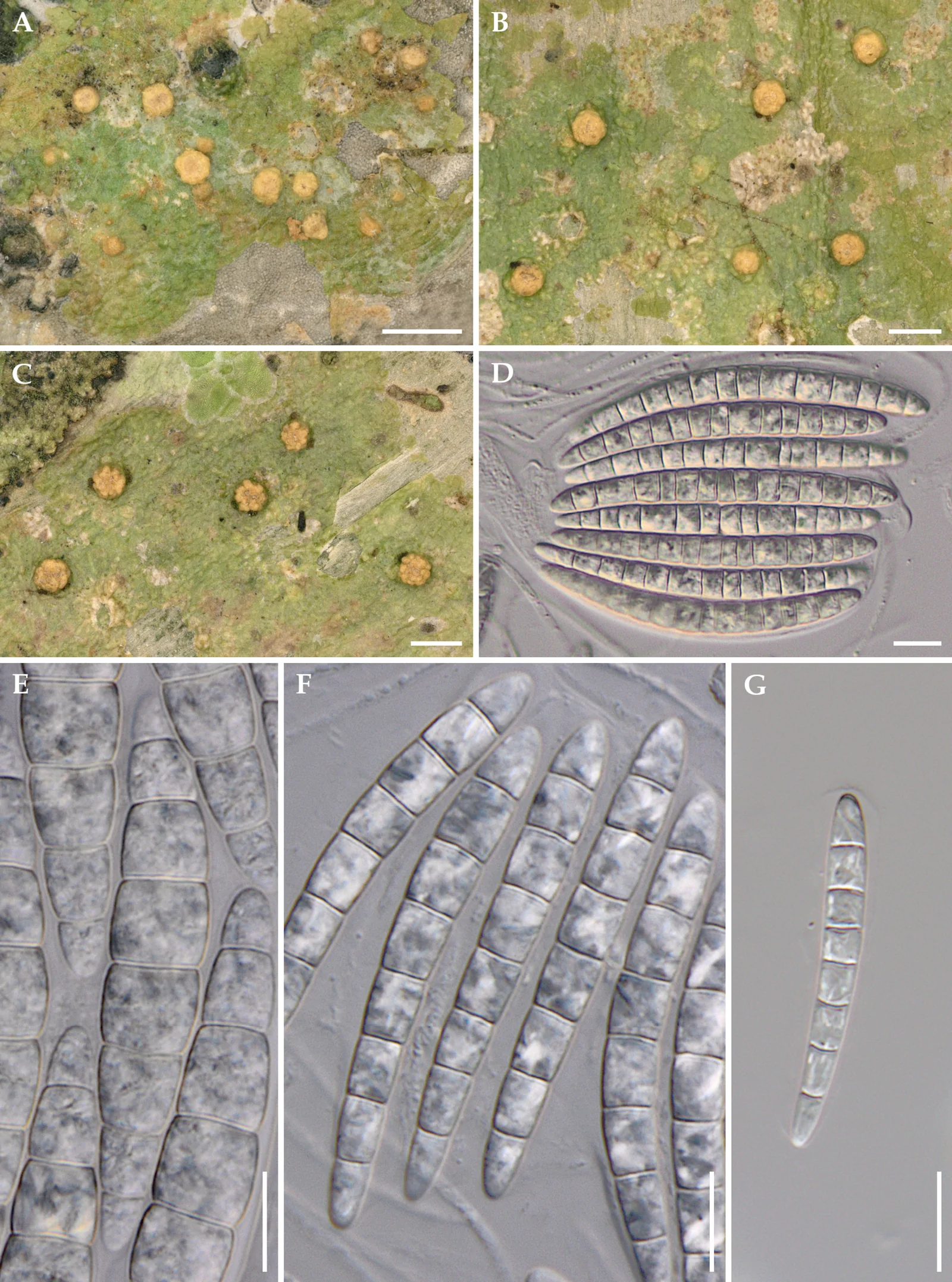
Selected specimens representing the *Porina sphaerocephala* clade and ascospore variation within. **A***P. sphaerocephala* (25411C, Madagascar) **B***P. sphaerocephaloides* (20279L, Madagascar), standard morphology observed **C***P. sphaerocephaloides* (20279M, Madagascar), divergent specimen with more verrucose perithecia **D** 13–15–septate ascospores in water, from divergent *P. sphaerocephaloides* specimen (20279M, Madagascar) **E** Wider and slightly constricted ascospores in water, from *P. sphaerocephaloides* specimen (20279L, Madagascar) **F** Narrower ascospores in water, from *P. sphaerocephaloides* specimen (25375G, Madagascar) **G** Ascospores in water, from *P. sphaerocephala* specimen (25321D, Madagascar). Scale bars: 0.5 mm (**A**–**C**); 10 µm (**D**–**G**).

***Porina sphaerocephala* clade**. (Fig. 17) This clade, sister to the *Saxiloba* clade, includes the recently described corticolous *P. nuculoides* and saxicolous *P. rupicola*, together with the newly sequenced foliicolous species *P. sphaerocephala* (Fig. 17A) and *P. sphaerocephaloides* (Fig. 17B, C), representing the first foliicolous members of this lineage (Ertz and Diederich 2022). Both foliicolous species were previously assigned to the *P. radiata* group by Lücking (2004), together with other taxa characterised by a verrucose-rugose thalli, rectangular photobiont cells, and a crystallostratum, such as *P. mazosioides* (Fig. 16B, C) and *P. radiata* Kalb, Lücking & Vězda. The occurrence of foliicolous, corticolous, and saxicolous species within the same lineage indicates that substrate preference is not phylogenetically conserved and that shifts between substrates have likely occurred repeatedly during the evolution of the clade. The isolated Neotropical accession, identified as *P. internigrans* (Nyl.) Müll. Arg., should be interpreted cautiously, as other sequenced representatives of this species are recovered in a distantly related lineage, suggesting a probable misidentification or contamination. The examined specimens of *P. sphaerocephala* were relatively homogeneous, exhibiting both limited genetic divergence and highly consistent morphology across the ITS and the mtSSU phylogenies.

*Porina sphaerocephaloides*, however, displayed a markedly different pattern. Although its external morphology remained comparatively uniform, it exhibited substantial genetic divergence together with pronounced variation in ascospore morphology (Fig. 17D–F), supporting its interpretation as a species complex. Ascospore septation ranged predominantly from 7 to 12 septa and was accompanied by considerable variation in ascospore dimensions. One specimen (20279M) was particularly divergent, with 13–15-septate ascospores and slightly more verrucose perithecia (Fig. 17C, D). Nevertheless, because it remains phylogenetically nested within the broader *P. sphaerocephaloides* complex, it is not recognised here as a separate taxon. Although both the ITS and mtSSU phylogenies recover several supported subgroups, these do not correspond to monophyletic groups characterised by consistent morphological differences. Consequently, neither ascospore morphology nor external phenotype reliably reflects the underlying phylogenetic structure within this complex. Overall, the *P. sphaerocephala* clade illustrates the limitations of relying on ecological or morphological characters alone for species delimitation in foliicolous *Porinaceae*.

**Figure 18.**
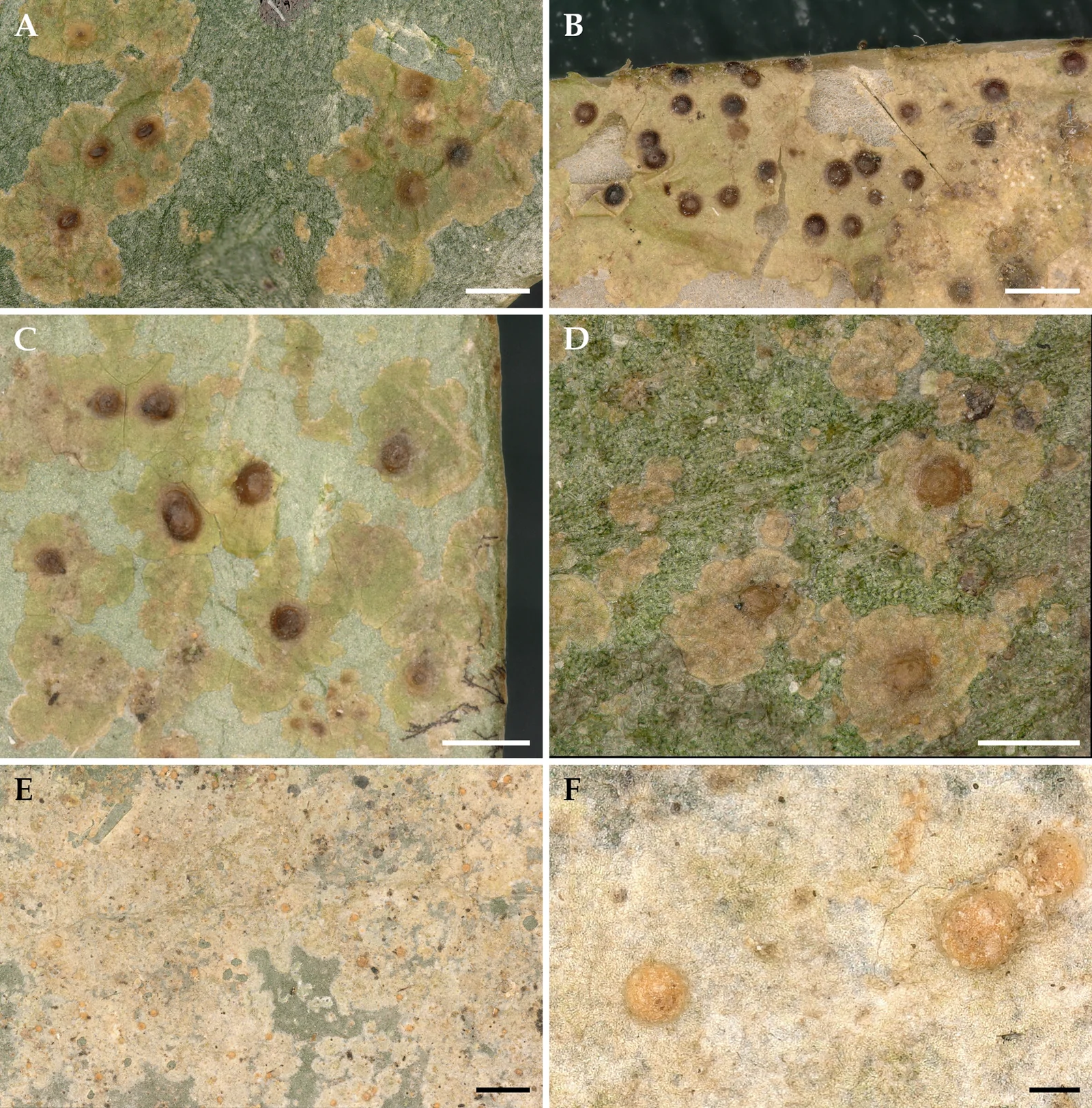
Selected specimens representing the *Phragmopeltheca* and *Porina fusca* clade. **A***P. limbulata* (20271I, Madagascar) **B***P. rubentior* (25411G, Madagascar) **C***P. rufula* (21938A, Mayotte) **D***P. rufula* (23539D, Mauritius) **E***P. cf. leptosperma* (20274C, Madagascar), view of thallus with perithecia **F***P. cf. leptosperma* (20274C, Madagascar), close-up of perithecia. Scale bars: 0.5 mm (**A**–**D**); 1 mm (**E**); 0.1 mm (**F**).

***Phragmopeltheca* clade**. (Fig. 18A–D) This well-supported clade is now shown to include not only foliicolous taxa but also a bambusicolous species based on ITS data. Although bambusicolous and foliicolous habitats are ecologically similar, the bambusicolous specimen is treated here as a distinct substrate, indicating that substrate preference is not strictly conserved within the clade. The expanded mtSSU phylogeny displays *Porina limbulata* (Fig. 18A) as paraphyletic, supporting the hypothesis of Baloch and Grube (2006) that it represents a species complex. Within this assemblage, Paleotropical and Neotropical specimens form two well-supported subgroups in the mtSSU phylogeny, indicating pronounced phylogeographic structuring. Nested within the *P. limbulata* complex is *P. rubentior* (Fig. 18B), which showed exceptionally low mtSSU divergence between Paleotropical and Neotropical specimens, a pattern that contrasts with the strong geographic differentiation observed elsewhere in the *Porinaceae* phylogeny. A possible explanation is that its comparatively small ascospores facilitate more effective long-distance dispersal, although this hypothesis remains speculative. Alternatively, the observed pattern may reflect recent divergence, incomplete geographic sampling, or the limited resolving power of the mtSSU marker.

A comparable pattern of geographic structuring is observed in *P. rufula* (Fig. 18C, D), which is likewise divided into well-supported Paleotropical and Neotropical lineages. However, the long internal branches recovered in the mtSSU phylogeny suggest that this taxon may harbour substantial cryptic diversity. Within the Paleotropical lineage, the specimens from Mayotte (Fig. 18C) form a distinct African subgroup, whereas the Mauritian specimen (Fig. 18D) groups with a Japanese accession (LC913215; Miyazawa and Ohmura 2026), suggesting an eastern Paleotropical affinity. A third subgroup, represented exclusively by eastern Paleotropical accessions, further suggests a complex biogeographic structure within the species. The ITS phylogeny is broadly congruent with the mtSSU phylogeny for the *Phragmopeltheca* clade but provides weaker support for the *P. limbulata-rubentior* complex. However, the ITS dataset does not include Neotropical or eastern Paleotropical representatives, highlighting the need for further sampling. Overall, this clade illustrates the combined effects of repeated substrate shifts, pronounced geographic structuring, and lineage-specific patterns of molecular divergence. The contrasting evolutionary histories of *P. limbulata*, *P. rubentior*, and *P. rufula* further demonstrate that closely related taxa can differ markedly in their levels of genetic differentiation and geographic structure, highlighting the complex evolutionary dynamics that underlie species diversification in *Porinaceae*.

***Porina fusca* clade**. (Fig. 18E, F) This relatively underrepresented clade is recovered only in the mtSSU phylogeny, limiting taxonomic conclusions and emphasising the need for additional sampling. The unstable placements of several *P. leptalea* (Durieu & Mont.) A.L. Sm. accessions suggest that some identifications may be unreliable. Furthermore, the examined specimen of *P. cf. leptosperma* is morphologically notable. Although slightly smoother, its perithecia show a striking resemblance to those of *P. sphaerocephala* in shape and colour (Fig. 17A). This observation suggests convergence in external morphology, illustrating the limited taxonomic value of external resemblance alone.

**Figure 19.**
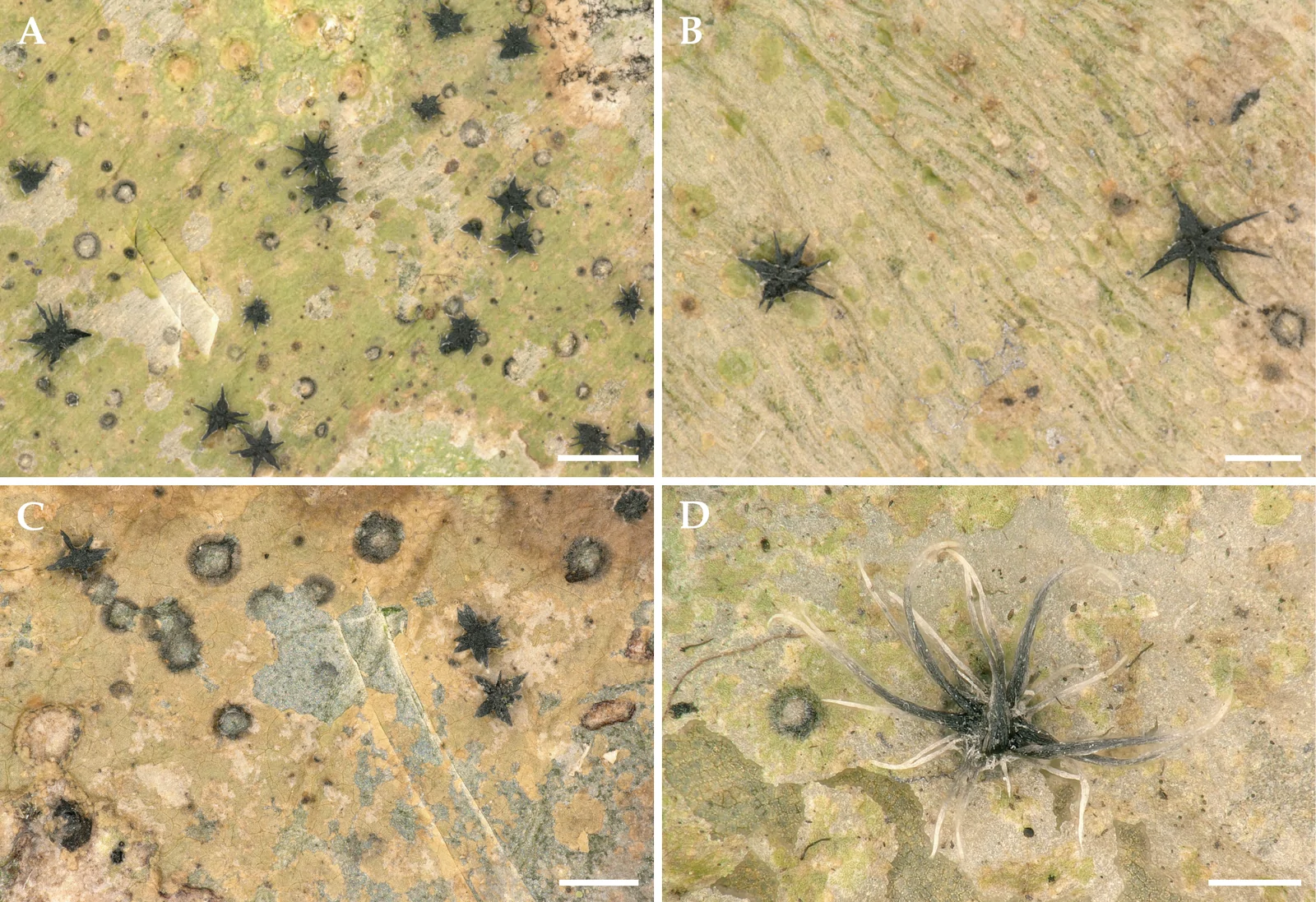
Selected specimens representing the *Trichothelium* clade, excluding newly described *Porina* species. **A***T. cf. epiphyllum* (20279K, Madagascar) **B***T. cf. epiphyllum* (20279F, Madagascar) **C***T. cf. epiphyllum* (20271F, Madagascar) **D***T. aff. argenteum* (20277C, Madagascar). Scale bars: 1 mm (**A**); 0.5 mm (**B, C**); 0.25 mm (**D**).

***Trichothelium* clade**. (Figs 8, 13, 19) This clade includes taxa currently assigned to both *Porina* and *Trichothelium*, representing a prime example of the challenges associated with generic delimitation. Although the clade itself is well–supported, its internal structure is largely unresolved, with only a few relatively homogeneous subclades recovered. In this clade, commonly used phenotypic characters for generic and specific delimitation, such as setae and perithecial wall pigmentation (K-/K+), show little correspondence with the recovered phylogeny, suggesting that these features are evolutionarily labile and potentially homoplastic. Furthermore, the mtSSU phylogeny indicates that the genus *Trichothelium* is not monophyletic. Specimens identified as *T. epiphyllum* (Fig. 19A–C) form a distinct Paleotropical subclade that is separated from a Neotropical subclade containing specimens identified as the same species, as well as accessions assigned to *Porina borreri* (Trevis.). D. Hawksw. & P. James. This pattern suggests that the current circumscription of *T. epiphyllum* may be too broad or may conceal multiple geographically structured lineages. In the absence of molecular data from type material, the currently sequenced specimens are referred to as *T. cf. epiphyllum*. Moreover, *Porina nitidula* Müll. Arg. is recovered in multiple mtSSU subclades together with other taxa, suggesting either that the species is non-monophyletic or that some published identifications are incorrect. Resolving these uncertainties will require molecular data generated from type or otherwise reliably authenticated material.

The observed incongruence between morphology and phylogeny is further illustrated by characters traditionally regarded as diagnostic at the generic level. Most species within the *Trichothelium* clade possess black, K– perithecial walls, yet the newly described *Porina cincta* (Fig. 8) exhibits red, K+ perithecia while remaining nested within the same lineage. This suggests that perithecial pigmentation alone is an unreliable character for delimiting natural groups within the clade. Similarly, the newly described *P. trichocoronata* (Fig. 13) displays white hyphal hairs that, in some perithecia, fuse to form setae-like structures, representing an apparent morphological intermediate between hyphal hairs and true setae. These observations support earlier suggestions by Baloch and Grube (2006) that several characters used for taxonomic delimitation vary gradually across *Porinaceae*. Overall, the results suggest that current generic circumscriptions within the *Trichothelium* clade require re-evaluation, as several traditionally diagnostic characters appear to be evolutionarily labile.

### Photobiont diversity and selectivity

Although trentepohlioid photobionts are common among lichens, their diversity within foliicolous *Porinaceae* remains largely unknown. The *rbc*L phylogeny revealed unexpectedly high diversity, with 25 algal haplotypes distributed across six major clades, four of which are newly recovered. Despite only two broad morphotypes being recognised, angular-rounded (irregularly arranged) and rectangular (arranged in radiate plates) algal cells, the recovered phylogeny reveals substantial cryptic diversity among the photobionts (Lücking and Vězda 1998; Baloch and Grube 2006, 2009; Lücking 2008). Cell morphology alone is therefore insufficient to infer photobiont lineages, as the two morphotypes occur across several distinct clades. No consistent haplotype-specific differences in cell measurements, shape, and interspace were observed, except for rectangular haplotypes 33:4 (Fig. 6G) and 33:5 (Fig. 6H), which appeared to slightly differ in cell dimensions.

Within *Phylloporina*, all sequenced taxa formed reciprocal and exclusive symbiotic associations with angular-rounded algae from clade 26 (Fig. 6A, B). This pattern suggests strong photobiont specificity and indicates that photobiont *rbc*L data may provide useful complementary evidence for recognising major evolutionary lineages within the mycobionts. In this context, photobiont clade 26 appears to be a useful indicator of the *Phylloporina* clade, although it should not be used alone for taxonomic purposes. Notably, clade 26 exhibited the highest haplotype diversity and the broadest range of symbiotic associations, with several algal haplotypes shared among multiple fungal taxa. Conversely, *P. cf. epiphylla* 1, *P. aff. lucida* 2, and *P. aff. subepiphylla* 2, each harboured two distinct algal haplotypes within a single thallus, suggesting that individual mycobionts can associate simultaneously with multiple closely related photobiont lineages. Although thallus sections were screened for visible free-living or epiphytic algae and other extraneous material before DNA sampling, the inadvertent inclusion of non-symbiotic algae cannot be entirely ruled out. Confirmation that multiple photobiont lineages genuinely co-occur within a single thallus would require approaches such as detailed microscopic observations showing direct associations between the mycobiont and the two different photobionts.

Overall, symbiotic patterns outside the *Phylloporina* clade appear more variable, with some fungal taxa forming associations with multiple photobiont clades and vice versa. Furthermore, the results suggest that photobiont morphotypes are less stable across the rest of the *Porinaceae*. The presence or absence of calcium oxalate crystals in the thallus does not appear to be linked to photobiont identity, since haplotype 33:1 (Fig. 6E, F) was recovered from species with both crystalline and non-crystalline thalli. Cases such as the marked differences in algal morphologies observed between *P. longispora* and *P. aff. mazosioides* (Fig. 7C, D) suggest that photobiont morphotypes are not always reliable taxonomic proxies, even when genetic divergence is minimal. Examples such as *P. mayottensis* further complicate photobiont identification, as it was the only taxon examined that harboured several algal morphotypes in most examined specimens (Fig. 7E, F). However, it remains unclear whether this represents a single flexible photobiont taxon or multiple co-occurring lineages. Altogether, the recovered algal diversity reveals an additional layer of hidden diversity within foliicolous *Porinaceae*. The results show both high specificity in some lineages and substantial flexibility in others. The apparent differences in photobiont specificity and flexibility should nevertheless be interpreted with caution, as taxa represented by more specimens had greater opportunities to detect multiple photobiont associations than sparsely sampled taxa did. Therefore, the data support a more nuanced taxonomic view in which photobiont identity may be informative in some clades but less so in others. This study provides a first contribution towards addressing the limited knowledge of photobiont diversity in foliicolous lichens and highlights the need for denser, population-level sampling of photobionts.

## Conclusion

This study revealed that foliicolous *Porinaceae* within MIOI are much more diverse than previously expected, indicating that the region has been undersampled. It includes six newly described species and multiple taxa that were previously unsequenced or lacked Paleotropical representatives. Nevertheless, this diversity is only partly captured by current phenotypic and phylogenetic concepts. Strong phylogeographic structuring was recovered in several sampled clades. This was often paired with substantial cryptic diversity, and traditionally used diagnostic characters, such as ascospore septation, were less reliable than expected for reflecting phylogenetic relationships. Furthermore, some generic boundaries, such as for *Trichothelium*, appear unclear and cannot be defined accurately using isolated characters such as morphology and substrate preference. Overall, patterns recovered across the phylogeny, phenotype, and geography were only partially congruent, revealing that current species and genus concepts remain too unrefined in parts of the family. These results confirm that an integrative taxonomical approach combining robust phenotypic assessment with multi-locus phylogenetic data, including type-linked material, is essential for reliable taxonomic delimitation within *Porinaceae*.

Cryptic diversity is not restricted to mycobionts but also extends to photobionts. The *rbc*L marker revealed substantial algal diversity largely obscured by only two broadly described morphotypes. Some fungal lineages, such as *Phylloporina*, exhibited strong photobiont specificity, whereas others appeared less selective. Overall, the data indicate unexpectedly broad variation in symbiotic association patterns across *Porinaceae*, which is not reflected by observable morphology. These results show that photobiont identity can be taxonomically valuable in some lineages, but should be interpreted with caution overall and always in combination with molecular data.

Altogether, this study emphasises the need for extensive multi-locus and type-linked sampling, combined with thorough phenotypic assessments, to refine species and genus concepts within *Porinaceae*. Lineage-specific revisions across broader geographical ranges will be necessary to resolve persistent taxonomic uncertainty and to understand the evolutionary patterns observed within the family.

## Supplementary Material

XML Treatment for Porina cf. atriceps

XML Treatment for Porina cf. australiensis

XML Treatment for Porina cincta

XML Treatment for Porina complanata

XML Treatment for Porina aff. epiphylla

XML Treatment for Porina cf. epiphylloides

XML Treatment for Porina cf. leptosperma

XML Treatment for Porina limbulata

XML Treatment for Porina madagascariensis

XML Treatment for Porina mayottensis

XML Treatment for Porina mazosioides

XML Treatment for Porina aff. mazosioides

XML Treatment for Porina parva

XML Treatment for Porina rubentior

XML Treatment for Porina rufula

XML Treatment for Porina sphaerocephala

XML Treatment for Porina sphaerocephaloides

XML Treatment for Porina trichocoronata

XML Treatment for Trichothelium aff. argenteum

XML Treatment for Trichothelium cf. epiphyllum

XML Treatment for Trichothelium

## Additional information

### Artificial Intelligence (AI) use

The authors accept full responsibility for the content of the manuscript, including the disclosure of any use of AI.

Regarding the use of AI in the preparation of this manuscript, the authors declare the following: GPT.

**Used for**: Language, style and writing.

To improve the English of some parts of the text.

### Adherence to national and international regulations

Specimen collection was conducted in accordance with the Nagoya Protocol, where applicable.

### Author ORCIDs

S. Debaene https://orcid.org/0009-0001-3469-2426

L. Borgato https://orcid.org/0000-0001-5120-7253

D. Ertz https://orcid.org/0000-0001-8746-3187

### Data availability

The DNA sequences generated in this study were deposited in NCBI (GenBank) at https://www.ncbi.nlm.nih.gov/genbank/. Additional figures and tables are provided as Supplementary Information.

## Supplementary materials

Supplementary material 1 Species names, voucher specimens and GenBank Accession numbers of mtSSU and ITS sequences used in this study Data type docx https://binary.pensoft.net/file/1829011This dataset is made available under the Open Database License (http://opendatacommons.org/licenses/odbl/1.0/). The Open Database License (ODbL) is a license agreement intended to allow users to freely share, modify, and use this Dataset while maintaining this same freedom for others, provided that the original source and author(s) are credited. Sandro Debaene, Damien Ertz

Supplementary material 2 Species names, voucher specimens and GenBank Accession numbers of *rbc*L sequences used in this study Data type docx https://binary.pensoft.net/file/1829012This dataset is made available under the Open Database License (http://opendatacommons.org/licenses/odbl/1.0/). The Open Database License (ODbL) is a license agreement intended to allow users to freely share, modify, and use this Dataset while maintaining this same freedom for others, provided that the original source and author(s) are credited. Sandro Debaene, Luca Borgato

Supplementary material 3 Complete mtSSU phylogenetic tree of the *Porinaceae* in PDF format Data type pdf https://binary.pensoft.net/file/1829013This dataset is made available under the Open Database License (http://opendatacommons.org/licenses/odbl/1.0/). The Open Database License (ODbL) is a license agreement intended to allow users to freely share, modify, and use this Dataset while maintaining this same freedom for others, provided that the original source and author(s) are credited. Sandro Debaene, Luca Borgato

Supplementary material 4 Complete ITS phylogenetic tree of the *Porinaceae* in PDF format Data type pdf https://binary.pensoft.net/file/1829014This dataset is made available under the Open Database License (http://opendatacommons.org/licenses/odbl/1.0/). The Open Database License (ODbL) is a license agreement intended to allow users to freely share, modify, and use this Dataset while maintaining this same freedom for others, provided that the original source and author(s) are credited. Sandro Debaene, Luca Borgato

Supplementary material 5 Complete *rbc*L phylogenetic tree of the *Trentepohliaceae* in PDF format Data type pdf https://binary.pensoft.net/file/1829015This dataset is made available under the Open Database License (http://opendatacommons.org/licenses/odbl/1.0/). The Open Database License (ODbL) is a license agreement intended to allow users to freely share, modify, and use this Dataset while maintaining this same freedom for others, provided that the original source and author(s) are credited. Sandro Debaene, Luca Borgato

Supplementary material 6 Nexus file containing the trimmed mtSSU alignment used in this study Data type nex https://binary.pensoft.net/file/1829016This dataset is made available under the Open Database License (http://opendatacommons.org/licenses/odbl/1.0/). The Open Database License (ODbL) is a license agreement intended to allow users to freely share, modify, and use this Dataset while maintaining this same freedom for others, provided that the original source and author(s) are credited.Author: Sandro Debaene

Supplementary material 7 Nexus file containing the trimmed ITS alignment used in this study Data type nex https://binary.pensoft.net/file/1829017This dataset is made available under the Open Database License (http://opendatacommons.org/licenses/odbl/1.0/). The Open Database License (ODbL) is a license agreement intended to allow users to freely share, modify, and use this Dataset while maintaining this same freedom for others, provided that the original source and author(s) are credited.Author: Sandro Debaene

Supplementary material 8 Nexus file containing the trimmed *rbc*L alignment used in this study Data type nex https://binary.pensoft.net/file/1829018This dataset is made available under the Open Database License (http://opendatacommons.org/licenses/odbl/1.0/). The Open Database License (ODbL) is a license agreement intended to allow users to freely share, modify, and use this Dataset while maintaining this same freedom for others, provided that the original source and author(s) are credited.Author: Sandro Debaene
