# Supplementary material for: Integrative taxonomy reveals hidden fungal and algal diversity in foliicolous Porinaceae (Ascomycota, Ostropales) from Madagascar and the Indian Ocean Islands
Source: IMA Fungus. 2026 Nov 5;17:e207656. doi: 10.3897/imafungus.17.207656 (PMC13760347; doi:10.3897/imafungus.17.207656)
Supplement: Supplementary file 1 [file imafungus-17-e207656-s003.pdf]

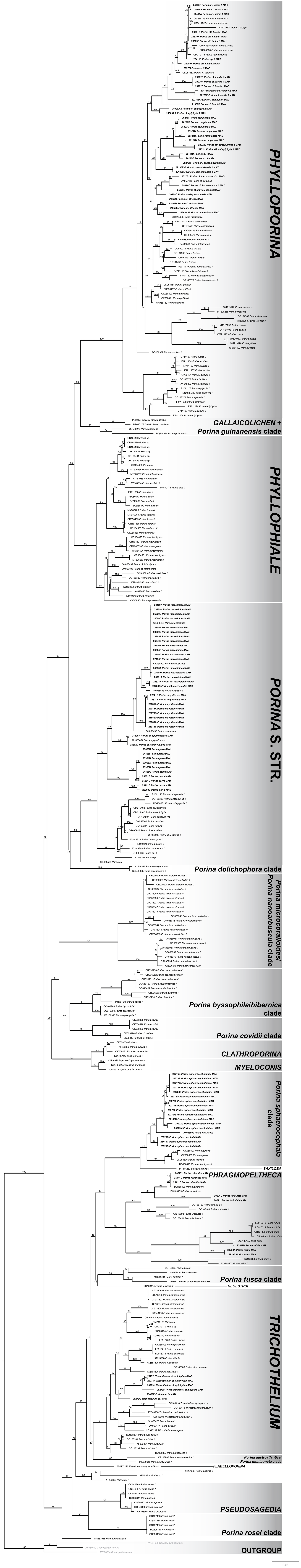
PHYLLOPORINA
GALLAICOLICHEN + Porina guinanensis clade
PHYLLOPHIALE
PORINA S. STR.
Porina dolichophora clade
Porina microcoralloides/ Porina nanoarbuscula clade
Porina byssophila/hibernica clade
Porina covidii clade
CLATHROPORINA
MYELOCONIS
Porina sphaerocephala clade
SAXILOBA
PHRAGMOPELTHECA
Porina fusca clade
SEGESTRIA
TRICHOTHELIUM
Porina austroatlantica/ Porina multipuncta clade
FLABELLOPORINA
PSEUDOSAGEDIA
Porina rosei clade
OUTGROUP
0.06
