# Supplementary material for: Integrative taxonomy reveals hidden fungal and algal diversity in foliicolous Porinaceae (Ascomycota, Ostropales) from Madagascar and the Indian Ocean Islands
Source: IMA Fungus. 2026 Nov 5;17:e207656. doi: 10.3897/imafungus.17.207656 (PMC13760347; doi:10.3897/imafungus.17.207656)
Supplement: Supplementary file 2 [file imafungus-17-e207656-s004.pdf]

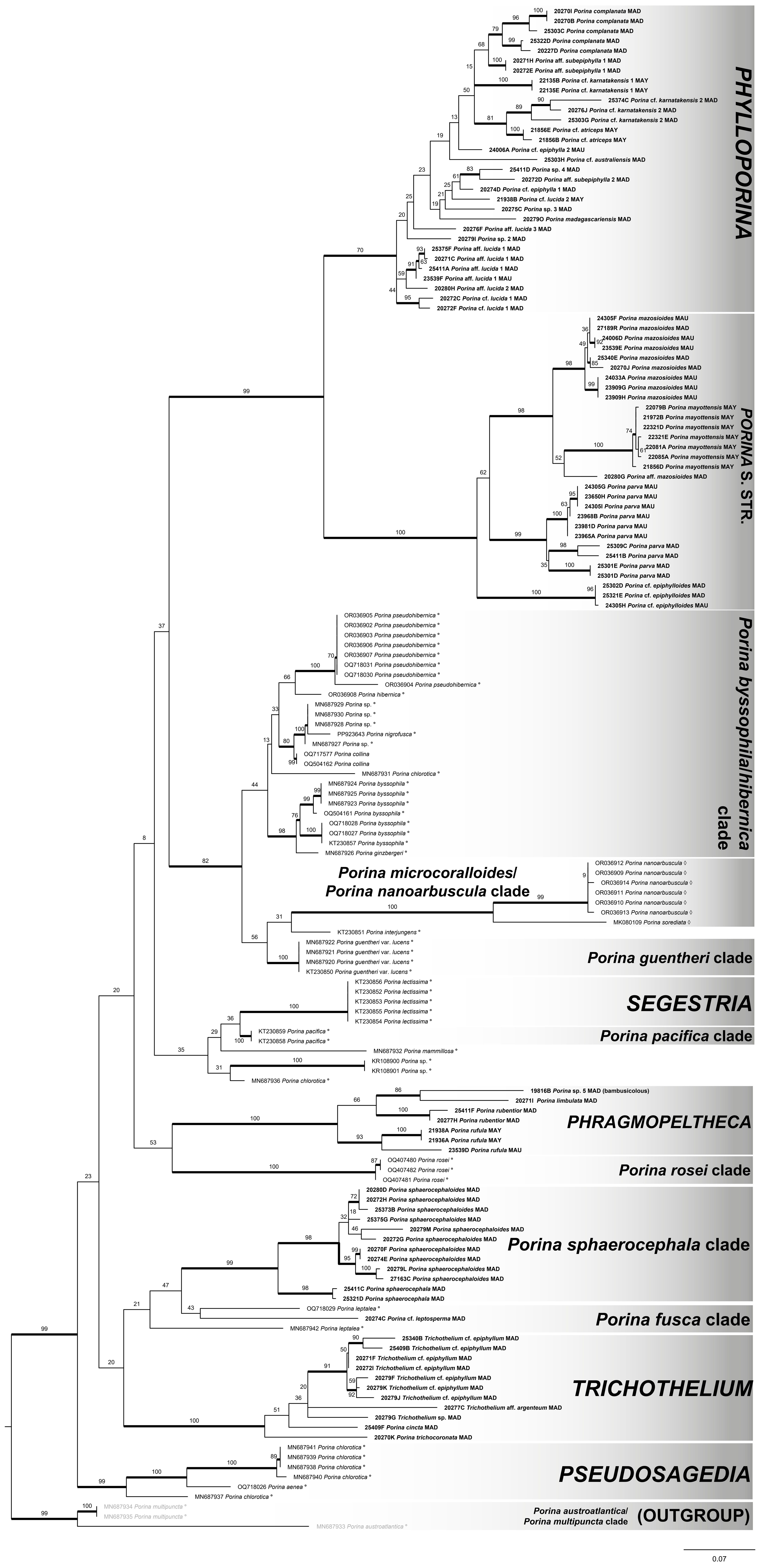
PHYLLOPORINA
20270I Porina complanata MAD
20270B Porina complanata MAD
25303C Porina complanata MAD
25322D Porina complanata MAD
20227D Porina complanata MAD
20271H Porina aff. subepiphylla 1 MAD
20272E Porina aff. subepiphylla 1 MAD
22135B Porina cf. karnatakensis 1 MAY
22135E Porina cf. karnatakensis 1 MAY
25374C Porina cf. karnatakensis 2 MAD
20276J Porina cf. karnatakensis 2 MAD
25303G Porina cf. karnatakensis 2 MAD
21856E Porina cf. atriceps MAY
21856B Porina cf. atriceps MAY
24006A Porina cf. epiphylla 2 MAU
25303H Porina cf. australiensis MAD
25411D Porina sp. 4 MAD
20272D Porina aff. subepiphylla 2 MAD
20274D Porina cf. epiphylla 1 MAD
21938B Porina cf. lucida 2 MAY
20275C Porina sp. 3 MAD
20279O Porina madagascariensis MAD
20276F Porina aff. lucida 3 MAD
20279I Porina sp. 2 MAD
25375F Porina aff. lucida 1 MAD
20271C Porina aff. lucida 1 MAD
25411A Porina aff. lucida 1 MAD
23539F Porina aff. lucida 1 MAU
20280H Porina aff. lucida 2 MAD
20272C Porina cf. lucida 1 MAD
20272F Porina cf. lucida 1 MAD
PORINA S. STR.
24305F Porina mazosioides MAU
27189R Porina mazosioides MAD
24006D Porina mazosioides MAU
23539E Porina mazosioides MAU
25340E Porina mazosioides MAD
20270J Porina mazosioides MAD
24033A Porina mazosioides MAU
23909G Porina mazosioides MAU
23909H Porina mazosioides MAU
22079B Porina mayottensis MAY
21972B Porina mayottensis MAY
22321D Porina mayottensis MAY
22321E Porina mayottensis MAY
22081A Porina mayottensis MAY
22085A Porina mayottensis MAY
21856D Porina mayottensis MAY
20280G Porina aff. mazosioides MAD
24305G Porina parva MAU
23650H Porina parva MAU
24305I Porina parva MAU
23968B Porina parva MAU
23981D Porina parva MAU
23965A Porina parva MAU
25309C Porina parva MAD
25411B Porina parva MAD
25301E Porina parva MAD
25301D Porina parva MAD
25302D Porina cf. epiphylloides MAD
25321E Porina cf. epiphylloides MAD
24305H Porina cf. epiphylloides MAU
Porina byssophila/hibernica clade
OR036905 Porina pseudohibernica °
OR036902 Porina pseudohibernica °
OR036903 Porina pseudohibernica °
OR036906 Porina pseudohibernica °
OR036907 Porina pseudohibernica °
OQ718031 Porina pseudohibernica °
OQ718030 Porina pseudohibernica °
OR036904 Porina pseudohibernica °
OR036908 Porina hibernica °
MN687929 Porina sp. °
MN687930 Porina sp. °
MN687928 Porina sp. °
PP923643 Porina nigrofusca °
MN687927 Porina sp. °
OQ717577 Porina collina
OQ504162 Porina collina
MN687931 Porina chlorotica °
MN687924 Porina byssophila °
MN687925 Porina byssophila °
MN687923 Porina byssophila °
OQ504161 Porina byssophila °
OQ718028 Porina byssophila °
OQ718027 Porina byssophila °
KT230857 Porina byssophila °
MN687926 Porina ginzbergeri °
Porina microcoralloides/
Porina nanoarbuscula clade
OR036912 Porina nanoarbuscula ◊
OR036909 Porina nanoarbuscula ◊
OR036914 Porina nanoarbuscula ◊
OR036911 Porina nanoarbuscula ◊
OR036910 Porina nanoarbuscula ◊
OR036913 Porina nanoarbuscula ◊
MK080109 Porina sorediata ◊
KT230851 Porina interjungens °
Porina guentheri clade
MN687922 Porina guentheri var. lucens °
MN687921 Porina guentheri var. lucens °
MN687920 Porina guentheri var. lucens °
KT230850 Porina guentheri var. lucens °
SEGESTRIA
KT230856 Porina lectissima °
KT230852 Porina lectissima °
KT230853 Porina lectissima °
KT230855 Porina lectissima °
KT230854 Porina lectissima °
Porina pacifica clade
KT230859 Porina pacifica °
KT230858 Porina pacifica °
MN687932 Porina mammillosa °
KR108900 Porina sp. °
KR108901 Porina sp. °
MN687936 Porina chlorotica °
PHRAGMOPELTHECA
19816B Porina sp. 5 MAD (bambusicolous)
20271I Porina limbulata MAD
25411F Porina rubentior MAD
20277H Porina rubentior MAD
21938A Porina rufula MAY
21936A Porina rufula MAY
23539D Porina rufula MAU
Porina rosei clade
OQ407480 Porina rosei °
OQ407482 Porina rosei °
OQ407481 Porina rosei °
Porina sphaerocephala clade
20280D Porina sphaerocephaloides MAD
20272H Porina sphaerocephaloides MAD
25373B Porina sphaerocephaloides MAD
25375G Porina sphaerocephaloides MAD
20279M Porina sphaerocephaloides MAD
20272G Porina sphaerocephaloides MAD
20270F Porina sphaerocephaloides MAD
20274E Porina sphaerocephaloides MAD
20279L Porina sphaerocephaloides MAD
27163C Porina sphaerocephaloides MAD
25411C Porina sphaerocephala MAD
25321D Porina sphaerocephala MAD
Porina fusca clade
OQ718029 Porina leptalea °
20274C Porina cf. leptosperma MAD
MN687942 Porina leptalea °
TRICHOTHELIUM
25340B Trichothelium cf. epiphyllum MAD
25409B Trichothelium cf. epiphyllum MAD
20271F Trichothelium cf. epiphyllum MAD
20272I Trichothelium cf. epiphyllum MAD
20279F Trichothelium cf. epiphyllum MAD
20279K Trichothelium cf. epiphyllum MAD
20279J Trichothelium cf. epiphyllum MAD
20277C Trichothelium aff. argenteum MAD
20279G Trichothelium sp. MAD
25409F Porina cincta MAD
20270K Porina trichocoronata MAD
PSEUDOSAGEDIA
MN687941 Porina chlorotica °
MN687939 Porina chlorotica °
MN687938 Porina chlorotica °
MN687940 Porina chlorotica °
OQ718026 Porina aenea °
MN687937 Porina chlorotica °
Porina austroatlantica/
Porina multipuncta clade
(OUTGROUP)
MN687934 Porina multipuncta °
MN687935 Porina multipuncta °
MN687933 Porina austroatlantica °
0.07
