# Supplementary material for: Integrative taxonomy reveals hidden fungal and algal diversity in foliicolous Porinaceae (Ascomycota, Ostropales) from Madagascar and the Indian Ocean Islands
Source: IMA Fungus. 2026 Nov 5;17:e207656. doi: 10.3897/imafungus.17.207656 (PMC13760347; doi:10.3897/imafungus.17.207656)
Supplement: Supplementary file 3 [file imafungus-17-e207656-s005.pdf]

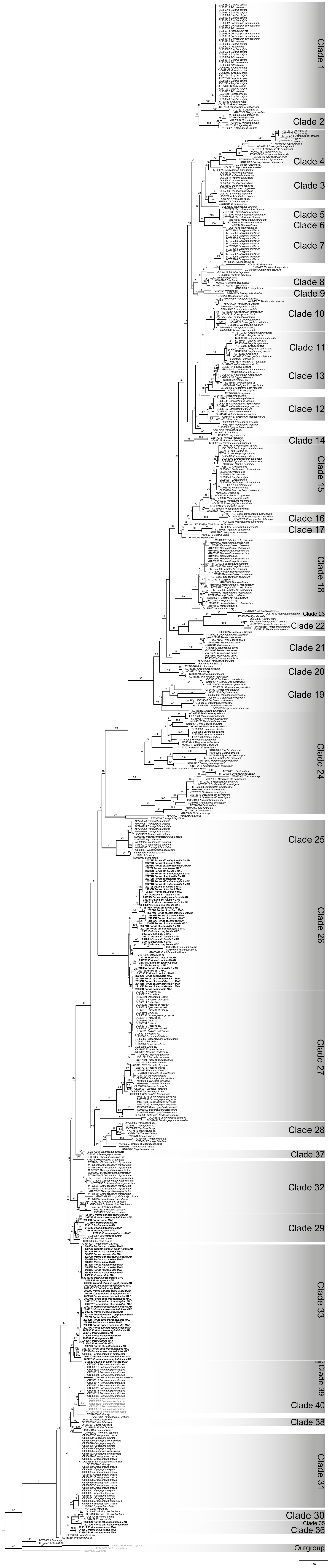

Clade 1
Clade 2
Clade 4
Clade 3
Clade 5
Clade 6
Clade 7
Clade 8
Clade 9
Clade 10
Clade 11
Clade 13
Clade 12
Clade 14
Clade 15
Clade 16
Clade 17
Clade 18
Clade 23
Clade 22
Clade 21
Clade 20
Clade 19
Clade 24
Clade 25
Clade 26
Clade 27
Clade 28
Clade 37
Clade 32
Clade 29
Clade 33
Clade 34
Clade 39
Clade 40
Clade 38
Clade 31
Clade 30
Clade 35
Clade 36
Outgroup
0.07
