# Supplementary material for: Integrative taxonomy reveals hidden fungal and algal diversity in foliicolous Porinaceae (Ascomycota, Ostropales) from Madagascar and the Indian Ocean Islands
Source: IMA Fungus. 2026 Nov 5;17:e207656. doi: 10.3897/imafungus.17.207656 (PMC13760347; doi:10.3897/imafungus.17.207656)
Supplement: Supplementary file 4 [file imafungus-17-e207656-s001.docx]

**Table 1 –** Species names, voucher specimens, GenBank Accession numbers of mtSSU and ITS sequences used in this study, and associated references. Newly generated sequences are in bold. is. = island.

| **Name** | **Voucher** | **mtSSU** | **ITS** | **Reference** |
| --- | --- | --- | --- | --- |
| *Coenogonium leprieurii* | AFTOL-ID 351 | AY584698 | / | (Lutzoni et al. 2004) |
| *Coenogonium luteum* | AFTOL-ID 352 | AY584699 | / | (Lutzoni et al. 2004) |
| *Coenogonium pineti* | 24 Jan. 02 *Palice* | AY300884 | / | (Lumbsch et al. 2003) |
| *Flabelloporina squamulifera* | Brazil, *Lücking & Caceres 39729* | MH457127 | / | (Sobreira et al. 2018) |
| *Gallaicolichen pacificus* | New Caledonia, *EL1811a* | PP080177 | / | (Lebreton et al. 2024) |
| *Gallaicolichen pacificus* | New Caledonia, *EL1812b* | PP080178 | / | (Lebreton et al. 2024) |
| *Myeloconis erumpens* | Thailand, *Lücking 24061* | KJ449322 | / | (Nelsen et al. 2014) |
| *Myeloconis fecunda* | Peru, *Nelsen s.n.* | KJ449319 | / | (Nelsen et al. 2014) |
| *Myeloconis guyanensis* | Puerto Rico, *Mercado-Diaz 3372 F53* | KJ449326 | / | (Nelsen et al. 2014) |
| *Porina aenea* | Austria, *Gruber s.n.-F1294* | DQ168411 | / | (Baloch and Grube 2006) |
| *Porina aenea* | Czech Republic, *PRA-Vondrak 24834* | OQ646397 | / | (Vondrák et al. 2023) |
| *Porina aenea* | Czech Republic, *PRA-Vondrak 25464* | OQ646398 | OQ718026 | (Vondrák et al. 2023) |
| *Porina aenea* | Czech Republic, *PRA-Palice 33213b* | OQ683135 | / | (Vondrák et al. 2023) |
| ***Porina* aff. *epiphylla*** | **Mayotte is., *Ertz 22131H*** | **QB133774** | **/** | Present study |
| ***Porina* aff. *lucida* 1** | **Mauritius is., *Ertz 23539H*** | **QB133761** | **/** | Present study |
| ***Porina* aff. *lucida* 1** | **Madagascar, *Ertz 20271C*** | **QB133762** | **QB133832** | Present study |
| ***Porina* aff. *lucida* 1** | **Madagascar, *Ertz 25375F*** | **QB133763** | **QB133833** | Present study |
| ***Porina* aff. *lucida* 1** | **Mauritius is., *Ertz 23539F*** | **QB133764** | **QB133831** | Present study |
| ***Porina* aff. *lucida* 1** | **Madagascar, *Ertz 25303F*** | **QB133765** | **/** | Present study |
| ***Porina* aff. *lucida* 1** | **Madagascar, *Ertz 25411A*** | **QB133766** | **QB133834** | Present study |
| ***Porina* aff. *lucida* 2** | **Madagascar, *Ertz 20280H*** | **QB133768** | **QB133835** | Present study |
| ***Porina* aff. *lucida* 3** | **Madagascar, *Ertz 20276F*** | **QB133773** | **QB133839** | Present study |
| ***Porina* aff*. mazosioides*** | **Madagascar, *Ertz 25321F*** | **QB133747** | **/** | Present study |
| ***Porina* aff*. mazosioides*** | **Madagascar, *Ertz 20280G*** | **QB133748** | **QB133874** | Present study |
| ***Porina* aff. s*ubepiphylla* 1** | **Madagascar, *Ertz 20272E*** | **QB133793** | **QB133851** | Present study |
| ***Porina* aff. s*ubepiphylla* 1** | **Madagascar, *Ertz 20271H*** | **QB133794** | **QB133852** | Present study |
| ***Porina* aff. s*ubepiphylla* 2** | **Madagascar, *Ertz 20272D*** | **QB133777** | **QB133844** | Present study |
| *Porina africana* | Mauritius is., *Ertz 23957* | OK058474 | / | (Ertz and Diederich 2022) |
| *Porina africana* | Mauritius is., *Ertz 24298* | OK058475 | / | (Ertz and Diederich 2022) |
| *Porina alba* | Costa Rica, *Baloch CR126* | AY648894 | / | (Grube et al. 2004) |
| *Porina alba* | Costa Rica, *Lücking 16001o* | DQ168372 | / | (Baloch and Grube 2006) |
| *Porina alba* | Costa Rica, *Baloch CR404-F1117* | FJ711086 | / | (Baloch and Grube 2009) |
| *Porina alba* | Costa Rica, *Baloch CR657-F984* | FJ711088 | / | (Baloch and Grube 2009) |
| *Porina alba* | Costa Rica, *Baloch CR610-F1255* | FJ711089 | / | (Baloch and Grube 2009) |
| *Porina alba* | Guadeloupe, *EL879a* | PP080173 | / | (Lebreton et al. 2024) |
| *Porina alba* | Guadeloupe, *EL880a1* | PP080174 | / | (Lebreton et al. 2024) |
| *Porina andreana* | China, *YN222512* (unpublished) | OQ550270 | / | Unpublished |
| *Porina atriceps* | China, *YN18305* (unpublished) | OM219174 | / | Unpublished |
| *Porina atrocoerulea* | Mexico, *Herrera-Campos S39P3-F1048* | DQ168389 | / | (Baloch and Grube 2006) |
| *Porina austroatlantica* | Falkland Islands, *Orange 22963* | / | MN687933 | (Orange et al. 2020) |
| *Porina austroatlantica* | Falkland Islands, *Orange 22606* | KR108903 | / | (Orange 2015) |
| *Porina bellendenica* | China, *YN18167* (unpublished) | MT526256 | / | Unpublished |
| *Porina bellendenica* | China, *YN18183* (unpublished) | MT526257 | / | Unpublished |
| *Porina borreri* | Belgium, *Ertz 12130* | OK058476 | / | (Ertz and Diederich 2022) |
| *Porina borreri* | Belgium, *Ertz 25837* | OK058477 | / | (Ertz and Diederich 2022) |
| *Porina byssophila* | United Kingdom, *Orange 16444* | / | MN687923 | (Orange et al. 2020) |
| *Porina byssophila* | United Kingdom, *Orange 17561* | / | MN687924 | (Orange et al. 2020) |
| *Porina byssophila* | United Kingdom, *Orange 23938* | / | MN687925 | (Orange et al. 2020) |
| *Porina byssophila* | Belgium, *Ertz 25750* | / | OQ504161 | (Ertz 2023) |
| *Porina byssophila* | Czech Republic, *PRA-Vondrak 24308* | / | OQ718027 | (Vondrák et al. 2023) |
| *Porina byssophila* | United Kingdom, *Orange 21981* | KR108915 | KT230857 | (Orange 2015) |
| *Porina byssophila* | Belgium, *Ertz 26030* | OQ468268 | / | (Ertz 2023) |
| *Porina byssophila* | Czech Republic, *PRA-Vondrak 24786* | OQ646399 | OQ718028 | (Vondrák et al. 2023) |
| ***Porina* cf. *atriceps*** | **Mayotte is., *Ertz 21856B*** | **QB133784** | **QB133853** | Present study |
| ***Porina* cf. *atriceps*** | **Mayotte is., *Ertz 21856C*** | **QB133785** | **/** | Present study |
| ***Porina* cf. *atriceps*** | **Mayotte is., *Ertz 21856E*** | **QB133786** | **QB133854** | Present study |
| ***Porina* cf. *australiensis*** | **Madagascar, *Ertz 25303H*** | **QB133799** | **QB133861** | Present study |
| *Porina* cf. *eminentior* | Mauritius is., *Ertz 23493* | OK058481 | / | (Ertz and Diederich 2022) |
| *Porina* cf. *epiphylla* | Mauritius is., *Ertz 23526* | OK058482 | / | (Ertz and Diederich 2022) |
| *Porina* cf. *epiphylla* | Mauritius is., *Ertz 24345* | OK058483 | / | (Ertz and Diederich 2022) |
| ***Porina* cf. *epiphylla* 1** | **Madagascar, *Ertz 20274D*** | **QB133778** | **QB133841** | Present study |
| ***Porina* cf. *epiphylla* 2** | **Mauritius is., *Ertz 24006A.1*** | **QB133797** | **/** | Present study |
| ***Porina* cf. *epiphylla* 2** | **Mauritius is., *Ertz 24006A.2*** | **QB133798** | **QB133842** | Present study |
| ***Porina* cf*. epiphylloides*** | **Madagascar, *Ertz 2*5321E** | **/** | **QB133863** | Present study |
| ***Porina* cf*. epiphylloides*** | **Mauritius is., *Ertz 24305H*** | **QB133759** | **QB133862** | Present study |
| ***Porina* cf*. epiphylloides*** | **Madagascar, *Ertz 25302D*** | **QB133760** | **QB133864** | Present study |
| *Porina* cf. *internigrans* | Mauritius is., *Ertz 23519* | OK058492 | / | (Ertz and Diederich 2022) |
| *Porina* cf. *internigrans* | Mauritius is., *Ertz 23956* | OK058493 | / | (Ertz and Diederich 2022) |
| ***Porina* cf. *karnatakensis* 1** | **Mayotte is., *Ertz 22135E*** | **QB133795** | **QB133859** | Present study |
| ***Porina* cf. *karnatakensis* 1** | **Mayotte is., *Ertz 22135B*** | **QB133796** | **QB133860** | Present study |
| ***Porina* cf. *karnatakensis* 2** | **Madagascar, *Ertz 20276J*** | **QB133780** | **QB133858** | Present study |
| ***Porina* cf. *karnatakensis* 2** | **Madagascar, *Ertz 25374C*** | **QB133781** | **QB133857** | Present study |
| ***Porina* cf. *karnatakensis* 2** | **Madagascar, *Ertz 25303G*** | **QB133782** | **QB133856** | Present study |
| ***Porina* cf. *leptosperma*** | **Madagascar, *Ertz 20274C*** | **QB133717** | **QB133800** | Present study |
| ***Porina* cf. *lucida* 1** | **Madagascar, *Ertz 20272C*** | **QB133769** | **QB133837** | Present study |
| ***Porina* cf. *lucida* 1** | **Madagascar, *Ertz 20270H*** | **QB133770** | **/** | Present study |
| ***Porina* cf. *lucida* 1** | **Madagascar, *Ertz 20272F*** | **QB133771** | **QB133838** | Present study |
| ***Porina* cf. *lucida* 2** | **Mayotte is., *Ertz 21938B*** | **QB133779** | **QB133840** | Present study |
| *Porina* cf. *malmei* | Madagascar, *Ertz 25249-hymenium* | OK058496 | / | (Ertz and Diederich 2022) |
| *Porina* cf. *malmei* | Madagascar, *Ertz 25249-thallus* | OK058497 | / | (Ertz and Diederich 2022) |
| *Porina* cf. *scabrida* | Florida, *WBS 21212.7* | OR036942 | / | (Sanders et al. 2023) |
| *Porina* cf. *scabrida* | Florida, *WBS 21320.3* | OR036943 | / | (Sanders et al. 2023) |
| *Porina chlorotica* | Ireland, *Orange 18225* | / | MN687931 | (Orange et al. 2020) |
| *Porina chlorotica* | United Kingdom, *Orange 17299* | / | MN687936 | (Orange et al. 2020) |
| *Porina chlorotica* | United Kingdom, *Orange 24146* | / | MN687937 | (Orange et al. 2020) |
| *Porina chlorotica* | Ireland, *Orange 18154* | / | MN687938 | (Orange et al. 2020) |
| *Porina chlorotica* | United Kingdom, *Orange 22748* | / | MN687940 | (Orange et al. 2020) |
| *Porina chlorotica* | United Kingdom, *Orange 23600* | / | MN687941 | (Orange et al. 2020) |
| *Porina chlorotica* | United Kingdom, *Orange 17307* | KR108907 | MN687939 | (Orange 2015) |
| ***Porina cincta*** | **Madagascar, *Ertz 25409F*** | **QB133723** | **QB133821** | Present study |
| *Porina collina* | Belgium, *Ertz 26432* | / | OQ504162 | (Ertz 2023) |
| *Porina collina* | Czech Republic, *PRA-Palice 29243* | / | OQ717577 | (Vondrák et al. 2023) |
| *Porina collina* | United Kingdom, *Orange 24073* | MN687916 | / | (Orange et al. 2020) |
| ***Porina complanata*** | **Madagascar, *Ertz 20227D*** | **QB133787** | **QB133846** | Present study |
| ***Porina complanata*** | **Madagascar, *Ertz 25322D*** | **QB133788** | **QB133847** | Present study |
| ***Porina complanata*** | **Madagascar, *Ertz 25321B*** | **QB133789** | **/** | Present study |
| ***Porina complanata*** | **Madagascar, *Ertz 20270I*** | **QB133790** | **QB133850** | Present study |
| ***Porina complanata*** | **Madagascar, *Ertz 20270B*** | **QB133791** | **QB133849** | Present study |
| ***Porina complanata*** | **Madagascar, *Ertz 25303C*** | **QB133792** | **QB133848** | Present study |
| *Porina conica* | China, *YN18286-4* (unpublished) | MT526252 | / | Unpublished |
| *Porina conica* | China, *YN210923-1* (unpublished) | OM219169 | / | Unpublished |
| *Porina conica* | China, *YN210915-1* (unpublished) | OR164498 | / | Unpublished |
| *Porina covidii* | Mauritius is., *Ertz 23311*-hymenium | OK058478 | / | (Ertz and Diederich 2022) |
| *Porina covidii* | Mauritius is., *Ertz 23311*-thallus | OK058479 | / | (Ertz and Diederich 2022) |
| *Porina covidii* | Rodrigues is., *Ertz 23780* | OK058480 | / | (Ertz and Diederich 2022) |
| *Porina cryptostoma* | Costa Rica, *Lücking 16117a* | KJ449308 | / | (Nelsen et al. 2014) |
| *Porina cupreola* | China, *YN222406-3* (unpublished) | OR164484 | / | Unpublished |
| *Porina dolichophora* | Costa Rica, *Lücking AS HT1* | KJ449306 | / | (Nelsen et al. 2014) |
| *Porina epiphylla* | Costa Rica, *Baloch CR103* | AY648892 | / | (Grube et al. 2004) |
| *Porina epiphylla* | Costa Rica, *Baloch CR635-F928* | DQ168373 | / | (Baloch and Grube 2006) |
| *Porina epiphylla* | Costa Rica, *Grube CR901-F906* | DQ168374 | / | (Baloch and Grube 2006) |
| *Porina epiphylla* | Costa Rica, *Baloch CR265/3-F535* | FJ711096 | / | (Baloch and Grube 2009) |
| *Porina epiphylla* | Costa Rica, *Baloch CR1120-F1506* | FJ711098 | / | (Baloch and Grube 2009) |
| *Porina epiphylla* | Costa Rica, *Baloch CR432-F762* | FJ711103 | / | (Baloch and Grube 2009) |
| *Porina epiphylla* | Costa Rica, *Lücking RL16024_F346* | FJ711106 | / | (Baloch and Grube 2009) |
| *Porina epiphylla* | Mexico, *Herrera-Campos SP-F1058* | FJ711107 | / | (Baloch and Grube 2009) |
| *Porina epiphylla* | *Lutzoni et al. 03.24.03-10* | KJ766464 | / | (Miadlikowska et al. 2014) |
| *Porina epiphylloides* | Mauritius is., *Ertz 24343* | OK058484 | **/** | (Ertz and Diederich 2022) |
| *Porina epiphylloides* | Mauritius is., *Ertz 24343* | OK058484 | / | (Ertz and Diederich 2022) |
| *Porina exasperatula* | Panama, *Lücking Pan-Comp. PAN-18* | KJ449316 | / | (Nelsen et al. 2014) |
| *Porina exocha* | *Lumbsch 19108g* | KF833333 | / | Unpublished |
| *Porina farinosa* | Panama, *Lücking Panama-Crane, Pan-03* | KJ449312 | / | (Nelsen et al. 2014) |
| *Porina florensii* | Mauritius is., *Diederich 18348* | MN989205 | / | (Diederich and Ertz 2020) |
| *Porina florensii* | Mauritius is., *Diederich 18453* | MN989206 | / | (Diederich and Ertz 2020) |
| *Porina florensii* | Mauritius is., *Ertz 23489* | OK058485 | / | (Ertz and Diederich 2022) |
| *Porina florensii* | Mauritius is., *Ertz 24283* | OK058486 | / | (Ertz and Diederich 2022) |
| *Porina florensii* | China, *FJ220176* (unpublished) | OR164486 | / | Unpublished |
| *Porina florensii* | China, *HN19538* (unpublished) | OR164500 | / | Unpublished |
| *Porina fusca* | Costa Rica, *Baloch CR757-F1673* | DQ168398 | / | (Baloch and Grube 2006) |
| *Porina ginzbergeri* | United Kingdom, *Orange 21712* | / | MN687926 | (Orange et al. 2020) |
| *Porina griffithsii* | Mauritius is., *Ertz 23581* | OK058487 | / | (Ertz and Diederich 2022) |
| *Porina griffithsii* | Mauritius is., *Ertz 23610* | OK058488 | / | (Ertz and Diederich 2022) |
| *Porina griffithsii* | Mauritius is., *Ertz 23632* | OK058489 | / | (Ertz and Diederich 2022) |
| *Porina griffithsii* | Rodrigues is., *Ertz 23703pp* | OK058490 | / | (Ertz and Diederich 2022) |
| *Porina griffithsii* | Rodrigues is., *Ertz 23887* | OK058491 | / | (Ertz and Diederich 2022) |
| *Porina guentheri* var. *lucens* | United Kingdom, *Orange 16677* | / | KT230850 | (Orange 2015) |
| *Porina guentheri* var. *lucens* | United Kingdom, *Orange 20859* | / | MN687920 | (Orange et al. 2020) |
| *Porina guentheri* var. *lucens* | United Kingdom, *Orange 24224* | / | MN687921 | (Orange et al. 2020) |
| *Porina guentheri* var. *lucens* | Ireland, *Orange 18248* | / | MN687922 | (Orange et al. 2020) |
| *Porina guianensis* | Costa Rica, *Baloch CR1021-F1597* | DQ168384 | / | (Baloch and Grube 2006) |
| *Porina heterospora* | Brazil, *Nelsen s. n.* | KJ449318 | / | (Nelsen et al. 2014) |
| *Porina hibernica* | United Kingdom, *N.A.S 2894* | OR036953 | / | (Sanders et al. 2023) |
| *Porina hibernica* | United Kingdom, *N.A.S 2896* | OR036954 | OR036908 | (Sanders et al. 2023) |
| *Porina imitatrix* | Panama, *Lücking Pan26* | KJ449313 | / | (Nelsen et al. 2014) |
| *Porina imitatrix* | Panama, *Lücking Pan-Comp. PAN-17* | KJ449315 | / | (Nelsen et al. 2014) |
| *Porina interjungens* | United Kingdom, *Orange 16686* | / | KT230851 | (Orange 2015) |
| *Porina internigrans* | Costa Rica, *Baloch CR886-F1338* | DQ168413 | / | (Baloch and Grube 2006) |
| *Porina internigrans* | China, *GZ18144* (unpublished) | MT526253 | / | Unpublished |
| *Porina internigrans* | China, *FJ220226-1* (unpublished) | OR164494 | / | Unpublished |
| *Porina internigrans* | China, *20200141* (unpublished) | OR164495 | / | Unpublished |
| *Porina internigrans* | China, *FJ211233* (unpublished) | OR164501 | / | Unpublished |
| *Porina internigrans* | China, *FJ220173* (unpublished) | OR164503 | / | Unpublished |
| *Porina internigrans* | China, *GX201083* (unpublished) | OR164504 | / | Unpublished |
| *Porina kamerunensis* | Japan, *K. Miyazawa & Y. Ohmura KeM504* | LC648416 | / | (Miyazawa et al. 2022) |
| *Porina kamerunensis* | Japan, *TNS:K. Miyazawa 666* | LC913203 | / | (Miyazawa and Ohmura 2026) |
| *Porina kamerunensis* | Japan, *TNS:K. Miyazawa 762* | LC913204 | / | (Miyazawa and Ohmura 2026) |
| *Porina kamerunensis* | Japan, *TNS:K. Miyazawa 1108* | LC913205 | / | (Miyazawa and Ohmura 2026) |
| *Porina kamerunensis* | Japan, *TNS:K. Miyazawa 1113* | LC913206 | / | (Miyazawa and Ohmura 2026) |
| *Porina kamerunensis* | Japan, *TNS:K. Miyazawa 1300* | LC913207 | / | (Miyazawa and Ohmura 2026) |
| *Porina kamerunensis* | China, *FJ220190-2* (unpublished) | OR164483 | / | Unpublished |
| *Porina karnatakensis* | Costa Rica, *Baloch CR729_F1419* | DQ168375 | / | (Baloch and Grube 2006) |
| *Porina karnatakensis* | Costa Rica, *Baloch CR567/1_F1163* | FJ711112 | / | (Baloch and Grube 2009) |
| *Porina karnatakensis* | Costa Rica, *Baloch CR732/1_F1401* | FJ711115 | / | (Baloch and Grube 2009) |
| *Porina karnatakensis* | Costa Rica, *Baloch CR679_F855* | FJ711119 | / | (Baloch and Grube 2009) |
| *Porina karnatakensis* | China, *YN210801-1* (unpublished) | OM219172 | / | Unpublished |
| *Porina karnatakensis* | China, *YN211114* (unpublished) | OM219173 | / | Unpublished |
| *Porina karnatakensis* | China, *YN210915-3* (unpublished) | OM219175 | / | Unpublished |
| *Porina karnatakensis* | China, *YN222411* (unpublished) | OR164505 | / | Unpublished |
| *Porina karnatakensis* | China, FJ*220190* (unpublished) | OR164506 | / | Unpublished |
| *Porina lectissima* | United Kingdom, *Orange 20441* | / | KT230852 | (Orange 2015) |
| *Porina lectissima* | United Kingdom, *Orange 20462* | / | KT230853 | (Orange 2015) |
| *Porina lectissima* | United Kingdom, *Orange 20855* | / | KT230854 | (Orange 2015) |
| *Porina lectissima* | Ireland, *Orange 21436* | / | KT230855 | (Orange 2015) |
| *Porina lectissima* | United Kingdom, *Orange 21663* | / | KT230856 | (Orange 2015) |
| *Porina lectissima* | Austria, *Grube s.n.-F1464* | DQ168414 | / | (Baloch and Grube 2006) |
| *Porina leptalea* | Falkland Islands, *Orange 22282* | / | MN687942 | (Orange et al. 2020) |
| *Porina leptalea* | Belgium, *Ertz 23175* | MT831494 | / | (Ertz et al. 2021) |
| *Porina leptalea* | Mauritius is., *Ertz 21473* | OK058494 | / | (Ertz and Diederich 2022) |
| *Porina leptalea* | Czech Republic, *PRA-Vondrak 24457* | OQ646400 | OQ718029 | (Vondrák et al. 2023) |
| *Porina leptalea* | Czech Republic, *PRA-Vondrak 24458* | OQ646401 | / | (Vondrák et al. 2023) |
| *Porina limbulata* | Costa Rica, *Baloch CR026/1* | AY648893 | / | (Grube et al. 2004) |
| *Porina limbulata* | Costa Rica, *Baloch CR330-F755* | DQ168402 | / | (Baloch and Grube 2006) |
| *Porina limbulata* | Costa Rica, *Baloch CR535-F1111* | DQ168403 | / | (Baloch and Grube 2006) |
| *Porina limbulata* | Costa Rica, *Baloch CR458-F1225* | DQ168404 | / | (Baloch and Grube 2006) |
| ***Porina limbulata*** | **Madagascar, *Ertz 20271G*** | **QB133696** | **/** | Present study |
| ***Porina limbulata*** | **Madagascar, *Ertz 20271I*** | **QB133697** | **QB133814** | Present study |
| *Porina limitata* | China, *FJ220212* (unpublished) | OR164496 | / | Unpublished |
| *Porina limitata* | China, *FJ220213* (unpublished) | OR164497 | / | Unpublished |
| *Porina longispora* | Mauritius is., *Ertz 23524* | OK058495 | / | (Ertz and Diederich 2022) |
| *Porina lucida* | Costa Rica, *Baloch CR750_F1440* | DQ168378 | / | (Baloch and Grube 2006) |
| *Porina lucida* | Costa Rica, *Baloch CR79_F90* | FJ711128 | / | (Baloch and Grube 2009) |
| *Porina lucida* | Costa Rica, *Lücking RL_F343* | FJ711130 | / | (Baloch and Grube 2009) |
| *Porina lucida* | Costa Rica, *Baloch CR664_F1196* | FJ711134 | / | (Baloch and Grube 2009) |
| *Porina lucida* | Costa Rica, *Baloch CR372_F955* | FJ711137 | / | (Baloch and Grube 2009) |
| ***Porina madagacariensis*** | **Madagascar, *Ertz 20279O*** | **QB133783** | **QB133855** | Present study |
| *Porina mammillosa* | United Kingdom, *Orange 24142* | MN687918 | MN687932 | (Orange et al. 2020) |
| *Porina mastoidea* | Costa Rica, *Baloch CR861-F1333* | DQ168382 | / | (Baloch and Grube 2006) |
| *Porina mastoidea* | Costa Rica, *Baloch CR869-F1377* | DQ168383 | / | (Baloch and Grube 2006) |
| *Porina mastoidella* | China, *YN18332* (unpublished) | MT526259 | / | Unpublished |
| *Porina mauritiana* | Mauritius is., *Ertz 23433* | OK058498 | / | (Ertz and Diederich 2022) |
| ***Porina mayottensis*** | **Mayotte is., *Ertz 22085A*** | **QB133739** | **QB133876** | Present study |
| ***Porina mayottensis*** | **Mayotte is., *Ertz 22321E*** | **QB133740** | **QB133877** | Present study |
| ***Porina mayottensis*** | **Mayotte is., *Ertz 22321D*** | **QB133741** | **QB133879** | Present study |
| ***Porina mayottensis*** | **Mayotte is., *Ertz 22081A*** | **QB133742** | **QB133880** | Present study |
| ***Porina mayottensis*** | **Mayotte is., *Ertz 21856D*** | **QB133743** | **QB133875** | Present study |
| ***Porina mayottensis*** | **Mayotte is., *Ertz 22079B*** | **QB133744** | **QB133881** | Present study |
| ***Porina mayottensis*** | **Mayotte is., *Ertz 21972B*** | **QB133745** | **QB133878** | Present study |
| ***Porina mayottensis*** | **Mayotte is., *Ertz 22069A*** | **QB133746** | **/** | Present study |
| *Porina mazosioides* | Mauritius is., *Ertz 23459* | OK058499 | / | (Ertz and Diederich 2022) |
| *Porina mazosioides* | Mauritius is., *Ertz 23525* | OK058500 | / | (Ertz and Diederich 2022) |
| ***Porina mazosioides*** | **Mauritius is., *Ertz 24006D*** | **QB133724** | **QB133866** | Present study |
| ***Porina mazosioides*** | **Madagascar, *Ertz 25329D*** | **QB133725** | **/** | Present study |
| ***Porina mazosioides*** | **Mauritius is., *Ertz 23909F*** | **QB133726** | **/** | Present study |
| ***Porina mazosioides*** | **Mauritius is., *Ertz 24305E*** | **QB133727** | **/** | Present study |
| ***Porina mazosioides*** | **Mauritius is., *Ertz 23539E*** | **QB133728** | **QB133865** | Present study |
| ***Porina mazosioides*** | **Madagascar, *Ertz 25340E*** | **QB133729** | **QB133870** | Present study |
| ***Porina mazosioides*** | **Madagascar, *Ertz 27195P*** | **QB133730** | **/** | Present study |
| ***Porina mazosioides*** | **Mauritius is., *Ertz 23981A*** | **QB133731** | **/** | Present study |
| ***Porina mazosioides*** | **Mauritius is., *Ertz 23459A*** | **QB133732** | **/** | Present study |
| ***Porina mazosioides*** | **Madagascar, *Ertz 20270J*** | **QB133733** | **QB133871** | Present study |
| ***Porina mazosioides*** | **Mauritius is., *Ertz 24305F*** | **QB133734** | **QB133872** | Present study |
| ***Porina mazosioides*** | **Mauritius is., *Ertz 23909G*** | **QB133735** | **QB133867** | Present study |
| ***Porina mazosioides*** | **Mauritius is., *Ertz 23909H*** | **QB133736** | **QB133869** | Present study |
| ***Porina mazosioides*** | **Mauritius is., *Ertz 24033A*** | **QB133737** | **QB133868** | Present study |
| ***Porina mazosioides*** | **Madagascar, *Ertz 27189R*** | **QB133738** | **QB133873** | Present study |
| *Porina microcoralloides* | Florida, *WBS 20423.11b* | OR036926 | / | (Sanders et al. 2023) |
| *Porina microcoralloides* | Florida, *WBS 20424.5* | OR036927 | / | (Sanders et al. 2023) |
| *Porina microcoralloides* | Florida, *WBS 20511.2* | OR036928 | / | (Sanders et al. 2023) |
| *Porina microcoralloides* | Florida, *WBS 20423.9* | OR036929 | / | (Sanders et al. 2023) |
| *Porina microcoralloides* | Florida, *WBS 20423.6* | OR036930 | / | (Sanders et al. 2023) |
| *Porina microcoralloides* | Florida, *WBS 20510.3* | OR036933 | / | (Sanders et al. 2023) |
| *Porina microcoralloides* | Florida, *WBS 20425.1* | OR036935 | / | (Sanders et al. 2023) |
| *Porina microcoralloides* | Florida, *WBS 20506.3* | OR036937 | / | (Sanders et al. 2023) |
| *Porina microcoralloides* | Florida, *WBS 21312.16* | OR036944 | / | (Sanders et al. 2023) |
| *Porina microcoralloides* | Florida, *WBS 21312.17* | OR036945 | / | (Sanders et al. 2023) |
| *Porina microcoralloides* | Florida, *WBS 21320.5* | OR036946 | / | (Sanders et al. 2023) |
| *Porina microcoralloides* | Florida, *WBS 21502.2* | OR036947 | / | (Sanders et al. 2023) |
| *Porina microcoralloides* | Florida, *WBS 21410.1* | OR036948 | / | (Sanders et al. 2023) |
| *Porina microcoralloides* | Florida, *WBS 21425.6* | OR036949 | / | (Sanders et al. 2023) |
| *Porina mirabilis* | *Baloch CR126* | AY648894 | / | (Grube et al. 2004) |
| *Porina multipuncta* | United Kingdom, *Orange 24329* | / | MN687934 | (Orange et al. 2020) |
| *Porina multipuncta* | United Kingdom, *Orange 23941* | / | MN687935 | (Orange et al. 2020) |
| *Porina multipuncta* | United Kingdom, *Coppins s.n.* | MK990615 | / | (Ertz et al. 2019) |
| *Porina nanoarbuscula* | Florida*, WBS 21320.1* | / | OR036914 | (Sanders et al. 2023) |
| *Porina nanoarbuscula* | Florida, *WBS 20511.3* | OR036931 | / | (Sanders et al. 2023) |
| *Porina nanoarbuscula* | Florida, *WBS 20423.1* | OR036932 | OR036909 | (Sanders et al. 2023) |
| *Porina nanoarbuscula* | Florida, *WBS 20424.4* | OR036934 | / | (Sanders et al. 2023) |
| *Porina nanoarbuscula* | Florida, *WBS 21403.5* | OR036938 | OR036913 | (Sanders et al. 2023) |
| *Porina nanoarbuscula* | Florida, *WBS 21421.5* | OR036939 | OR036912 | (Sanders et al. 2023) |
| *Porina nanoarbuscula* | Florida*, WBS 21421.8* | OR036940 | OR036911 | (Sanders et al. 2023) |
| *Porina nanoarbuscula* | Florida, *WBS 21502.2* | OR036941 | OR036910 | (Sanders et al. 2023) |
| *Porina nigrofusca* | India, *Shubham Pradhan s.n.* | / | PP923643 | Unpublished |
| *Porina nitidula* | Costa Rica, *Baloch CR455-F1216* | DQ168391 | / | (Baloch and Grube 2006) |
| *Porina nitidula* | Costa Rica, *Baloch CR1078-F1681* | DQ168392 | / | (Baloch and Grube 2006) |
| *Porina nitidula* | Costa Rica, *Lücking s.n.* | KF833334 | / | Unpublished |
| *Porina nitidula* | Japan, *TNS:K. Miyazawa 662* | LC913208 | / | (Miyazawa and Ohmura 2026) |
| *Porina nitidula* | Japan, *TNS:K. Miyazawa 673C* | LC913209 | / | (Miyazawa and Ohmura 2026) |
| *Porina nitidula* | Japan, *TNS:K. Miyazawa 790C* | LC913210 | / | (Miyazawa and Ohmura 2026) |
| *Porina nucula* | Costa Rica, *Lücking 17099* | DQ168387 | / | (Baloch and Grube 2006) |
| *Porina nucula* | Costa Rica, *Lücking 17007c* | KJ449310 | / | (Nelsen et al. 2014) |
| *Porina nucula* | Mauritius is., *Ertz 23762* | OK058501 | / | (Ertz and Diederich 2022) |
| *Porina nuculoides* | Mauritius is., *Ertz 23617* | OK058502 | / | (Ertz and Diederich 2022) |
| *Porina pacifica* | Canada*, Orange 22762* | / | KT230858 | (Orange 2015) |
| *Porina pacifica* | Canada*, Orange 22770* | KT254300 | KT230859 | (Orange 2015) |
| *Porina papillifera* | Costa Rica, *Baloch CR1095-F1688* | DQ168396 | / | (Baloch and Grube 2006) |
| ***Porina parva*** | **Madagascar, *Ertz 25309C*** | **QB133749** | **QB133882** | Present study |
| ***Porina parva*** | **Madagascar, *Ertz 25411B*** | **QB133750** | **QB133883** | Present study |
| ***Porina parva*** | **Madagascar, *Ertz 25301D*** | **QB133751** | **QB133884** | Present study |
| ***Porina parva*** | **Madagascar, *Ertz 25301E*** | **QB133752** | **QB133885** | Present study |
| ***Porina parva*** | **Mauritius is., *Ertz 23968B*** | **QB133753** | **QB133886** | Present study |
| ***Porina parva*** | **Mauritius is., *Ertz 24305G*** | **QB133754** | **QB133890** | Present study |
| ***Porina parva*** | **Mauritius is., *Ertz 24305I*** | **QB133755** | **QB133888** | Present study |
| ***Porina parva*** | **Mauritius is., *Ertz 23965A*** | **QB133756** | **QB133887** | Present study |
| ***Porina parva*** | **Mauritius is., *Ertz 23981D*** | **QB133757** | **QB133891** | Present study |
| ***Porina parva*** | **Mauritius is., *Ertz 23650H*** | **QB133758** | **QB133889** | Present study |
| *Porina perminuta* | Japan, *TNS:K. Miyazawa 1237* | LC913211 | / | (Miyazawa and Ohmura 2026) |
| *Porina perminuta* | Japan, *TNS:Y. Tochihara 1* | LC913212 | / | (Miyazawa and Ohmura 2026) |
| *Porina perminuta* | Mauritius is., *Diederich 18732* | OK058503 | / | (Ertz and Diederich 2022) |
| *Porina pilifera* | China, *YN210907-3* (unpublished) | OM219176 | / | Unpublished |
| *Porina pilifera* | China, *YN210920-2* (unpublished) | OM219177 | / | Unpublished |
| *Porina pilifera* | China, *FJ222520* (unpublished) | OR164499 | / | Unpublished |
| *Porina praestantior* | Mauritius is., *Ertz 23520* | OK058504 | / | (Ertz and Diederich 2022) |
| *Porina pseudohibernica* | Italy, *Muggia L4363* | / | OR036903 | (Sanders et al. 2023) |
| *Porina pseudohibernica* | Italy, *Muggia L4364* | / | OR036905 | (Sanders et al. 2023) |
| *Porina pseudohibernica* | Italy, *Muggia L4362* | / | OR036907 | (Sanders et al. 2023) |
| *Porina pseudohibernica* | Czech Republic, *PRA-Vondrak 25768* | OQ646402 | OQ718030 | (Vondrák et al. 2023) |
| *Porina pseudohibernica* | Czech Republic, *PRA-Vondrak 25769* | OQ646403 | OQ718031 | (Vondrák et al. 2023) |
| *Porina pseudohibernica* | Italy, *Muggia L4360* | OR036950 | OR036902 | (Sanders et al. 2023) |
| *Porina pseudohibernica* | Italy, *Muggia L4361* | OR036951 | OR036906 | (Sanders et al. 2023) |
| *Porina pseudohibernica* | Italy, *Muggia L4365* | OR036952 | OR036904 | (Sanders et al. 2023) |
| *Porina radiata* | Costa Rica, *Baloch CR106/1* | AY648895 | / | (Grube et al. 2004) |
| *Porina radiata* | Costa Rica, *Baloch CR1071-F1538* | DQ168386 | / | (Baloch and Grube 2006) |
| *Porina rosei* | Russia, *PRA-Vondrak 22938* | / | OQ407481 | (Malíček et al. 2023) |
| *Porina rosei* | Russia, *PRA-Vondrak 15450* | OQ407483 | OQ407480 | (Malíček et al. 2023) |
| *Porina rosei* | Russia, *PRA-Vondrak 22903* | OQ407484 | OQ407482 | (Malíček et al. 2023) |
| *Porina rosei* | Czech Republic, *J. Malicek 12126* | OQ407485 | / | (Malíček et al. 2023) |
| *Porina rosei* | Russia, *PRA-Vondrak 22318* | OQ683136 | / | (Vondrák et al. 2023) |
| *Porina rosei* | Belgium, *Ertz 27623* | PQ206317 | / | (Ertz 2024) |
| *Porina rubentior* | Costa Rica, *Baloch CR460-F1231* | DQ168405 | / | (Baloch and Grube 2006) |
| *Porina rubentior* | Costa Rica, *Baloch CR671A-F1212* | DQ168406 | / | (Baloch and Grube 2006) |
| ***Porina rubentior*** | **Madagascar, *Ertz 25411F*** | **QB133693** | **QB133815** | Present study |
| ***Porina rubentior*** | **Madagascar, *Ertz 20277H*** | **QB133694** | **QB133816** | Present study |
| ***Porina rubentior*** | **Madagascar, *Ertz 25411G*** | **QB133695** | **/** | Present study |
| *Porina rubescens* | Costa Rica, *Baloch CR1086-F1553* | DQ168397 | / | (Baloch and Grube 2006) |
| *Porina rufula* | Costa Rica, *Baloch CR496-F1087* | DQ168407 | / | (Baloch and Grube 2006) |
| *Porina rufula* | Costa Rica, *Baloch CR180-F641* | DQ168408 | / | (Baloch and Grube 2006) |
| *Porina rufula* | Japan, *TNS:K. Miyazawa 757C* | LC913213 | / | (Miyazawa and Ohmura 2026) |
| *Porina rufula* | Japan, *TNS:K. Miyazawa 769A* | LC913214 | / | (Miyazawa and Ohmura 2026) |
| *Porina rufula* | Japan, *TNS:K. Miyazawa 1163* | LC913215 | / | (Miyazawa and Ohmura 2026) |
| *Porina rufula* | China, *YN222406-4* (unpublished) | OR164482 | / | Unpublished |
| *Porina rufula* | China, *YN18341-1* (unpublished) | OR164485 | / | Unpublished |
| ***Porina rufula*** | **Mauritius is., *Ertz 23539D*** | **QB133698** | **QB133819** | Present study |
| ***Porina rufula*** | **Mayotte is., *Ertz 21938A*** | **QB133699** | **QB133817** | Present study |
| ***Porina rufula*** | **Mayotte is., *Ertz 21936A*** | **QB133700** | **QB133818** | Present study |
| *Porina rupicola* | Mauritius is., *Ertz 23620* | OK058505 | / | (Ertz and Diederich 2022) |
| *Porina rupicola* | Rodrigues is., *Ertz 23758* | OK058506 | / | (Ertz and Diederich 2022) |
| *Porina rupicola* | Mauritius, *Ertz 24088* | OK058507 | / | (Ertz and Diederich 2022) |
| *Porina simulans* | Dominican Republic, *Kalb 568* | DQ168379 | / | (Baloch and Grube 2006) |
| *Porina sorediata* | Brazil, *Caceres and Aptroot 42451* | / | MK080109 | Unpublished |
| *Porina* sp. | Panama, *Lücking Pan-Comp. PAN-19* | KJ449317 | / | (Nelsen et al. 2014) |
| *Porina* sp. | China, *YN18152* (unpublished) | OR164487 | / | Unpublished |
| *Porina* sp. | China, *FJ220224-1* (unpublished) | OR164488 | / | Unpublished |
| *Porina* sp. | China, *FJ220223* (unpublished) | OR164489 | / | Unpublished |
| *Porina* sp. | China, *FJ220140* (unpublished) | OR164490 | / | Unpublished |
| *Porina* sp. | China, *FJ220412* (unpublished) | OR164491 | / | Unpublished |
| *Porina* sp. | China, *FJ220410* (unpublished) | OR164492 | / | Unpublished |
| *Porina* sp. | China, *YN18021* (unpublished) | OR164493 | / | Unpublished |
| *Porina* sp. | United Kingdom, *Orange 20628* | / | KR108901 | (Orange 2015) |
| *Porina* sp. | United Kingdom, *Orange 24121* | / | MN687927 | (Orange et al. 2020) |
| *Porina* sp. | United Kingdom, *Orange 24128* | / | MN687928 | (Orange et al. 2020) |
| *Porina* sp. | United Kingdom, *Orange 24073* | / | MN687929 | (Orange et al. 2020) |
| *Porina* sp. | United Kingdom, *Orange 24180* | / | MN687930 | (Orange et al. 2020) |
| *Porina* sp. | United Kingdom, *Orange 20644* | KR108914 | KR108900 | (Orange 2015) |
| *Porina* sp. | Canada, *Orange 22778* | KT230860 | / | (Orange 2015) |
| *Porina* sp. | Canada, *Orange 22778* | KT230860 | / | (Orange 2015) |
| *Porina* sp. | Mauritius is., *Ertz 23584* | OK058508 | / | (Ertz and Diederich 2022) |
| *Porina* sp. | Mauritius is., *Ertz 23618* | OK058509 | / | (Ertz and Diederich 2022) |
| *Porina* sp. | China, *YN210912* (unpublished) | OM219178 | / | Unpublished |
| *Porina* sp. | China, *YN210920-1* (unpublished) | OM219179 | / | Unpublished |
| *Porina* sp. | Florida, *WBS 20509.4* | OR036936 | / | (Sanders et al. 2023) |
| ***Porina* sp. 1** | **Madagascar, *Ertz 25411E*** | **QB133767** | **/** | Present study |
| ***Porina* sp. 2** | **Madagascar, *Ertz 20279I*** | **QB133772** | **QB133836** | Present study |
| ***Porina* sp. 3** | **Madagascar, *Ertz 25411D*** | **QB133776** | **QB133845** | Present study |
| ***Porina* sp. 4** | **Madagascar, *Ertz 20275C*** | **QB133775** | **QB133843** | Present study |
| ***Porina* sp. 5** | **Madagascar, *Ertz 19816B*** | **/** | **QB133813** | Present study |
| ***Porina sphaerocephala*** | **Madagascar, *Ertz 25329C*** | **QB133714** | **/** | Present study |
| ***Porina sphaerocephala*** | **Madagascar, *Ertz 25411C*** | **QB133715** | **QB133802** | Present study |
| ***Porina sphaerocephala*** | **Madagascar, *Ertz 25321D*** | **QB133716** | **QB133801** | Present study |
| ***Porina sphaerocephaloides*** | **Madagascar, *Ertz 27163C*** | **QB133701** | **QB133803** | Present study |
| ***Porina sphaerocephaloides*** | **Madagascar, *Ertz 20274E*** | **QB133702** | **QB133805** | Present study |
| ***Porina sphaerocephaloides*** | **Madagascar, *Ertz 20270F*** | **QB133703** | **QB133806** | Present study |
| ***Porina sphaerocephaloides*** | **Madagascar, *Ertz 20279L*** | **QB133704** | **QB133804** | Present study |
| ***Porina sphaerocephaloides*** | **Madagascar, *Ertz 20279Q*** | **QB133705** | **/** | Present study |
| ***Porina sphaerocephaloides*** | **Madagascar, *Ertz 20275B*** | **QB133706** | **/** | Present study |
| ***Porina sphaerocephaloides*** | **Madagascar, *Ertz 20280D*** | **QB133707** | **QB133811** | Present study |
| ***Porina sphaerocephaloides*** | **Madagascar, *Ertz 20272H*** | **QB133708** | **QB133812** | Present study |
| ***Porina sphaerocephaloides*** | **Madagascar, *Ertz 20277G*** | **QB133709** | **/** | Present study |
| ***Porina sphaerocephaloides*** | **Madagascar, *Ertz 25373B*** | **QB133710** | **QB133810** | Present study |
| ***Porina sphaerocephaloides*** | **Madagascar, *Ertz 25375G*** | **QB133711** | **QB133807** | Present study |
| ***Porina sphaerocephaloides*** | **Madagascar, *Ertz 20272G*** | **QB133712** | **QB133809** | Present study |
| ***Porina sphaerocephaloides*** | **Madagascar, *Ertz 20279M*** | **QB133713** | **QB133808** | Present study |
| *Porina subepiphylla* | Costa Rica, *Baloch CR160/1-F145* | DQ168380 | / | (Baloch and Grube 2006) |
| *Porina subepiphylla* | Costa Rica, *Baloch CR289-F517* | DQ168381 | / | (Baloch and Grube 2006) |
| *Porina subepiphylla* | Costa Rica, *Baloch CR1023-F1602* | FJ711145 | / | (Baloch and Grube 2009) |
| *Porina subepiphylla* | China, *YN18332-2* (unpublished) | OM219167 | / | Unpublished |
| *Porina subepiphylla* | China, *YN210729* (unpublished) | OM219168 | / | Unpublished |
| *Porina subepiphylla* | China, *YN222518* (unpublished) | OR164507 | / | Unpublished |
| *Porina subinterstes* | China, *YN18332-1* (unpublished) | OM219171 | / | Unpublished |
| *Porina subinterstes* | China, *YN222517-2* (unpublished) | OR164508 | / | Unpublished |
| *Porina subnitidula* | Mexico, *Herrera-Campos S39P8a-F1054* | DQ168394 | / | (Baloch and Grube 2006) |
| *Porina subnitidula* | China, *FJ220425-1* (unpublished) | OQ383626 | / | Unpublished |
| *Porina tetracerae* | Costa Rica, *Lücking 17038b* | KJ449309 | / | (Nelsen et al. 2014) |
| *Porina tetracerae* | Panama, *Lücking Panama-Cupanella, Pan-07* | KJ449314 | / | (Nelsen et al. 2014) |
| ***Porina trichocoronata*** | **Madagascar, *Ertz 20270K*** | **/** | **QB133820** | Present study |
| *Porina virescens* | China, *YN18286-2* (unpublished) | MT526255 | / | Unpublished |
| *Porina virescens* | China, *YN18292* (unpublished) | MT526258 | / | Unpublished |
| *Porina virescens* | China, *YN210924-2* (unpublished) | OM219170 | / | Unpublished |
| *Porina virescens* | China, *YN210921-3* (unpublished) | OR164509 | / | Unpublished |
| *Saxiloba firmula* | Cuba, *Lücking et al. 41891d* | MT371252 | / | Unpublished |
| ***Trichothelium* aff. *argenteum*** | **Madagascar, *Ertz 20277C*** | **/** | **QB133822** | Present study |
| *Trichothelium annulatum* | Mexico, *Herrera-Campos S39P3-F1057* | DQ168415 | / | (Baloch and Grube 2006) |
| Trichothelium assurgens | Japan, *TNS:K. Miyazawa 766E* | LC913239 | / | (Miyazawa and Ohmura 2026) |
| ***Trichothelium* cf. *epiphyllum*** | **Madagascar, *Ertz 20279J*** | **/** | **QB133826** | Present study |
| ***Trichothelium* cf. *epiphyllum*** | **Madagascar, *Ertz 25340B*** | **/** | **QB133827** | Present study |
| ***Trichothelium* cf. *epiphyllum*** | **Madagascar, *Ertz 25409B*** | **/** | **QB133828** | Present study |
| ***Trichothelium* cf. *epiphyllum*** | **Madagascar, *Ertz 20272I*** | **QB133718** | **QB133824** | Present study |
| ***Trichothelium* cf. *epiphyllum*** | **Madagascar, *Ertz 20271F*** | **QB133719** | **QB133825** | Present study |
| ***Trichothelium* cf. *epiphyllum*** | **Madagascar, *Ertz 20279K*** | **QB133720** | **QB133830** | Present study |
| ***Trichothelium* cf. *epiphyllum*** | **Madagascar, *Ertz 20279F*** | **QB133721** | **QB133829** | Present study |
| *Trichothelium epiphyllum* | Costa Rica, *Baloch CR127* | AY648901 | / | (Grube et al. 2004) |
| *Trichothelium epiphyllum* | Costa Rica, *Baloch CR732/1-F1431* | DQ168416 | / | (Baloch and Grube 2006) |
| *Trichothelium pallidisetum* | Costa Rica, *Lücking 16030* | AY648900 | / | (Grube et al. 2004) |
| ***Trichothelium* sp.** | **Madagascar, *Ertz 20279G*** | **QB133722** | **QB133823** | Present study |

**References**

Baloch E, Grube M (2006) Evolution and phylogenetic relationships within *Porinaceae* (*Ostropomycetidae*), focusing on foliicolous species. Mycological Research 110: 125–136. https://doi.org/10.1016/j.mycres.2005.09.009

Baloch E, Grube M (2009) Pronounced genetic diversity in tropical epiphyllous li­chen fungi. Molecular Ecology 18: 2185–2197. https://doi.org/10.1111/j.1365- 294x.2009.04183.x

Diederich P, Ertz D (2020) First checklist of lichens and lichenicolous fungi from Mauri­tius, with phylogenetic analyses and description of new taxa. Plant and Fungal Sys­tematics 65: 13–75. https://doi.org/10.35535/pfsyst-2020-0003

Ertz D (2023) Les lichens et les champignons lichénicoles de la vallée de la Semois en­tre Bouillon et Bohan, avec dix-neuf espèces nouvelles pour la Belgique. Dumortiera 121: 18–44. https://doi.org/10.5281/zenodo.7830895

Ertz D (2024) Quatre espèces de lichens et deux espèces de champignons lichéni­coles nouvelles pour la Belgique, et d’autres espèces intéressantes pour le pays. Du­mortiera 124: 28–33. https://doi.org/10.5281/zenodo.14186218

Ertz D, Diederich P (2022) Unravelling the diversity of the lichen genus *Porina* (*Porinace­ae*) in Mauritius. Plant Ecology and Evolution 155: 123–152. https://doi.org/10.5091/ plecevo.84545

Ertz D, Sanderson N, Coppins BJ et al. (2019) *Opegrapha multipuncta* and *Schismatom­ma quercicola* (*Arthoniomycetes*) belong to the *Lecanoromycetes*. The Lichenologist 51: 395–405. https://doi.org/10.1017/s002428291900029x

Ertz D, Sanderson N, Lebouvier M (2021) *Thelopsis* challenges the generic circumscrip­tion in the *Gyalectaceae* and brings new insights to the taxonomy of *Ramonia*. The Lichenologist 53: 45–61. https://doi.org/10.1017/s002428292000050x

Grube M, Baloch E, Lumbsch HT (2004) The phylogeny of *Porinaceae* (*Ostropomyceti­dae*) suggests a neotenic origin of perithecia in *Lecanoromycetes*. Mycological Re­search 108: 1111–1118. https://doi.org/10.1017/s0953756204000826

Lebreton E, Ertz D, Lücking R et al. (2024) Further expansion of morphological vari­ability in the *Porinaceae* (*Ascomycota*, *Ostropales*) with the placement of the enig­matic genus *Gallaicolichen*. The Lichenologist 56: 61–71. https://doi.org/10.1017/ s0024282924000124

Lumbsch HT, Schmitt I, Palice Z et al. (2003) Supraordinal phylogenetic relationships of *Lecanoromycetes* based on a Bayesian analysis of combined nuclear and mito­chondrial sequences. Molecular Phylogenetics and Evolution 31: 822–832. https:// doi.org/10.1016/j.ympev.2003.11.001

Lutzoni F, Kauff F, Cox CJ et al. (2004) Assembling the fungal tree of life: progress, clas­sification, and evolution of subcellular traits. American Journal of Botany 91: 1446– 1480. <https://doi.org/10.3732/ajb.91.10.1446>

Malíček J, Coppins B, Palice Z et al. (2023) *Coenogonium nimisii* – a new isidiate ep­iphytic lichen similar to *Porina rosei*. The Lichenologist 55: 305–313. https://doi. org/10.1017/s0024282923000257

Miadlikowska J, Kauff F, Högnabba F et al. (2014) A multigene phylogenetic synthesis for the class *Lecanoromycetes* (*Ascomycota*): 1307 fungi representing 1139 infrage­neric taxa, 317 genera and 66 families. Molecular Phylogenetics and Evolution 79: 132–168. <https://doi.org/10.1016/j.ympev.2014.04.003>

Miyazawa K, Ohmura Y (2026) Noteworthy foliicolous lichens and a lichenicolous fun­gus collected from Okinawa Island, southern Japan. Taiwania 71: 159–184. https:// doi.org/10.6165/tai.2026.71.159

Miyazawa K, Ohmura Y, Yamaoka Y (2022) Noteworthy foliicolous lichens collected from Iriomote Island, southern Japan. Taiwania 67: 155–163. https://doi.org/10.6165/ tai.2022.67.155

Nelsen MP, Lücking R, Andrew CJ et al. (2014) Molecular phylogeny reveals the true colours of *Myeloconidaceae* (*Ascomycota*: *Ostropales*). Australian Systematic Botany 27: 38–47. https://doi.org/10.1071/sb13040

Orange A (2015) A new freshwater *Porina* (*Porinaceae*, *Ostropales*) from Great Britain. The Lichenologist 47: 351–358. https://doi.org/10.1017/s0024282915000365

Orange A, Palice Z, Klepsland J (2020) A new isidiate saxicolous species of *Porina* (*Ascomycota*, *Ostropales*, *Porinaceae*). The Lichenologist 52: 267–277. https://doi. org/10.1017/s0024282920000183

Sanders W, De Carolis R, Ertz D et al. (2023) Independent, structurally distinct transi­tions to microfruticose growth in the crustose genus *Porina* (*Ostropales*, *Lecanoro­mycetes*): new isidioid species from south-western Florida. The Lichenologist 55: 347–365. https://doi.org/10.1017/s0024282923000440

Sobreira PNB, Cáceres MES, Maia LC et al. (2018) *Flabelloporina*, a new genus in the *Por­inaceae* (*Ascomycota*, *Ostropales*), with the first record of *F. squamulifera* from Brazil. Phytotaxa 358: 67–75. https://doi.org/10.11646/phytotaxa.358.1.4

Vondrák J, Svoboda S, Košnar J et al. (2023) Martin7: A reference database of DNA barcodes for European epiphytic lichens and its taxonomic implications. Preslia 95: 311–345. https://doi.org/10.23855/preslia.2023.311
