# Supplementary material for: Integrative taxonomy reveals hidden fungal and algal diversity in foliicolous Porinaceae (Ascomycota, Ostropales) from Madagascar and the Indian Ocean Islands
Source: IMA Fungus. 2026 Nov 5;17:e207656. doi: 10.3897/imafungus.17.207656 (PMC13760347; doi:10.3897/imafungus.17.207656)
Supplement: Supplementary file 5 [file imafungus-17-e207656-s002.docx]

**Table 2 –** Origin of the alga (host or free living), algal species, GenBank Accession numbers of *rbc*L sequences used in this study, and associated reference. Newly generated sequences are in bold.

| **Origin of alga** | **Algal species** | **Genbank number** | **Reference** |
| --- | --- | --- | --- |
| *Acanthothecis* sp*.* | *Trentepohliales* | GU549462 | (Nelsen et al. 2011) |
| *Acrocordia gemmata* | *Trentepohlia* sp. | JQ617931 | (Hametner et al. 2014a) |
| *Allographa* cf*. cinerea* | *Printzina lagenifera* | KC469275 | (Hametner et al. 2014b) |
| *Allographa laubertiana* | *Trentepohlia* sp. | KC469249 | (Hametner et al. 2014b) |
| *Allographa subruiziana* | *Printzina* sp*.* | KC469237 | (Hametner et al. 2014b) |
| *Alyxoria ochrocincta* | *Trentepohliaceae* sp. | OL956922 | (Borgato et al. 2022) |
| *Alyxoria* sp*.* | *Trentepohlia* cf*. jolithus* | MT075944 | (Kosecka et al. 2020) |
| *Alyxoria varia* | *Trentepohlia abietina* | KC469256 | (Hametner et al. 2014b) |
| *Alyxoria varia* | *Trentepohliaceae* sp. | OL956835 | (Borgato et al. 2022) |
| *Alyxoria varia* | *Trentepohliaceae* sp. | OL956837 | (Borgato et al. 2022) |
| *Anthracothecium cristatellum* | *Trentepohliales* | GU549443 | (Nelsen et al. 2011) |
| *Arthonia atra* | *Trentepohlia* sp. | JQ617933 | (Hametner et al. 2014a) |
| *Arthonia atra* | *Trentepohlia* sp. | JQ617934 | (Hametner et al. 2014a) |
| *Arthonia atra* | *Trentepohliaceae* sp. | OL956813 | (Borgato et al. 2022) |
| *Arthonia atra* | *Trentepohliaceae* sp. | OL956830 | (Borgato et al. 2022) |
| *Arthonia atra* | *Trentepohliaceae* sp. | OL956834 | (Borgato et al. 2022) |
| *Arthonia atra* | *Trentepohliaceae* sp. | OL956839 | (Borgato et al. 2022) |
| *Arthonia atra* | *Trentepohliaceae* sp. | OL956842 | (Borgato et al. 2022) |
| *Arthonia atra* | *Trentepohliaceae* sp. | OL956864 | (Borgato et al. 2022) |
| *Arthonia atra* | *Trentepohliaceae* sp. | OL956924 | (Borgato et al. 2022) |
| *Arthonia atra* | *Trentepohliaceae* sp. | OL956926 | (Borgato et al. 2022) |
| *Arthonia atra* | *Trentepohliaceae* sp. | OL956927 | (Borgato et al. 2022) |
| *Arthonia* cf*. pyrrhuliza* | *Trentepohlia* sp. | KC469261 | (Hametner et al. 2014b) |
| *Arthonia didyma* | *Trentepohliaceae* sp. | OL956844 | (Borgato et al. 2022) |
| *Arthonia gr calcarea* | *Trentepohliaceae* sp. | OL956901 | (Borgato et al. 2022) |
| *Arthonia meridionalis* | *Trentepohliaceae* sp. | OL956899 | (Borgato et al. 2022) |
| *Arthonia radiata* | *Trentepohlia* sp. | JQ617929 | (Hametner et al. 2014a) |
| *Arthonia radiata* | *Trentepohliaceae* sp. | OL956868 | (Borgato et al. 2022) |
| *Arthonia ruana* | *Printzina* cf*. lagenifera* | JQ617914 | (Hametner et al. 2014a) |
| *Arthonia ruana* | *Trentepohliaceae* sp. | OL956865 | (Borgato et al. 2022) |
| *Arthonia* sp*.* | *Printzina* sp*.* | KC469272 | (Hametner et al. 2014b) |
| *Astrochapsa* sp*.* | *Trentepohlia* sp. | MT075946 | (Kosecka et al. 2020) |
| *Astrothelium aeneum* | *Trentepohliales* | GU549451 | (Nelsen et al. 2011) |
| *Astrothelium aeneum* | *Trentepohliales* | GU549467 | (Nelsen et al. 2011) |
| *Astrothelium* cf*. aeneum* | *Trentepohliales* | GU549448 | (Nelsen et al. 2011) |
| *Astrothelium* cf*. diplocarpum* | *Trentepohliales* | GU549468 | (Nelsen et al. 2011) |
| *Astrothelium cinnamomeum* | *Trentepohliales* | GU549449 | (Nelsen et al. 2011) |
| *Astrothelium galbineum* | *Trentepohliales* | GU549471 | (Nelsen et al. 2011) |
| *Astrothelium leucoconicum* | *Trentepohliales* | GU549447 | (Nelsen et al. 2011) |
| *Astrothelium megasp.ermum* | *Trentepohliales* | GU549442 | (Nelsen et al. 2011) |
| *Astrothelium megasp.ermum* | *Trentepohliales* | GU549470 | (Nelsen et al. 2011) |
| *Astrothelium nitidiusculum* | *Trentepohliales* | GU549469 | (Nelsen et al. 2011) |
| *Astrothelium versicolor* | *Trentepohliales* | GU549450 | (Nelsen et al. 2011) |
| *Coenogonium* aff*. luteolum* | *Trentepohlia* sp. | KC469226 | (Hametner et al. 2014b) |
| *Coenogonium barbatum* | *Trentepohlia arborum* | KC469224 | (Hametner et al. 2014b) |
| *Coenogonium* cf*. stramineum* | *Printzina* sp*.* | KC469228 | (Hametner et al. 2014b) |
| *Coenogonium flavoviride* | *Printzina* sp*.* | KC469225 | (Hametner et al. 2014b) |
| *Coenogonium interpositum* | *Trentepohlia arborum* | KC469231 | (Hametner et al. 2014b) |
| *Coenogonium leprieurii* | *Printzina* sp*.* | KC469222 | (Hametner et al. 2014b) |
| *Coenogonium leprieurii* | *Trentepohlia* sp. | KC469227 | (Hametner et al. 2014b) |
| *Coenogonium linkii* | *Trentepohliales* | GU549472 | (Nelsen et al. 2011) |
| *Coenogonium linkii* | *Trentepohlia* sp. | KC469221 | (Hametner et al. 2014b) |
| *Coenogonium linkii* | *Trentepohlia* sp. | KC469232 | (Hametner et al. 2014b) |
| *Coenogonium linkii* | *Trentepohlia aurea* | KC469233 | (Hametner et al. 2014b) |
| *Coenogonium magdalenae* | *Printzina* sp*.* | KC469240 | (Hametner et al. 2014b) |
| *Coenogonium* sp*.* | *Trentepohlia* sp. | KC469203 | (Hametner et al. 2014b) |
| *Coenogonium* sp*.* | *Trentepohlia arborum* | KC469223 | (Hametner et al. 2014b) |
| *Coenogonium* sp*.* | *Printzina lagenifera* | MT075947 | (Kosecka et al. 2020) |
| *Coenogonium subdilutum* | *Printzina* sp*.* | KC469230 | (Hametner et al. 2014b) |
| *Coenogonium subluteum* | *Trentepohlia* sp. | KC469229 | (Hametner et al. 2014b) |
| *Coniocarpon cinnabarinum* | *Printzina lagenifera* | JQ617932 | (Hametner et al. 2014a) |
| *Coniocarpon cinnabarinum* | *Printzina* cf*. lagenifera* | JQ617944 | (Hametner et al. 2014a) |
| *Coniocarpon cinnabarinum* | *Printzina* sp*.* | KC469214 | (Hametner et al. 2014b) |
| *Coniocarpon cinnabarinum* | *Trentepohliaceae* sp. | OL956816 | (Borgato et al. 2022) |
| *Coniocarpon cinnabarinum* | *Trentepohliaceae* sp. | OL956818 | (Borgato et al. 2022) |
| *Coniocarpon cinnabarinum* | *Trentepohliaceae* sp. | OL956826 | (Borgato et al. 2022) |
| *Coniocarpon cinnabarinum* | *Trentepohliaceae* sp. | OL956827 | (Borgato et al. 2022) |
| *Coniocarpon cinnabarinum* | *Trentepohliaceae* sp. | OL956841 | (Borgato et al. 2022) |
| *Coniocarpon cinnabarinum* | *Trentepohliaceae* sp. | OL956869 | (Borgato et al. 2022) |
| *Coniocarpon cinnabarinum* | *Trentepohliaceae* sp. | OL956870 | (Borgato et al. 2022) |
| *Cryptothecia assimilis* | *Trentepohliales* | GU549460 | (Nelsen et al. 2011) |
| *Cryptothelium* sp*.* | *Trentepohliales* | GU549452 | (Nelsen et al. 2011) |
| *Cystocoleus ebeneus* | *Trentepohlia* sp. | JQ617917 | (Hametner et al. 2014a) |
| *Dendrographa alectoroides* | *Trentepohliales* | GU549441 | (Nelsen et al. 2011) |
| *Dendrographa decolorans* | *Trentepohliaceae* sp. | OL956836 | (Borgato et al. 2022) |
| *Dendrographa decolorans* | *Trentepohliaceae* sp. | OL956884 | (Borgato et al. 2022) |
| *Dendrographa decolorans* | *Trentepohliaceae* sp. | OL956930 | (Borgato et al. 2022) |
| *Dendrographa latebrarum* | *Trentepohliaceae* sp. | OL956822 | (Borgato et al. 2022) |
| *Dendrographa latebrarum* | *Trentepohliaceae* sp. | OL956889 | (Borgato et al. 2022) |
| *Diarthonis spadicea* | *Trentepohliaceae* sp. | OL956853 | (Borgato et al. 2022) |
| *Diarthonis spadicea* | *Trentepohliaceae* sp. | OL956860 | (Borgato et al. 2022) |
| *Diarthonis spadicea* | *Trentepohliaceae* sp. | OL956862 | (Borgato et al. 2022) |
| *Dichosporidium boschianum* | *Trentepohliales* | GU549461 | (Nelsen et al. 2011) |
| *Dichosporidium nigrocinctum* | *Printzina* cf*. bosseae* | MT075948 | (Kosecka et al. 2020) |
| *Dichosporidium nigrocinctum* | *Printzina* cf*. bosseae* | MT075949 | (Kosecka et al. 2020) |
| *Dichosporidium nigrocinctum* | *Printzina* cf*. bosseae* | MT075950 | (Kosecka et al. 2020) |
| *Dichosporidium nigrocinctum* | *Printzina* cf*. bosseae* | MT075951 | (Kosecka et al. 2020) |
| *Dichosporidium nigrocinctum* | *Printzina* cf*. bosseae* | MT075952 | (Kosecka et al. 2020) |
| *Dichosporidium nigrocinctum* | *Printzina* cf*. bosseae* | MT075954 | (Kosecka et al. 2020) |
| *Dichosporidium nigrocinctum* | *Trentepohlia* sp. | MT075955 | (Kosecka et al. 2020) |
| *Dichosporidium nigrocinctum* | *Printzina bosseae* | MT075956 | (Kosecka et al. 2020) |
| *Dichosporidium nigrocinctum* | *Printzina bosseae* | MT075957 | (Kosecka et al. 2020) |
| *Dichosporidium nigrocinctum* | *Printzina bosseae* | MT076039 | (Kosecka et al. 2020) |
| *Dichosporidium nigrocinctum* | *Printzina bosseae* | MT076040 | (Kosecka et al. 2020) |
| *Dichosporidium nigrocinctum* | *Printzina bosseae* | MT076041 | (Kosecka et al. 2020) |
| *Dichosporidium nigrocinctum* | *Printzina bosseae* | MT076042 | (Kosecka et al. 2020) |
| *Dichosporidium nigrocinctum* | *Trentepohliaceae* sp. | OL956906 | (Borgato et al. 2022) |
| *Dichosporidium nigrocinctum* | *Trentepohliaceae* sp. | OL956909 | (Borgato et al. 2022) |
| *Diorygma antillarum* | *Printzina lagenifera* | MT075958 | (Kosecka et al. 2020) |
| *Diorygma antillarum* | *Printzina lagenifera* | MT075959 | (Kosecka et al. 2020) |
| *Diorygma antillarum* | *Printzina lagenifera* | MT075960 | (Kosecka et al. 2020) |
| *Diorygma antillarum* | *Printzina lagenifera* | MT075961 | (Kosecka et al. 2020) |
| *Diorygma antillarum* | *Printzina lagenifera* | MT075962 | (Kosecka et al. 2020) |
| *Diorygma antillarum* | *Printzina lagenifera* | MT075963 | (Kosecka et al. 2020) |
| *Diorygma antillarum* | *Printzina lagenifera* | MT075964 | (Kosecka et al. 2020) |
| *Diorygma antillarum* | *Printzina lagenifera* | MT075965 | (Kosecka et al. 2020) |
| *Diorygma antillarum* | *Printzina lagenifera* | MT075966 | (Kosecka et al. 2020) |
| *Diorygma antillarum* | *Printzina lagenifera* | MT075967 | (Kosecka et al. 2020) |
| *Diorygma antillarum* | *Printzina lagenifera* | MT075968 | (Kosecka et al. 2020) |
| *Diorygma confluens* | *Trentepohlia* sp. | MT075969 | (Kosecka et al. 2020) |
| *Diorygma pruinosum* | *Trentepohlia* sp. | KC469219 | (Hametner et al. 2014b) |
| *Diorygma* sp*.* | *Trentepohlia* sp. | MT075970 | (Kosecka et al. 2020) |
| *Diorygma* sp*.* | *Printzina* sp*.* | MT075971 | (Kosecka et al. 2020) |
| *Diorygma* sp*.* | *Printzina* sp*.* | MT075972 | (Kosecka et al. 2020) |
| *Diorygma* sp*.* | *Printzina* sp*.* | MT075973 | (Kosecka et al. 2020) |
| *Diorygma* sp*.* | *Trentepohlia* sp. | MT075974 | (Kosecka et al. 2020) |
| *Diorygma* sp*.* | *Trentepohlia* sp. | MT075975 | (Kosecka et al. 2020) |
| *Diorygma* sp*.* | *Printzina* sp*.* | MT075976 | (Kosecka et al. 2020) |
| *Diorygma* sp*.* | *Printzina lagenifera* | MT075977 | (Kosecka et al. 2020) |
| *Dirina ceratoniae* | *Trentepohliaceae* sp. | OL956898 | (Borgato et al. 2022) |
| *Dirina fallax* | *Trentepohliaceae* sp. | OL956894 | (Borgato et al. 2022) |
| *Dirina fallax* | *Trentepohliaceae* sp. | OL956895 | (Borgato et al. 2022) |
| *Dirina fallax* | *Trentepohliaceae* sp. | OL956896 | (Borgato et al. 2022) |
| *Dirina fallax* | *Trentepohliaceae* sp. | OL956911 | (Borgato et al. 2022) |
| *Dirina fallax* | *Trentepohliaceae* sp. | OL956914 | (Borgato et al. 2022) |
| *Dirina fallax* | *Trentepohliaceae* sp. | OL956919 | (Borgato et al. 2022) |
| *Dirina massiliensis* | *Trentepohliaceae* sp. | OL956812 | (Borgato et al. 2022) |
| *Dirina massiliensis* | *Trentepohliaceae* sp. | OL956931 | (Borgato et al. 2022) |
| *Diromma dirinellum* | *Trentepohliaceae* sp. | OL956885 | (Borgato et al. 2022) |
| *Enterographa cf quassiaecola* | *Trentepohliaceae* sp. | OL956907 | (Borgato et al. 2022) |
| *Enterographa crassa* | *Trentepohliaceae* sp. | OL956846 | (Borgato et al. 2022) |
| *Enterographa crassa* | *Trentepohliaceae* sp. | OL956847 | (Borgato et al. 2022) |
| *Enterographa crassa* | *Trentepohliaceae* sp. | OL956850 | (Borgato et al. 2022) |
| *Enterographa crassa* | *Trentepohliaceae* sp. | OL956854 | (Borgato et al. 2022) |
| *Enterographa crassa* | *Trentepohliaceae* sp. | OL956856 | (Borgato et al. 2022) |
| *Enterographa crassa* | *Trentepohliaceae* sp. | OL956857 | (Borgato et al. 2022) |
| *Enterographa crassa* | *Trentepohliaceae* sp. | OL956859 | (Borgato et al. 2022) |
| *Enterographa crassa* | *Trentepohliaceae* sp. | OL956873 | (Borgato et al. 2022) |
| *Enterographa crassa* | *Trentepohliaceae* sp. | OL956876 | (Borgato et al. 2022) |
| *Enterographa crassa* | *Trentepohliaceae* sp. | OL956877 | (Borgato et al. 2022) |
| *Enterographa crassa* | *Trentepohliaceae* sp. | OL956878 | (Borgato et al. 2022) |
| *Enterographa crassa* | *Trentepohliaceae* sp. | OL956882 | (Borgato et al. 2022) |
| *Enterographa hutchinsiae* | *Trentepohliaceae* sp. | OL956823 | (Borgato et al. 2022) |
| *Enterographa hutchinsiae* | *Trentepohliaceae* sp. | OL956824 | (Borgato et al. 2022) |
| *Enterographa pitardii* | *Trentepohliaceae* sp. | OL956887 | (Borgato et al. 2022) |
| *Enterographa zonata* | *Trentepohliaceae* sp. | OL956825 | (Borgato et al. 2022) |
| Free living | *Ulva linza* | AB097620 | (Shimada et al. 2003) |
| Free living | *Caulerpa prolifera* | AY942173 | (Lam and Zechman 2006) |
| Free living | *Halimeda opuntia* | AY942174 | (Lam and Zechman 2006) |
| Free living | *Cephaleuros parasiticus* | FJ534596 | (Rindi et al. 2009) |
| Free living | *Cephaleuros virescens* | FJ534597 | (Rindi et al. 2009) |
| Free living | *Cephaleuros virescens* | FJ534598 | (Rindi et al. 2009) |
| Free living | *Cephaleuros virescens* | FJ534599 | (Rindi et al. 2009) |
| Free living | *Cephaleuros virescens* | FJ534600 | (Rindi et al. 2009) |
| Free living | *Cephaleuros virescens* | FJ534601 | (Rindi et al. 2009) |
| Free living | *Trentepohlia* cf*. jolithus* | FJ534602 | (Rindi et al. 2009) |
| Free living | *Trentepohlia* cf*. abietina* | FJ534603 | (Rindi et al. 2009) |
| Free living | *Trentepohlia arborum* | FJ534604 | (Rindi et al. 2009) |
| Free living | *Trentepohlia arborum* | FJ534605 | (Rindi et al. 2009) |
| Free living | *Trentepohlia arborum* | FJ534606 | (Rindi et al. 2009) |
| Free living | *Trentepohlia arborum* | FJ534607 | (Rindi et al. 2009) |
| Free living | *Trentepohlia aurea* | FJ534608 | (Rindi et al. 2009) |
| Free living | *Trentepohlia aurea* | FJ534609 | (Rindi et al. 2009) |
| Free living | *Trentepohlia* cf*. annulata* | FJ534610 | (Rindi et al. 2009) |
| Free living | *Trentepohlia* cf*. flintii* | FJ534611 | (Rindi et al. 2009) |
| Free living | *Trentepohlia* cf*. umbrina* | FJ534612 | (Rindi et al. 2009) |
| Free living | *Trentepohlia dialepta* | FJ534613 | (Rindi et al. 2009) |
| Free living | *Trentepohlia dusenii* | FJ534614 | (Rindi et al. 2009) |
| Free living | *Trentepohlia flava* | FJ534615 | (Rindi et al. 2009) |
| Free living | *Trentepohlia flava* | FJ534616 | (Rindi et al. 2009) |
| Free living | *Trentepohlia* sp. | FJ534617 | (Rindi et al. 2009) |
| Free living | *Trentepohlia* sp. | FJ534618 | (Rindi et al. 2009) |
| Free living | *Trentepohlia* sp. | FJ534619 | (Rindi et al. 2009) |
| Free living | *Trentepohlia jolithus* | FJ534620 | (Rindi et al. 2009) |
| Free living | *Trentepohlia umbrina* | FJ534621 | (Rindi et al. 2009) |
| Free living | *Trentepohlia umbrina* | FJ534622 | (Rindi et al. 2009) |
| Free living | *Printzina* cf*. bosseae* | FJ534623 | (Rindi et al. 2009) |
| Free living | *Printzina bosseae* | FJ534624 | (Rindi et al. 2009) |
| Free living | *Printzina bosseae* | FJ534625 | (Rindi et al. 2009) |
| Free living | *Printzina bosseae* | FJ534626 | (Rindi et al. 2009) |
| Free living | *Printzina lagenifera* | FJ534627 | (Rindi et al. 2009) |
| Free living | *Printzina* cf*. lagenifera* | FJ534628 | (Rindi et al. 2009) |
| Free living | *Printzina lagenifera* | FJ534629 | (Rindi et al. 2009) |
| Free living | *Printzina* cf*. lagenifera* | FJ534630 | (Rindi et al. 2009) |
| Free living | *Printzina* cf*. lagenifera* | FJ534631 | (Rindi et al. 2009) |
| Free living | *Printzina lagenifera* | FJ534632 | (Rindi et al. 2009) |
| Free living | *Printzina effusa* | FJ534633 | (Rindi et al. 2009) |
| Free living | *Printzina* sp*.* | FJ534634 | (Rindi et al. 2009) |
| Free living | *Printzina* sp*.* | FJ534635 | (Rindi et al. 2009) |
| Free living | *Printzina* sp*.* | FJ534636 | (Rindi et al. 2009) |
| Free living | *Trentepohlia aurea* | FJ715722 | (Cocquyt et al. 2009) |
| Free living | *Trentepohlia* sp. | JQ617936 | (Hametner et al. 2014a) |
| Free living | *Trentepohlia* sp. | KC469265 | (Hametner et al. 2014b) |
| Free living | *Trentepohlia* sp. | KC469280 | (Hametner et al. 2014b) |
| Free living | *Cephaleuros parasiticus* | KC559111 | (Ramya et al. 2013) |
| Free living | *Printzina* sp*.* | KF019631 | (Hametner et al. 2014b) |
| Free living | *Trentepohlia aurea* | KF638562 | (Achankunju et Panikkar, Unpublished) |
| Free living | *Cephaleuros parasiticus* | KM464711 | (Fučíková et al. 2014) |
| Free living | *Trentepohlia annulata* | KM464712 | (Fučíková et al. 2014) |
| Free living | *Trentepohlia* sp. | KY098764 | (Lemes-Da-Silva et al. 2018) |
| Free living | *Trentepohlia* sp. | KY098765 | (Lemes-Da-Silva et al. 2018) |
| Free living | *Trentepohlia* sp. | KY098766 | (Lemes-Da-Silva et al. 2018) |
| Free living | *Trentepohlia abietina* | KY762286 | (Lemes-Da-Silva et al., Unpublished) |
| Free living | *Trentepohlia* sp. | KY777577 | (Lemes-Da-Silva et al., Unpublished) |
| Free living | *Trentepohlia* sp. | KY777578 | (Lemes-Da-Silva et al., Unpublished) |
| Free living | *Cephaleuros parasiticus* | MG252608 | (Ertz et al. 2018) |
| Free living | *Cephaleuros virescens* | MG252609 | (Ertz et al. 2018) |
| Free living | *Cephaleuros* sp. | MG721734 | (Fang et al. 2018) |
| Free living | *Trentepohlia abietina* | MH940255 | (Klimešová et al. 2019) |
| Free living | *Trentepohlia jolithus* | MH940256 | (Klimešová et al. 2019) |
| Free living | *Trentepohlia umbrina* | MH940257 | (Klimešová et al. 2019) |
| Free living | *Trentepohlia umbrina* | MH940258 | (Klimešová et al. 2019) |
| Free living | *Trentepohlia umbrina* | MH940260 | (Klimešová et al. 2019) |
| Free living | *Trentepohlia annulata* | MH940261 | (Klimešová et al. 2019) |
| Free living | *Trentepohlia annulata* | MH940262 | (Klimešová et al. 2019) |
| Free living | *Trentepohlia annulata* | MH940263 | (Klimešová et al. 2019) |
| Free living | *Trentepohlia annulata* | MH940264 | (Klimešová et al. 2019) |
| Free living | *Trentepohlia annulata* | MH940265 | (Klimešová et al. 2019) |
| Free living | *Trentepohlia annulata* | MH940266 | (Klimešová et al. 2019) |
| Free living | *Trentepohlia umbrina* | MH940267 | (Klimešová et al. 2019) |
| Free living | *Trentepohlia umbrina* | MH940268 | (Klimešová et al. 2019) |
| Free living | *Trentepohlia umbrina* | MH940269 | (Klimešová et al. 2019) |
| Free living | *Trentepohlia umbrina* | MH940270 | (Klimešová et al. 2019) |
| Free living | *Trentepohlia jolithus* | MH940271 | (Klimešová et al. 2019) |
| Free living | *Trentepohlia abietina* | MH940274 | (Klimešová et al. 2019) |
| Free living | *Trentepohlia umbrina* | MH940277 | (Klimešová et al. 2019) |
| Free living | *Trentepohlia umbrina* | MH940278 | (Klimešová et al. 2019) |
| Free living | *Trentepohlia umbrina* | MH940279 | (Klimešová et al. 2019) |
| Free living | *Trentepohlia umbrina* | MK281583 | (Bartoli et al. 2019) |
| Free living | *Trentepohlia aurea* | MN822087 | (Saini, Unpublished) |
| Free living | *Trentepohlia aurea* | MN822088 | (Saini, Unpublished) |
| Free living | *Trentepohlia aurea* | OL771308 | (Toppo et al., Unpublished) |
| Free living | *Trentepohliaceae* sp. | OL956811 | (Borgato et al. 2022) |
| *Glyphis cicatricosa* | *Trentepohlia* sp. | KC469235 | (Hametner et al. 2014b) |
| *Glyphis scyphulifera* | *Printzina lagenifera* | KC469273 | (Hametner et al. 2014b) |
| *Glyphis scyphulifera* | *Printzina lagenifera* | KC469274 | (Hametner et al. 2014b) |
| *Gorgadesia mira* | *Trentepohliaceae* sp. | OL956900 | (Borgato et al. 2022) |
| *Graphina polyclades* | *Printzina* sp*.* | KC469238 | (Hametner et al. 2014b) |
| *Graphis alboscripta* | *Printzina* sp*.* | KC469269 | (Hametner et al. 2014b) |
| *Graphis apertella* | *Printzina* sp*.* | KC469241 | (Hametner et al. 2014b) |
| *Graphis centrifuga* | *Printzina* sp*.* | KC469258 | (Hametner et al. 2014b) |
| *Graphis* cf*. pseudosophistica* | *Trentepohlia* sp. | KC469250 | (Hametner et al. 2014b) |
| *Graphis cincta* | *Printzina* sp*.* | KC469242 | (Hametner et al. 2014b) |
| *Graphis elegans* | *Printzina lagenifera* | KC469276 | (Hametner et al. 2014b) |
| *Graphis elegans* | *Trentepohliaceae* sp. | OL956880 | (Borgato et al. 2022) |
| *Graphis emersa* | *Trentepohlia* sp. | KC469206 | (Hametner et al. 2014b) |
| *Graphis inustuloides* | *Trentepohliaceae* sp. | OL956843 | (Borgato et al. 2022) |
| *Graphis inustuloides* | *Trentepohliaceae* sp. | OL956845 | (Borgato et al. 2022) |
| *Graphis leptocarpa* | *Printzina* sp*.* | KC469246 | (Hametner et al. 2014b) |
| *Graphis librata* | *Trentepohlia* sp. | KC469279 | (Hametner et al. 2014b) |
| *Graphis lineola* | *Printzina* sp*.* | KC469243 | (Hametner et al. 2014b) |
| *Graphis neoelongata* | *Trentepohlia* sp. | KC469217 | (Hametner et al. 2014b) |
| *Graphis propinqua* | *Trentepohlia* sp. | JF727818 | (Hametner et al. 2014a) |
| *Graphis scripta* | *Trentepohliales* | GU549473 | (Nelsen et al. 2011) |
| *Graphis scripta* | *Printzina lagenifera* | JF727813 | (Hametner et al. 2014a) |
| *Graphis scripta* | *Printzina lagenifera* | JF727816 | (Hametner et al. 2014a) |
| *Graphis scripta* | *Printzina* sp*.* | JQ617915 | (Hametner et al. 2014a) |
| *Graphis scripta* | *Printzina lagenifera* | JQ617937 | (Hametner et al. 2014a) |
| *Graphis scripta* | *Printzina lagenifera* | JQ617938 | (Hametner et al. 2014a) |
| *Graphis scripta* | *Printzina lagenifera* | JQ617939 | (Hametner et al. 2014a) |
| *Graphis scripta* | *Printzina lagenifera* | JQ617940 | (Hametner et al. 2014a) |
| *Graphis scripta* | *Printzina lagenifera* | JQ617941 | (Hametner et al. 2014a) |
| *Graphis scripta* | *Printzina lagenifera* | JQ617942 | (Hametner et al. 2014a) |
| *Graphis scripta* | *Printzina lagenifera* | JQ617943 | (Hametner et al. 2014a) |
| *Graphis scripta* | *Trentepohliaceae* sp. | OL956819 | (Borgato et al. 2022) |
| *Graphis scripta* | *Trentepohliaceae* sp. | OL956820 | (Borgato et al. 2022) |
| *Graphis scripta* | *Trentepohliaceae* sp. | OL956828 | (Borgato et al. 2022) |
| *Graphis scripta* | *Trentepohliaceae* sp. | OL956829 | (Borgato et al. 2022) |
| *Graphis scripta* | *Trentepohliaceae* sp. | OL956832 | (Borgato et al. 2022) |
| *Graphis scripta* | *Trentepohliaceae* sp. | OL956833 | (Borgato et al. 2022) |
| *Graphis scripta* | *Trentepohliaceae* sp. | OL956838 | (Borgato et al. 2022) |
| *Graphis scripta* | *Trentepohliaceae* sp. | OL956848 | (Borgato et al. 2022) |
| *Graphis scripta* | *Trentepohliaceae* sp. | OL956849 | (Borgato et al. 2022) |
| *Graphis scripta* | *Trentepohliaceae* sp. | OL956855 | (Borgato et al. 2022) |
| *Graphis scripta* | *Trentepohliaceae* sp. | OL956863 | (Borgato et al. 2022) |
| *Graphis scripta* | *Trentepohliaceae* sp. | OL956866 | (Borgato et al. 2022) |
| *Graphis scripta* | *Trentepohliaceae* sp. | OL956867 | (Borgato et al. 2022) |
| *Graphis scripta* | *Trentepohliaceae* sp. | OL956871 | (Borgato et al. 2022) |
| *Graphis* sp*.* | *Trentepohliales* | GU549466 | (Nelsen et al. 2011) |
| *Graphis* sp*.* | *Printzina lagenifera* | KC469208 | (Hametner et al. 2014b) |
| *Graphis* sp*.* | *Printzina lagenifera* | KC469209 | (Hametner et al. 2014b) |
| *Graphis* sp*.* | *Printzina lagenifera* | KC469210 | (Hametner et al. 2014b) |
| *Graphis* sp*.* | *Trentepohlia* sp. | KC469212 | (Hametner et al. 2014b) |
| *Graphis* sp*.* | *Trentepohlia* sp. | KC469218 | (Hametner et al. 2014b) |
| *Graphis* sp*.* | *Printzina* sp*.* | KC469239 | (Hametner et al. 2014b) |
| *Graphis* sp*.* | *Trentepohlia* sp. | KC469260 | (Hametner et al. 2014b) |
| *Graphis* sp*.* | *Trentepohlia* sp. | KF031493 | (Hametner et al. 2014b) |
| *Graphis submarginata* | *Printzina* sp*.* | JF727821 | (Hametner et al. 2014a) |
| *Graphis virescens* | *Trentepohlia* sp. | KC469205 | (Hametner et al. 2014b) |
| *Gyalecta jenensis* | *Trentepohlia aurea* | JQ617916 | (Hametner et al. 2014a) |
| *Halegrapha mucronata* | *Trentepohlia* sp. | KC469259 | (Hametner et al. 2014b) |
| *Halegrapha mucronata* | *Printzina* sp*.* | KC469263 | (Hametner et al. 2014b) |
| *Halegrapha mucronata* | *Trentepohlia* sp. | KC469267 | (Hametner et al. 2014b) |
| *Halegrapha mucronata* | *Trentepohlia* sp. | KC469277 | (Hametner et al. 2014b) |
| *Herpothallon* aff*. echinatum* | *Printzina* cf*. lagenifera* | MT075979 | (Kosecka et al. 2020) |
| *Herpothallon* cf*. philippinum* | *Trentepohlia* sp. | MT075980 | (Kosecka et al. 2020) |
| *Herpothallon* cf*. roseocinctum* | *Trentepohlia* sp. | MT075995 | (Kosecka et al. 2020) |
| *Herpothallon* cf*. rubrocinctum* | *Trentepohlia* sp. | MT075981 | (Kosecka et al. 2020) |
| *Herpothallon cinereum* | *Printzina* cf*. lagenifera* | MT075983 | (Kosecka et al. 2020) |
| *Herpothallon cinereum* | *Trentepohlia* sp. | MT075984 | (Kosecka et al. 2020) |
| *Herpothallon cinereum* | *Trentepohlia* sp. | MT075985 | (Kosecka et al. 2020) |
| *Herpothallon confusum* | *Trentepohlia* sp. | MT075986 | (Kosecka et al. 2020) |
| *Herpothallon echinatum* | *Printzina* cf*. lagenifera* | MT075987 | (Kosecka et al. 2020) |
| *Herpothallon echinatum* | *Printzina* cf*. lagenifera* | MT075988 | (Kosecka et al. 2020) |
| *Herpothallon minimum* | *Trentepohlia* sp. | MT075989 | (Kosecka et al. 2020) |
| *Herpothallon philippinum* | *Trentepohlia* sp. | MT075990 | (Kosecka et al. 2020) |
| *Herpothallon philippinum* | *Trentepohlia* sp. | MT075991 | (Kosecka et al. 2020) |
| *Herpothallon philippinum* | *Trentepohlia* sp. | MT075992 | (Kosecka et al. 2020) |
| *Herpothallon philippinum* | *Trentepohlia* sp. | MT075993 | (Kosecka et al. 2020) |
| *Herpothallon pustulatum* | *Trentepohlia* sp. | MT075994 | (Kosecka et al. 2020) |
| *Herpothallon pustulatum* | *Trentepohlia* sp. | MT075996 | (Kosecka et al. 2020) |
| *Herpothallon roseocinctum* | *Trentepohlia* sp. | MT075982 | (Kosecka et al. 2020) |
| *Herpothallon roseocinctum* | *Trentepohlia* sp. | MT075997 | (Kosecka et al. 2020) |
| *Herpothallon roseocinctum* | *Trentepohlia* sp. | MT075998 | (Kosecka et al. 2020) |
| *Herpothallon roseocinctum* | *Trentepohlia* sp. | MT075999 | (Kosecka et al. 2020) |
| *Herpothallon roseocinctum* | *Trentepohlia* sp. | MT076000 | (Kosecka et al. 2020) |
| *Herpothallon roseocinctum* | *Trentepohlia* sp. | MT076001 | (Kosecka et al. 2020) |
| *Herpothallon rubrocinctum* | *Trentepohlia* sp. | KC469220 | (Hametner et al. 2014b) |
| *Herpothallon rubroechinatum* | *Printzina* cf*. lagenifera* | MT076002 | (Kosecka et al. 2020) |
| *Herpothallon* sp*.* | *Printzina* cf*. lagenifera* | KC469244 | (Hametner et al. 2014b) |
| *Herpothallon* sp*.* | *Trentepohlia* sp. | MT075953 | (Kosecka et al. 2020) |
| *Herpothallon* sp*.* | *Trentepohlia* sp. | MT075978 | (Kosecka et al. 2020) |
| *Herpothallon* sp*.* | *Trentepohlia* sp. | MT076003 | (Kosecka et al. 2020) |
| *Herpothallon* sp*.* | *Printzina lagenifera* | MT076004 | (Kosecka et al. 2020) |
| *Herpothallon* sp*.* | *Printzina lagenifera* | MT076005 | (Kosecka et al. 2020) |
| *Herpothallon* sp*.* | *Printzina lagenifera* | MT076006 | (Kosecka et al. 2020) |
| *Herpothallon* sp*.* | *Trentepohlia* sp. | MT076007 | (Kosecka et al. 2020) |
| *Isalonactis madagascariensis* | *Trentepohliaceae* sp. | OL956883 | (Borgato et al. 2022) |
| *Laurera sepulta* | *Trentepohliales* | GU549454 | (Nelsen et al. 2011) |
| *Lecanactis abietina* | *Trentepohliaceae* sp. | OL956852 | (Borgato et al. 2022) |
| *Lecanactis abietina* | *Trentepohliaceae* sp. | OL956858 | (Borgato et al. 2022) |
| *Lecanactis abietina* | *Trentepohliaceae* sp. | OL956861 | (Borgato et al. 2022) |
| *Lecanographa amylacea* | *Trentepohlia* sp. | MG676235 | (Ertz et al. 2018) |
| *Lecanographa amylacea* | *Trentepohlia* sp. | MG676236 | (Ertz et al. 2018) |
| *Lecanographa amylacea* | *Trentepohlia* sp. | MG676237 | (Ertz et al. 2018) |
| *Lecanographa amylacea* | *Trentepohlia* sp. | MG676238 | (Ertz et al. 2018) |
| *Lecanographa atlantica* | *Trentepohliaceae* sp. | OL956888 | (Borgato et al. 2022) |
| *Lecanographa gr lyncea* | *Trentepohliaceae* sp. | OL956897 | (Borgato et al. 2022) |
| *Leiorreuma hypomelaenum* | *Printzina* sp*.* | KC469264 | (Hametner et al. 2014b) |
| *Leucodecton glaucescens* | *Trentepohlia* sp. | MT076008 | (Kosecka et al. 2020) |
| *Mazosia carnea* | *Trentepohliaceae* sp. | OL956891 | (Borgato et al. 2022) |
| *Mazosia carnea* | *Trentepohliaceae* sp. | OL956892 | (Borgato et al. 2022) |
| *Mycoporum eschweileri* | *Printzina* cf*. lagenifera* | KC469251 | (Hametner et al. 2014b) |
| *Mycoporum lacteum* | *Trentepohlia* sp. | JQ617930 | (Hametner et al. 2014a) |
| *Mycoporum* sp*.* | *Trentepohlia* sp. | KC469211 | (Hametner et al. 2014b) |
| *Myriotrema glauculum* | *Trentepohlia* sp. | MT076009 | (Kosecka et al. 2020) |
| *Nadvornikia peninsulae* | *Trentepohliales* | GU549463 | (Nelsen et al. 2011) |
| *Ocellularia* aff*. africana* | *Trentepohlia* sp. | MT076012 | (Kosecka et al. 2020) |
| *Ocellularia* aff*. africana* | *Printzina* sp*.* | MT076013 | (Kosecka et al. 2020) |
| *Ocellularia* aff*. sorediigera* | *Trentepohlia* sp. | MT076014 | (Kosecka et al. 2020) |
| *Ocellularia* aff*. sorediigera* | *Printzina bosseae* | MT076015 | (Kosecka et al. 2020) |
| *Ocellularia* aff*. sorediigera* | *Trentepohlia* sp. | MT076016 | (Kosecka et al. 2020) |
| *Ocellularia* aff*. sorediigera* | *Trentepohlia* sp. | MT076017 | (Kosecka et al. 2020) |
| *Ocellularia* aff*. sorediigera* | *Trentepohlia* sp. | MT076018 | (Kosecka et al. 2020) |
| *Ocellularia* aff*. sorediigera* | *Trentepohlia* sp. | MT076019 | (Kosecka et al. 2020) |
| *Ocellularia* aff*. sorediigera* | *Trentepohlia* sp. | MT076020 | (Kosecka et al. 2020) |
| *Ocellularia* aff*. sorediigera* | *Trentepohlia* sp. | MT076021 | (Kosecka et al. 2020) |
| *Ocellularia erodens* | *Trentepohlia* sp. | MT076010 | (Kosecka et al. 2020) |
| *Ocellularia sorediigera* | *Trentepohlia* sp. | MT076022 | (Kosecka et al. 2020) |
| *Ocellularia* sp*.* | *Trentepohlia* sp. | MT076011 | (Kosecka et al. 2020) |
| *Ocellularia* sp*.* | *Trentepohlia* sp. | MT076023 | (Kosecka et al. 2020) |
| *Ocellularia* sp*.* | *Trentepohlia* sp. | MT076024 | (Kosecka et al. 2020) |
| *Ocellularia* sp*.* | *Trentepohlia* sp. | MT076025 | (Kosecka et al. 2020) |
| *Ocellularia* sp*.* | *Trentepohlia* sp. | MT076026 | (Kosecka et al. 2020) |
| *Ocellularia* sp*.* | *Trentepohlia* sp. | MT076027 | (Kosecka et al. 2020) |
| *Ocellularia* sp*.* | *Trentepohlia* sp. | MT076028 | (Kosecka et al. 2020) |
| *Opegrapha areniseda* | *Trentepohliaceae* sp. | OL956809 | (Borgato et al. 2022) |
| *Opegrapha lithyrga* | *Trentepohliaceae* sp. | OL956815 | (Borgato et al. 2022) |
| *Opegrapha niveoatra* | *Trentepohliaceae* sp. | OL956921 | (Borgato et al. 2022) |
| *Opegrapha vermicellifera* | *Trentepohliaceae* sp. | OL956925 | (Borgato et al. 2022) |
| *Opegrapha vermicellifera* | *Trentepohliaceae* sp. | OL956929 | (Borgato et al. 2022) |
| *Opegrapha vermicellifera* | *Trentepohliaceae* sp. | OL956932 | (Borgato et al. 2022) |
| *Opegrapha vulgata* | *Trentepohliaceae* sp. | OL956817 | (Borgato et al. 2022) |
| *Opegrapha vulgata* | *Trentepohliaceae* sp. | OL956831 | (Borgato et al. 2022) |
| *Opegrapha vulgata* | *Trentepohliaceae* sp. | OL956851 | (Borgato et al. 2022) |
| *Opegrapha vulgata* | *Trentepohliaceae* sp. | OL956872 | (Borgato et al. 2022) |
| *Opegrapha vulgata* | *Trentepohliaceae* sp. | OL956874 | (Borgato et al. 2022) |
| *Opegrapha vulgata* | *Trentepohliaceae* sp. | OL956875 | (Borgato et al. 2022) |
| *Opegrapha vulgata* | *Trentepohliaceae* sp. | OL956879 | (Borgato et al. 2022) |
| *Opegrapha vulgata* | *Trentepohliaceae* sp. | OL956905 | (Borgato et al. 2022) |
| *Opegrapha vulgata* | *Trentepohliaceae* sp. | OL956928 | (Borgato et al. 2022) |
| *Phaeographis colligata* | *Printzina* sp*.* | KC469266 | (Hametner et al. 2014b) |
| *Phaeographis inusta* | *Printzina* sp*.* | KC469213 | (Hametner et al. 2014b) |
| *Phaeographis inusta* | *Trentepohlia* sp. | KC469247 | (Hametner et al. 2014b) |
| *Phaeographis platycarpa* | *Trentepohlia* sp. | KC469268 | (Hametner et al. 2014b) |
| *Phaeographis* sp*.* | *Trentepohlia* sp. | KC469234 | (Hametner et al. 2014b) |
| *Phaeographis* sp*.* | *Printzina* sp*.* | KC469270 | (Hametner et al. 2014b) |
| *Phaeographis* sp*.* | *Printzina* sp*.* | KC469271 | (Hametner et al. 2014b) |
| *Phaeographis subdividens* | *Printzina* sp*.* | KC469215 | (Hametner et al. 2014b) |
| *Phaeographis subdividens* | *Trentepohlia* sp. | KC469216 | (Hametner et al. 2014b) |
| *Phaeographis subintricata* | *Trentepohlia* sp. | KC469262 | (Hametner et al. 2014b) |
| *Phaeotrema pachysp.orum* | *Trentepohliales* | GU549465 | (Nelsen et al. 2011) |
| *Platythecium hypoleptum* | *Trentepohliales* | GU549464 | (Nelsen et al. 2011) |
| *Platythecium hypoleptum* | *Trentepohlia* sp. | KC469257 | (Hametner et al. 2014b) |
| *Porina* aff*. dolichophora* | *Trentepohliales* | GU549458 | (Nelsen et al. 2011) |
| ***Porina* aff. *epiphylla*** | ***Trentepohliaceae* sp.** | **QB144592** | Present study |
| ***Porina* aff. *lucida* 1** | ***Trentepohliaceae* sp.** | **QB144562** | Present study |
| ***Porina* aff. *lucida* 1** | ***Trentepohliaceae* sp.** | **QB144564** | Present study |
| ***Porina* aff. *lucida* 1** | ***Trentepohliaceae* sp.** | **QB144566** | Present study |
| ***Porina* aff. *lucida* 1** | ***Trentepohliaceae* sp.** | **QB144568** | Present study |
| ***Porina* aff. *lucida* 1** | ***Trentepohliaceae* sp.** | **QB144580** | Present study |
| ***Porina* aff. *lucida* 1** | ***Trentepohliaceae* sp.** | **QB144595** | Present study |
| ***Porina* aff. *lucida* 2** | ***Trentepohliaceae* sp.** | **QB144559** | Present study |
| ***Porina* aff. *lucida* 2** | ***Trentepohliaceae* sp.** | **QB144579** | Present study |
| ***Porina* aff. *lucida* 3** | ***Trentepohliaceae* sp.** | **QB144589** | Present study |
| ***Porina* aff. *lucida* 3** | ***Trentepohliaceae* sp.** | **QB144590** | Present study |
| ***Porina* aff*. mazosioides*** | ***Trentepohliaceae* sp.** | **QB144496** | Present study |
| ***Porina* aff*. mazosioides*** | ***Trentepohliaceae* sp.** | **QB144497** | Present study |
| ***Porina* aff. s*ubepiphylla* 1** | ***Trentepohliaceae* sp.** | **QB144553** | Present study |
| ***Porina* aff. s*ubepiphylla* 1** | ***Trentepohliaceae* sp.** | **QB144555** | Present study |
| ***Porina* aff. s*ubepiphylla* 1** | ***Trentepohliaceae* sp.** | **QB144556** | Present study |
| ***Porina* aff. s*ubepiphylla* 2** | ***Trentepohliaceae* sp.** | **QB144552** | Present study |
| ***Porina* aff. s*ubepiphylla* 2** | ***Trentepohliaceae* sp.** | **QB144582** | Present study |
| ***Porina* cf. *atriceps*** | ***Trentepohliaceae* sp.** | **QB144569** | Present study |
| ***Porina* cf. *atriceps*** | ***Trentepohliaceae* sp.** | **QB144570** | Present study |
| ***Porina* cf. *atriceps*** | ***Trentepohliaceae* sp.** | **QB144571** | Present study |
| ***Porina* cf. *australiensis*** | ***Trentepohliaceae* sp.** | **QB144572** | Present study |
| ***Porina* cf. *epiphylla* 1** | ***Trentepohliaceae* sp.** | **QB144554** | Present study |
| ***Porina* cf. *epiphylla* 1** | ***Trentepohliaceae* sp.** | **QB144581** | Present study |
| ***Porina* cf. *epiphylla* 2** | ***Trentepohliaceae* sp.** | **QB144593** | Present study |
| ***Porina* cf*. epiphylloides*** | ***Trentepohliaceae* sp.** | **QB144541** | Present study |
| ***Porina* cf. *karnatakensis* 1** | ***Trentepohliaceae* sp.** | **QB144583** | Present study |
| ***Porina* cf. *karnatakensis* 1** | ***Trentepohliaceae* sp.** | **QB144584** | Present study |
| ***Porina* cf. *karnatakensis* 1** | ***Trentepohliaceae* sp.** | **QB144585** | Present study |
| ***Porina* cf. *karnatakensis* 1** | ***Trentepohliaceae* sp.** | **QB144586** | Present study |
| ***Porina* cf. *karnatakensis* 2** | ***Trentepohliaceae* sp.** | **QB144560** | Present study |
| ***Porina* cf. *karnatakensis* 2** | ***Trentepohliaceae* sp.** | **QB144565** | Present study |
| ***Porina* cf. *karnatakensis* 2** | ***Trentepohliaceae* sp.** | **QB144573** | Present study |
| ***Porina* cf. *leptosperma*** | ***Trentepohliaceae* sp.** | **QB144538** | Present study |
| ***Porina* cf. *lucida* 1** | ***Trentepohliaceae* sp.** | **QB144551** | Present study |
| ***Porina* cf. *lucida* 1** | ***Trentepohliaceae* sp.** | **QB144558** | Present study |
| ***Porina* cf. *lucida* 1** | ***Trentepohliaceae* sp.** | **QB144574** | Present study |
| ***Porina* cf. *lucida* 2** | ***Trentepohliaceae* sp.** | **QB144561** | Present study |
| *Porina* cf*. scabrida* | *Trentepohlia* sp*.* | OR053627 | (Sanders et al. 2023) |
| ***Porina complanata*** | ***Trentepohliaceae* sp.** | **QB144550** | Present study |
| ***Porina complanata*** | ***Trentepohliaceae* sp.** | **QB144557** | Present study |
| ***Porina complanata*** | ***Trentepohliaceae* sp.** | **QB144567** | Present study |
| ***Porina complanata*** | ***Trentepohliaceae* sp.** | **QB144576** | Present study |
| ***Porina complanata*** | ***Trentepohliaceae* sp.** | **QB144577** | Present study |
| ***Porina complanata*** | ***Trentepohliaceae* sp.** | **QB144587** | Present study |
| ***Porina complanata*** | ***Trentepohliaceae* sp.** | **QB144588** | Present study |
| *Porina distans* | *Trentepohliales* | GU549456 | (Nelsen et al. 2011) |
| *Porina dolichophora* | *Trentepohliales* | GU549453 | (Nelsen et al. 2011) |
| *Porina farinosa* | *Trentepohliales* | GU549444 | (Nelsen et al. 2011) |
| *Porina hibernica* | *Trentepohlia* sp. | OR053633 | (Sanders et al. 2023) |
| *Porina hibernica* | *Trentepohlia* sp. | OR053634 | (Sanders et al. 2023) |
| *Porina hibernica* | *Trentepohlia* sp. | OR053635 | (Sanders et al. 2023) |
| *Porina imitatrix* | *Trentepohliales* | GU549455 | (Nelsen et al. 2011) |
| *Porina leptalea* | *Trentepohliaceae* sp. | OL956881 | (Borgato et al. 2022) |
| ***Porina limbulata*** | ***Trentepohliaceae* sp.** | **QB144519** | Present study |
| ***Porina madagacariensis*** | ***Trentepohliaceae* sp.** | **QB144563** | Present study |
| ***Porina mayottensis*** | ***Trentepohliaceae* sp.** | **QB144493** | Present study |
| ***Porina mayottensis*** | ***Trentepohliaceae* sp.** | **QB144494** | Present study |
| ***Porina mayottensis*** | ***Trentepohliaceae* sp.** | **QB144495** | Present study |
| ***Porina mayottensis*** | ***Trentepohliaceae* sp.** | **QB144543** | Present study |
| ***Porina mayottensis*** | ***Trentepohliaceae* sp.** | **QB144544** | Present study |
| ***Porina mazosioides*** | ***Trentepohliaceae* sp.** | **QB144500** | Present study |
| ***Porina mazosioides*** | ***Trentepohliaceae* sp.** | **QB144501** | Present study |
| ***Porina mazosioides*** | ***Trentepohliaceae* sp.** | **QB144504** | Present study |
| ***Porina mazosioides*** | ***Trentepohliaceae* sp.** | **QB144506** | Present study |
| ***Porina mazosioides*** | ***Trentepohliaceae* sp.** | **QB144507** | Present study |
| ***Porina mazosioides*** | ***Trentepohliaceae* sp.** | **QB144508** | Present study |
| ***Porina mazosioides*** | ***Trentepohliaceae* sp.** | **QB144509** | Present study |
| ***Porina mazosioides*** | ***Trentepohliaceae* sp.** | **QB144510** | Present study |
| ***Porina mazosioides*** | ***Trentepohliaceae* sp.** | **QB144511** | Present study |
| ***Porina mazosioides*** | ***Trentepohliaceae* sp.** | **QB144524** | Present study |
| ***Porina mazosioides*** | ***Trentepohliaceae* sp.** | **QB144526** | Present study |
| ***Porina mazosioides*** | ***Trentepohliaceae* sp.** | **QB144531** | Present study |
| *Porina microcoralloides* | *Trentepohlia* sp. | OR053611 | (Sanders et al. 2023) |
| *Porina microcoralloides* | *Trentepohlia* sp. | OR053612 | (Sanders et al. 2023) |
| *Porina microcoralloides* | *Trentepohlia* sp. | OR053613 | (Sanders et al. 2023) |
| *Porina microcoralloides* | *Trentepohlia* sp. | OR053614 | (Sanders et al. 2023) |
| *Porina microcoralloides* | *Trentepohlia* sp. | OR053617 | (Sanders et al. 2023) |
| *Porina microcoralloides* | *Trentepohlia* sp*.* | OR053619 | (Sanders et al. 2023) |
| *Porina microcoralloides* | *Trentepohlia* sp*.* | OR053621 | (Sanders et al. 2023) |
| *Porina microcoralloides* | *Trentepohlia* sp*.* | OR053622 | (Sanders et al. 2023) |
| *Porina microcoralloides* | *Trentepohlia* sp*.* | OR053623 | (Sanders et al. 2023) |
| *Porina microcoralloides* | *Trentepohlia* sp*.* | OR053628 | (Sanders et al. 2023) |
| *Porina microcoralloides* | *Trentepohlia* sp*.* | OR053629 | (Sanders et al. 2023) |
| *Porina microcoralloides* | *Trentepohlia* sp*.* | OR053630 | (Sanders et al. 2023) |
| *Porina microcoralloides* | *Trentepohlia* sp. | OR053631 | (Sanders et al. 2023) |
| *Porina nanoarbuscula* | *Trentepohlia* sp. | OR053615 | (Sanders et al. 2023) |
| *Porina nanoarbuscula* | *Trentepohlia* sp. | OR053616 | (Sanders et al. 2023) |
| *Porina nanoarbuscula* | *Trentepohlia* sp*.* | OR053618 | (Sanders et al. 2023) |
| *Porina nanoarbuscula* | *Trentepohlia* sp*.* | OR053624 | (Sanders et al. 2023) |
| *Porina nanoarbuscula* | *Trentepohlia* sp*.* | OR053625 | (Sanders et al. 2023) |
| *Porina nanoarbuscula* | *Trentepohlia* sp*.* | OR053626 | (Sanders et al. 2023) |
| *Porina nucula* | *Trentepohliales* | GU549457 | (Nelsen et al. 2011) |
| ***Porina parva*** | ***Trentepohliaceae* sp.** | **QB144498** | Present study |
| ***Porina parva*** | ***Trentepohliaceae* sp.** | **QB144502** | Present study |
| ***Porina parva*** | ***Trentepohliaceae* sp.** | **QB144532** | Present study |
| ***Porina parva*** | ***Trentepohliaceae* sp.** | **QB144533** | Present study |
| ***Porina parva*** | ***Trentepohliaceae* sp.** | **QB144542** | Present study |
| ***Porina parva*** | ***Trentepohliaceae* sp.** | **QB144547** | Present study |
| ***Porina parva*** | ***Trentepohliaceae* sp.** | **QB144548** | Present study |
| ***Porina parva*** | ***Trentepohliaceae* sp.** | **QB144549** | Present study |
| *Porina pseudohibernica* | *Trentepohlia* sp. | OR053632 | (Sanders et al. 2023) |
| ***Porina rufula*** | ***Trentepohliaceae* sp.** | **QB144499** | Present study |
| ***Porina rufula*** | ***Trentepohliaceae* sp.** | **QB144534** | Present study |
| ***Porina rufula*** | ***Trentepohliaceae* sp.** | **QB144535** | Present study |
| *Porina sp.* | *Trentepohlia* sp. | KC469202 | (Hametner et al. 2014b) |
| *Porina sp.* | *Trentepohlia* cf*. jolithus* | MT076029 | (Kosecka et al. 2020) |
| *Porina sp.* | *Trentepohlia* cf*. umbrina* | MT076030 | (Kosecka et al. 2020) |
| ***Porina* sp. 1** | ***Trentepohliaceae* sp.** | **QB144578** | Present study |
| ***Porina* sp. 2** | ***Trentepohliaceae* sp.** | **QB144594** | Present study |
| ***Porina* sp. 3** | ***Trentepohliaceae* sp.** | **QB144575** | Present study |
| ***Porina* sp. 4** | ***Trentepohliaceae* sp.** | **QB144591** | Present study |
| *Porina sp..* | *Trentepohlia* sp*.* | OR053620 | (Sanders et al. 2023) |
| ***Porina sphaerocephala*** | ***Trentepohliaceae* sp.** | **QB144545** | Present study |
| ***Porina sphaerocephaloides*** | ***Trentepohliaceae* sp.** | **QB144505** | Present study |
| ***Porina sphaerocephaloides*** | ***Trentepohliaceae* sp.** | **QB144512** | Present study |
| ***Porina sphaerocephaloides*** | ***Trentepohliaceae* sp.** | **QB144514** | Present study |
| ***Porina sphaerocephaloides*** | ***Trentepohliaceae* sp.** | **QB144515** | Present study |
| ***Porina sphaerocephaloides*** | ***Trentepohliaceae* sp.** | **QB144517** | Present study |
| ***Porina sphaerocephaloides*** | ***Trentepohliaceae* sp.** | **QB144521** | Present study |
| ***Porina sphaerocephaloides*** | ***Trentepohliaceae* sp.** | **QB144523** | Present study |
| ***Porina sphaerocephaloides*** | ***Trentepohliaceae* sp.** | **QB144527** | Present study |
| ***Porina sphaerocephaloides*** | ***Trentepohliaceae* sp.** | **QB144528** | Present study |
| ***Porina sphaerocephaloides*** | ***Trentepohliaceae* sp.** | **QB144529** | Present study |
| ***Porina sphaerocephaloides*** | ***Trentepohliaceae* sp.** | **QB144530** | Present study |
| ***Porina sphaerocephaloides*** | ***Trentepohliaceae* sp.** | **QB144536** | Present study |
| ***Porina sphaerocephaloides*** | ***Trentepohliaceae* sp.** | **QB144537** | Present study |
| ***Porina sphaerocephaloides*** | ***Trentepohliaceae* sp.** | **QB144539** | Present study |
| ***Porina sphaerocephaloides*** | ***Trentepohliaceae* sp.** | **QB144540** | Present study |
| ***Porina sphaerocephaloides*** | ***Trentepohliaceae* sp.** | **QB144546** | Present study |
| *Porina tetracerae* | *Trentepohliales* | GU549445 | (Nelsen et al. 2011) |
| *Porina tetracerae* | *Trentepohliales* | GU549446 | (Nelsen et al. 2011) |
| *Pseudoschismatomma rufescens* | *Trentepohliaceae* sp. | OL956810 | (Borgato et al. 2022) |
| *Pyrenula laevigata* | *Printzina* sp*.* | JQ617913 | (Hametner et al. 2014a) |
| *Pyrenula laevigata* | *Printzina* sp*.* | JQ617935 | (Hametner et al. 2014a) |
| *Pyrenula quassiicola* | *Trentepohlia* sp. | KC469281 | (Hametner et al. 2014b) |
| *Pyrenula thelomorpha* | *Trentepohlia* sp. | KC469204 | (Hametner et al. 2014b) |
| *Reichlingia leopoldii* | *Trentepohliaceae* sp. | OL956814 | (Borgato et al. 2022) |
| *Reichlingia leopoldii* | *Trentepohliaceae* sp. | OL956840 | (Borgato et al. 2022) |
| *Roccella* cf*. montagnei* | *Trentepohlia* sp. | JQ617923 | (Hametner et al. 2014a) |
| *Roccella decipiens* | *Trentepohlia* sp. | JQ617920 | (Hametner et al. 2014a) |
| *Roccella galapagoensis* | *Trentepohlia* sp. | JQ617921 | (Hametner et al. 2014a) |
| *Roccella linearis* | *Trentepohlia* sp. | JQ617924 | (Hametner et al. 2014a) |
| *Roccella lirellina* | *Trentepohlia* sp. | JQ617918 | (Hametner et al. 2014a) |
| *Roccella maderensis* | *Trentepohlia* sp. | JQ617925 | (Hametner et al. 2014a) |
| *Roccella phycopsis* | *Trentepohlia* sp. | JQ617922 | (Hametner et al. 2014a) |
| *Roccella phycopsis* | *Trentepohliaceae* sp. | OL956910 | (Borgato et al. 2022) |
| *Roccella phycopsis* | *Trentepohliaceae* sp. | OL956912 | (Borgato et al. 2022) |
| *Roccella phycopsis* | *Trentepohliaceae* sp. | OL956915 | (Borgato et al. 2022) |
| *Roccella phycopsis* | *Trentepohliaceae* sp. | OL956916 | (Borgato et al. 2022) |
| *Roccella phycopsis* | *Trentepohliaceae* sp. | OL956917 | (Borgato et al. 2022) |
| *Roccella phycopsis* | *Trentepohliaceae* sp. | OL956918 | (Borgato et al. 2022) |
| *Roccella phycopsis* | *Trentepohliaceae* sp. | OL956920 | (Borgato et al. 2022) |
| *Roccella phycopsis* | *Trentepohliaceae* sp. | OL956923 | (Borgato et al. 2022) |
| *Roccella tinctoria* | *Trentepohlia* sp. | JQ617919 | (Hametner et al. 2014a) |
| *Roccella tinctoria* | *Trentepohlia* sp. | JQ617926 | (Hametner et al. 2014a) |
| *Roccella tinctoria* | *Trentepohlia* sp. | JQ617927 | (Hametner et al. 2014a) |
| *Roccella tuberculata* | *Trentepohliaceae* sp. | OL956913 | (Borgato et al. 2022) |
| *Roccellographa circumscripta* | *Trentepohliaceae* sp. | OL956886 | (Borgato et al. 2022) |
| *Sagenidiopsis isidiata* | *Trentepohlia* sp. | MT076031 | (Kosecka et al. 2020) |
| *Sagenidiopsis isidiata* | *Trentepohlia* sp. | MT076032 | (Kosecka et al. 2020) |
| *Sagenidiopsis* sp*.* | *Printzina lagenifera* | MT076033 | (Kosecka et al. 2020) |
| *Sarcographa cinchonarum* | *Trentepohlia* sp. | KC469236 | (Hametner et al. 2014b) |
| *Schizotrema sp.* | *Trentepohlia* sp. | MT076034 | (Kosecka et al. 2020) |
| *Sclerophyton trinidadense* | *Trentepohliaceae* sp. | OL956908 | (Borgato et al. 2022) |
| *Sparria endlicheri* | *Trentepohliaceae* sp. | OL956821 | (Borgato et al. 2022) |
| *Sparria endlicheri* | *Trentepohliaceae* sp. | OL956890 | (Borgato et al. 2022) |
| *Sporodophoron cretaceum* | *Trentepohliaceae* sp. | OL956902 | (Borgato et al. 2022) |
| *Sporodophoron cretaceum* | *Trentepohliaceae* sp. | OL956903 | (Borgato et al. 2022) |
| *Sporodophoron cretaceum* | *Trentepohliaceae* sp. | OL956904 | (Borgato et al. 2022) |
| *Strigula smaragdula* | *Printzina* cf*. lagenifera* | KC469245 | (Hametner et al. 2014b) |
| *Strigula smaragdula* | *Cephaleuros virescens* | KC469252 | (Hametner et al. 2014b) |
| *Syncesia farinacea* | *Trentepohlia* sp. | MT076035 | (Kosecka et al. 2020) |
| *Syncesia farinacea* | *Trentepohlia* sp. | MT076036 | (Kosecka et al. 2020) |
| *Syncesia leprobola* | *Trentepohliaceae* sp. | OL956933 | (Borgato et al. 2022) |
| *Syncesia leprobola* | *Trentepohliaceae* sp. | OL956934 | (Borgato et al. 2022) |
| *Syncesia myrticola* | *Trentepohliaceae* sp. | OL956893 | (Borgato et al. 2022) |
| *Thalloloma deplanatum* | *Trentepohlia* sp. | KC469278 | (Hametner et al. 2014b) |
| *Thelotrema lepadinum* | *Trentepohlia* sp. | JQ617928 | (Hametner et al. 2014a) |
| *Thelotrema lepadinum* | *Trentepohlia* sp. | KC469207 | (Hametner et al. 2014b) |
| *Thelotrema lepadinum* | *Trentepohlia* sp. | KC469248 | (Hametner et al. 2014b) |
| *Thelotrema lepadinum* | *Trentepohlia* sp. | KC469253 | (Hametner et al. 2014b) |
| *Thelotrema lepadinum* | *Trentepohlia* sp. | KC469254 | (Hametner et al. 2014b) |
| *Thelotrema lepadinum* | *Trentepohlia* sp. | KC469255 | (Hametner et al. 2014b) |
| *Thelotrema* sp*.* | *Trentepohlia* sp. | MT075945 | (Kosecka et al. 2020) |
| ***Trichothelium* cf. *epiphyllum*** | ***Trentepohliaceae* sp.** | **QB144503** | Present study |
| ***Trichothelium* cf. *epiphyllum*** | ***Trentepohliaceae* sp.** | **QB144513** | Present study |
| ***Trichothelium* cf. *epiphyllum*** | ***Trentepohliaceae* sp.** | **QB144516** | Present study |
| ***Trichothelium* cf. *epiphyllum*** | ***Trentepohliaceae* sp.** | **QB144520** | Present study |
| ***Trichothelium* cf. *epiphyllum*** | ***Trentepohliaceae* sp.** | **QB144522** | Present study |
| ***Trichothelium* cf. *epiphyllum*** | ***Trentepohliaceae* sp.** | **QB144525** | Present study |
| ***Trichothelium* sp.** | ***Trentepohliaceae* sp.** | **QB144518** | Present study |
| *Trypethelium eluteriae* | *Trentepohliales* | GU549459 | (Nelsen et al. 2011) |
| *Tylophoron hibernicum* | *Trentepohlia* sp. | MT076037 | (Kosecka et al. 2020) |
| *Tylophoron hibernicum* | *Trentepohlia* sp. | MT076038 | (Kosecka et al. 2020) |

**References**

Bartoli F, Ellwood NTW, Bruno L et al. (2019) Ecological and taxonomic characterisation of *Trentepohlia umbrina* (Kützing) Bornet growing on stone surfaces in Lazio (Italy). Annals of Microbiology 69: 1059–1070. https://doi.org/10.1007/s13213-019-01472-9

Borgato L, Ertz D, Van Rossum F et al. (2022) The diversity of lichenized trentepohlioid algal (*Ulvophyceae*) communities is driven by fungal taxonomy and ecological fac­tors. Journal of Phycology 58: 582–602. <https://doi.org/10.1111/jpy.13252>

Cocquyt E, Verbruggen H, Leliaert F et al. (2009) Gain and loss of elongation factor genes in green algae. BMC Evolutionary Biology 9: 39. https://doi.org/10.1186/1471-2148-9-39

Ertz D, Guzow-Krzemińska B, Thor G et al. (2018) Photobiont switching causes changes in the reproduction strategy and phenotypic dimorphism in the *Arthoniomycetes*. Sci­entific Reports 8: 4952. https://doi.org/10.1038/s41598-018-23219-3

Fučíková K, Leliaert F, Cooper ED et al. (2014) New phylogenetic hypotheses for the core *Chlorophyta* based on chloroplast sequence data. Frontiers in Ecology and Evolution 2: 63. https://doi.org/10.3389/fevo.2014.00063

Hametner C, Stocker-Wörgötter E, Grube M (2014a) New insights into diversity and se­lectivity of trentepohlialean lichen photobionts from the extratropics. Symbiosis 63: 31–40. https://doi.org/10.1007/s13199-014-0285-z

Hametner C, Stocker-Wörgötter E, Rindi F et al. (2014b) Phylogenetic position and morphol­ogy of lichenized *Trentepohliales* (*Ulvophyceae*, *Chlorophyta*) from selected species of *Graphidaceae*. Phycological Research 62: 170–186. <https://doi.org/10.1111/pre.12055>

Klimešová M, Rindi F, Škaloud P (2019) DNA cloning demonstrates high genetic het­erogeneity in populations of the subaerial green alga *Trentepohlia* (*Trentepohliales*, *Chlorophyta*). Journal of Phycology 55: 224–235. https://doi.org/10.1111/jpy.12817

Kosecka M, Jabłońska A, Flakus A et al. (2020) Trentepohlialean algae (*Trentepohliales*, *Ulvophyceae*) show preference to selected mycobiont lineages in lichen symbioses. Journal of Phycology 56: 979–993. https://doi.org/10.1111/jpy.12994

Lam DW, Zechman FW (2006) Phylogenetic analyses of the *Bryopsidales* (*Ulvophyceae*, *Chlorophyta*) based on rubisco large subunit gene sequences. Journal of Phycology 42: 669–678. <https://doi.org/10.1111/j.1529-8817.2006.00230.x>

Lemes-Da-Silva NM, López-Bautista JM, Branco LHZ (2018) A new and cryptic species of *Trentepohlia* (*Ulvophyceae*, *Chlorophyta*) from Brazilian mixed forests. Phycologia 57: 385–393. <https://doi.org/10.2216/16-133.1>

Nelsen MP, Plata ER, Andrew CJ et al. (2011) Phylogenetic diversity of Trentepohlialean algae associated with lichen-forming fungi. Journal of Phycology 47: 282–290. https://doi.org/10.1111/j.1529-8817.2011.00962.x

Ramya M, Ponmurugan P, Saravanan D (2012) Management of *Cephaleuros parasiticus* Karst (*Trentepohliales*: *Trentepohliaceae*), an algal pathogen of tea plant, *Camellia sinsensis* (L) (O. Kuntze). Crop Protection 44: 66–74. https://doi.org/10.1016/j.cropro.2012.10.023

Rindi F, Lam DW, López-Bautista JM (2009) Phylogenetic relationships and spe­cies circumscription in *Trentepohlia* and *Printzina* (*Trentepohliales*, *Chlorophyta*). Molecular Phylogenetics and Evolution 52: 329–339. https://doi.org/10.1016/j. ympev.2009.01.009

Sanders W, De Carolis R, Ertz D et al. (2023) Independent, structurally distinct transi­tions to microfruticose growth in the crustose genus *Porina* (*Ostropales*, *Lecanoro­mycetes*): new isidioid species from south-western Florida. The Lichenologist 55: 347–365. https://doi.org/10.1017/s0024282923000440

Shimada S, Hiraoka M, Nabata S et al. (2003) Molecular phylogenetic analyses of the Japanese *Ulva* and *Enteromorpha* (*Ulvales*, *Ulvophyceae*), with special reference to the free-floating *Ulva*. Phycological Research 51: 99–108. https://doi.org/10.1046/ j.1440-1835.2003.00296.x
